# Real-time electrical monitoring of enzymatic catalytic dynamics at the single-molecule level

**DOI:** 10.1038/s41467-026-74020-0

**Published:** 2026-06-05

**Authors:** Zhimin Fan, Zusen Chen, Zhengwen Gong, Sanjun Shi, Mingdi Xu, Xiaonan Feng, Yulu Liu, Ya Hu, Xiaoduo Chen, Guomao Zheng, Bintian Zhang

**Affiliations:** 1 Shenzhen Key Laboratory of Precision Measurement and Early Warning Technology for Urban Environmental Health Risks, School of Environmental Science and Engineering, Southern University of Science and Technology, Shenzhen, China; 2 Key Laboratory of Systems Health Science of Zhejiang Province, School of Life Science, Hangzhou Institute for Advanced Study, University of Chinese Academy of Sciences, Hangzhou, China

**Keywords:** Analytical biochemistry, Single-molecule biophysics, Environmental chemistry, Enzymes

## Abstract

Monitoring enzyme structural dynamics is essential for elucidating catalytic mechanisms, yet transient conformational fluctuations on microsecond-to-millisecond timescales remain challenging to resolve with conventional techniques. Here, we investigate the catalytic dynamics of cytochrome P450 1A1 (CYP1A1) during benzo[a]pyrene (BaP) metabolism by measuring single-molecule protein conductance. We show that catalysis-induced α-helix structural rearrangements, together with redox transitions of the heme center, modulate charge-transport efficiency. A negative correlation between BaP concentration and conductance enables construction of a kinetic model, yielding an apparent Michaelis constant of 24.2-43.2 μM. Real-time conductance measurements resolve four distinct conductance states associated with catalytic intermediates, which are further assigned using metabolic intermediates as substrates. These results provide insight into competing detoxification and activation pathways of BaP metabolism. This work establishes protein conductance as a generalizable platform for probing transient enzymatic dynamics and kinetics at the single-molecule level.

## Abstract (web summary)

Monitoring structural changes in enzymes is essential for elucidating the mechanisms and dynamics of enzymatic catalysis, however, the transient conformational fluctuations involved in catalytic processes typically occur on microsecond-to-millisecond timescales, posing significant challenges for detection via conventional techniques. Here, the authors investigate the catalytic dynamics of cytochrome P450 1A1 during the metabolism of benzo[a]pyrene by measuring the electrical conductance across a single enzyme molecule.

## Introduction

Enzyme-mediated biocatalysis plays a crucial role in numerous biological processes, including DNA replication, protein synthesis, and metabolic pathways^1–3^. A typical enzymatic catalysis cycle involves substrate recognition and binding, induced fit, chemical conversion, and product release, each accompanied by conformational changes in the enzyme^4,5^. Therefore, monitoring the periodic structural transitions of enzymes during catalysis is essential for understanding reaction dynamics and the underlying mechanisms^6^. However, these conformational fluctuations are often transient, occurring on microsecond timescales, which poses significant challenges for their real-time observation^7^. Moreover, the structural changes are typically subtle and difficult to detect directly^8^. As such, techniques with exceptionally high temporal resolution and sensitivity are required to monitor enzyme conformational dynamics.

Conventional methods for studying protein structures include circular dichroism (CD) spectroscopy, nuclear magnetic resonance (NMR), X-ray crystallography, and cryo-electron microscopy (Cryo-EM). CD spectroscopy quantifies the secondary structure composition of proteins (e.g., α helix/β-sheet)^9,10^; however, as a bulk measurement technique, it yields ensemble-averaged results that obscure molecular-level heterogeneity. NMR spectroscopy enables real-time observation of conformational dynamics in solutions without the need for crystallization, but it suffers from low sensitivity and often requires isotopic labeling^9^. X-ray crystallography and Cryo-EM offer atomic-resolution, three-dimensional structures of proteins, yet both primarily provide static snapshots and are limited in capturing dynamic transitions^11^. To elucidate the conformational motions underlying enzymatic catalytic steps, a method with high spatiotemporal resolution and single-molecule sensitivity is essential. In recent years, several single-molecule detection techniques have been developed to resolve complex biological dynamics. For instance, single-molecule force spectroscopy has been used to characterize transient interactions between photosynthetic reaction centers and electron donors^12^. Single-molecule fluorescence resonance energy transfer enables real-time tracking of conformational changes in biomolecules^13^. Nanopore-based sensing approaches have also been employed to examine interactions between proteins and DNA^14^. Furthermore, high-speed AFM provides exceptionally high spatiotemporal resolution, enabling the direct, real-time observation of protein dynamic processes under near-physiological conditions^15–17^. Despite these advancements, capturing enzyme catalytic dynamics remains a significant challenge due to the intrinsic complexity of catalytic processes, the transient and heterogeneous nature of intermediate states, and the intricate coupling between substrate and cofactor binding and catalytic turnover^18^.

Metalloproteins have been demonstrated to exhibit electrical conductance, a property that can also be observed in non-redox-active proteins when they are appropriately wired to metal electrodes through chemical connections^19,20^. One of the most intriguing aspects of protein conductance is its sensitivity to conformational changes induced by ligand binding or substrate recognition^21–23^. This property has significant technological implications, as functional proteins, such as antibodies, enzymes, and receptors, can be integrated into electric circuits to develop sensors for diverse applications^24^. These systems enable electrical signal readouts with high temporal resolution, making them particularly well-suited for probing the transient and subtle conformational changes of proteins that occur during biological processes. Enzymes often contain redox-active cofactors that are essential for catalytic function^25^. Conductance measurements of metalloproteins such as Azurin have revealed that charge transport can occur either through the peptide matrix via tunneling or through the metal center via hopping^26^. This dual mechanism may explain our previous observation of large conductance fluctuations upon activation of DNA polymerase^27^. Moreover, substrate binding or coenzyme association with an enzyme significantly alters the conductance through single-molecule protein junctions, offering a sensitive marker for monitoring enzymatic activity^27,28^. As such, in principle and practice, it is feasible to elucidate enzymatic catalytic mechanisms based on protein conductance measurements^29^. Nevertheless, the precise roles of cofactors, coenzymes, and conformational change in charge transport during catalysis remain poorly understood, and it remains technically challenging to resolve the complex, transient steps involved in enzymatic processes.

In this study, we investigate the conformational changes and catalytic dynamics of CYP1A1, a critical hepatic enzyme involved in the metabolism of polycyclic aromatic hydrocarbons, such as BaP, by monitoring its electrical conductance using a scanning tunneling microscope (STM). We demonstrate that the conductance of the enzyme is effectively modulated by coenzyme-mediated reduction of the heme iron (Fe^3+^) and substrate-induced conformational rearrangements. By combining single-molecule conductance measurements with CD spectroscopy and theoretical calculations, we establish a direct correlation between protein secondary structure and charge transport efficiency. Furthermore, real-time monitoring of conductance fluctuations during enzymatic catalysis resolves four distinct conductance states associated with transient catalytic intermediates, enabling the construction of a high-resolution kinetic map of competing detoxification and activation pathways in BaP metabolism. This work demonstrates a generalizable bioelectronic strategy for probing transient enzymatic dynamics at the single-molecule level, offering a powerful complement to conventional structural, spectroscopic, and imaging techniques.

## Results

### Single-molecule conductance measurement of CYP1A1

**Fig. 1:**
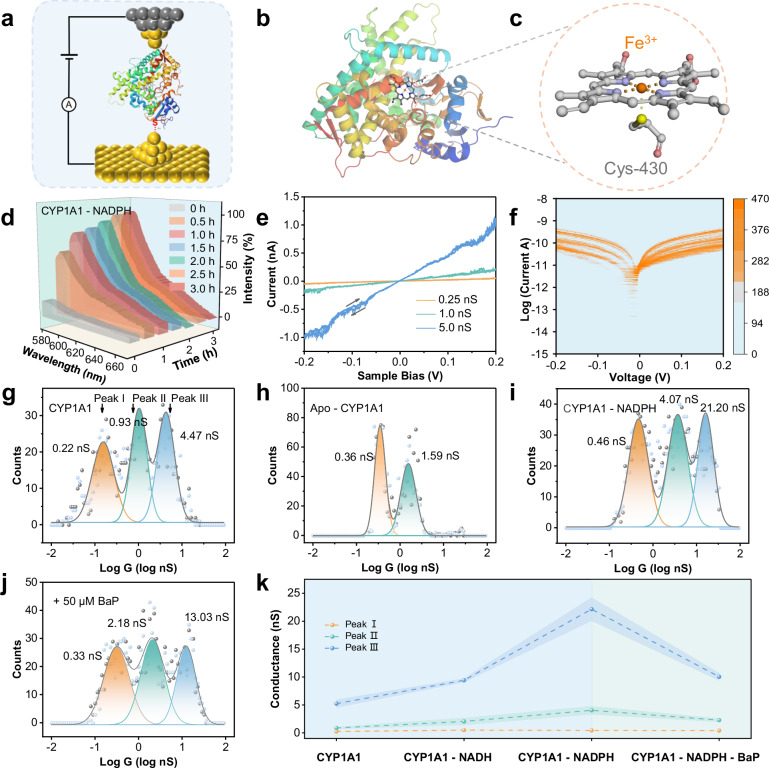
Electrical characterization of CYP1A1 via single-molecule conductance measurements using STM. **a** Schematic illustration of the single-molecule conductance measurement setup. CYP1A1 is covalently immobilized onto MPA-modified gold substrate via EDC/NHS coupling, while the probe remains unmodified and interacts with the enzyme through nonspecific contact or surface-exposed cysteine residues. **b** Crystal structure of CYP1A1 (PDB: 4I8V). **c** Molecular structure of the heme catalytic center in CYP1A1. **d** Enzymatic activity of CYP1A1 confirmed by fluorescence detection of resorufin, the product of CYP1A1-catalyzed deethylation of 7-ethoxyresorufin (7-ER). **e** Representative *I*–*V* curves recorded from a single CYP1A1 molecular junction, exhibiting linear and symmetric characteristics with overlapping forward and reverse scans. **f** Two-dimensional current–voltage map of CYP1A1, generated from logarithmic transformation of ~1000 *I*–*V* curves. **g**–**j** Conductance histograms of CYP1A1, apo-CYP1A1, CYP1A1-NADPH, and CYP1A1-NADPH-BaP (50 μM). **k** Summary of CYP1A1 conductance peak values under various conditions with coenzymes and substrate (data are presented as mean ± s.d., *n* = 3). Source data are provided as a Source data file.

CYP1A1 is a key enzyme involved in the metabolic detoxification process of living organisms and contains a ferric ion at its active center (Figs. 1b, c, and S1). The enzymatic activity was verified using UV–vis and fluorescence spectroscopy, yielding a specific activity of 1.9 U/mg (Figs. 1d and S2–S5). For conductance measurement, CYP1A1 was immobilized onto a mercaptopropionic acid (MPA)-modified gold substrate via EDC/NHS coupling chemistry (see “Methods” and Fig. S6). Successful surface functionalization was confirmed by Fourier-transform infrared (FTIR) spectroscopy, which revealed characteristic amide I and II absorption bands (Fig. S7), and by X-ray photoelectron spectroscopy (XPS), which identified elemental signatures of N and Fe (Fig. S8). Ellipsometry analysis showed a protein layer thickness of approximately 6.4 nm (Fig. S9), consistent with the crystallographic dimensions of CYP1A1 (PDB: 4I8V), suggesting the formation of a uniform monolayer on the gold substrate.

Conductance measurements of CYP1A1 were performed following our previous study^22^. Briefly, a fixed gap of ~4.5 nm was created between the CYP1A1-modified substrate and an insulated gold probe using an electrochemical STM in 50 mM phosphate-buffer saline (PBS) (Figs. 1a and S10). Upon bridging of a single protein molecule, indicated by a current increase exceeding 50 pA, bias voltage sweeps were performed from −0.2 to 0.2 V and then reversed at a scan rate of 1 V/s to generate current–voltage (*I–V*) curves (Figs. 1e and S11). More than 1000 *I*–*V* curves were recorded for each measurement, over 80% of which exhibited good overlap between the forward and reverse sweeps, indicating the stability of the molecular junction on the timescale of seconds. The slope of each *I–V* trace (i.e., conductance, G) was extracted and compiled into a conductance histogram for CYP1A1. As shown in Fig. 1g, three distinct peaks (denoted as Peaks I, II, and III) were observed at 0.22, 0.93, and 4.47 nS, which correspond to clustering patterns in the two-dimensional *I–V* map (Figs. 1f and S12). These peaks may represent three discrete charge-transport pathways through individual enzyme molecules^27^.

Our previous work demonstrated that reliable protein conductance measurements require at least one strongly coupled electrode-protein contact, formed either via covalent bonding or ligand-mediated interactions^22^. To evaluate the role of contact chemistry, the STM probe was passivated with mercaptoethanol (MCE) to suppress the formation of specific Au–S bonds. Under these conditions, Peaks II and III disappeared from the conductance histogram (Fig. S13), whereas Peak I (0.25 nS) persisted. This observation indicates that Peak I originates from asymmetric junction configurations involving one specific contact (e.g., Au–S bond at the substrate) and one nonspecific contact at the probe, a configuration previously observed in other protein junctions, such as antibodies and DNA polymerase^22,27^. In contrast, the higher conductance peaks (Peaks II and III) are consistent with junctions formed between two specific contacts, enabled by the presence of two surface-exposed cysteine residues on CYP1A1 (Cys20 and Cys502; Fig. S14). These chemical linkages promote strong electronic coupling between the enzyme and both electrodes, thereby facilitating efficient electron injection and transport across the junction. Our previous study further demonstrated that once strong chemical contacts are established, protein conductance exhibits relatively weak dependence on molecular length (*β* < 0.1 nm^−1^)^30^. Accordingly, although attachment at different cysteine residues may alter the effective transport length, such geometric variations may not necessarily lead to clearly resolvable conductance differences in the histogram, considering the intrinsic breadth of the conductance distributions (Fig. 1g). The pronounced conductance difference between Peaks II and III may be attributed to distinct charge-transport pathways with different intrinsic transport efficiencies.

CYP1A1 is a heme-containing enzyme, in which the ferric ion plays a critical role in maintaining the enzymatic activity (Fig. 1c). To evaluate the contribution of this metal center in charge transport through CYP1A1, the apo-form of the enzyme (apo-CYP1A1) was prepared by removing the ferric ion (see “Methods” and Fig. S15). Conductance measurement of apo-CYP1A1 revealed a bimodal distribution, with the highest conductance peak (Peak III) absent (Figs. 1h and S16). This result indicates that the ferric ion mediates a highly efficient charge transport pathway in the native holoenzyme. Similar phenomena have been reported in other metalloproteins. For instance, apo-azurin lacking its copper center exhibits a charge transport mechanism dominated by tunneling^20^, which differs from the wild-type azurin that displays an additional transport channel mediated by hopping through the metal ion^26,31^. It is also noteworthy that the bimodal conductance profile of apo-CYP1A1 closely resembles our previous observations for bivalently bound, non-redox-active proteins, such as antibodies^22^, implying that long-range charge transport can still occur through the amino acid matrix of CYP1A1, albeit less efficiently than in the presence of a redox-active center. Together, these results indicate that Peak III may correspond to charge transport through the metal active center, while Peak II is associated with tunneling via the peptide backbone and aromatic network within the protein matrix. Moreover, compared with holo-CYP1A1, the conductance of Peak II increases from 0.93 to 1.59 nS upon removal of the metal center (Fig. 1h). This enhancement likely reflects structural distortion or altered secondary-structure organization induced by heme extraction, as further evidenced by CD spectroscopy discussed below.

The catalytic activity of oxidoreductases typically depends on reduced coenzymes, such as NADPH and NADH, which serve as essential electron donors by providing the reducing equivalents required for molecular oxygen activation and substrate oxidation^32^. To investigate the effect of these coenzymes on protein conductance, 1 mM NADPH or NADH was added to the reaction buffer (Fig. S17). As shown in Figs. 1i and S18, the characteristic three-peak conductance distribution was retained in the presence of NADPH; however, the peak values increased significantly by factors of 2.0, 4.3, and 4.8 for Peaks I, II, and III, respectively. In contrast, NADH induced only modest enhancements of ~1.1-, 2.2-, and 2.2-fold for the corresponding peaks (Fig. S19). This pronounced disparity is consistent with the physiological preference of CYP1A1 for NADPH as its native coenzyme^33^, which enables more efficient electron transfer to the heme center. In agreement with this interpretation, enzymatic activity assays reveal a significantly higher catalytic efficiency of CYP1A1 in the presence of NADPH compared with NADH (Figs. S2 and S5). Collectively, these results reveal a strong correlation between protein conductance and enzymatic activity, suggesting that conductance measurements may serve as a sensitive probe for monitoring enzyme function.

The influence of enzymatic catalysis on protein conductance was evaluated using BaP as a model substrate for CYP1A1-mediated metabolism. As shown in Fig. 1j, the addition of 50 μM BaP results in a pronounced decrease in conductance relative to the CYP1A1-NADPH complex, with reductions to approximately 0.7-, 0.6-, and 0.6-fold for Peaks I, II, and III, respectively. For clarity, the conductance variations of CYP1A1 under different experimental conditions are summarized in Fig. 1k. The three conductance peaks exhibit distinct sensitivities to the enzymatic state of the protein. Peak I shows minimal variation across all conditions, consistent with previous reports indicating that conductance in this asymmetric junction configuration is dominated by contact resistance^22^. In contrast, Peak III displays the most pronounced response, with its conductance increasing approximately 5-fold upon NADPH addition but decreasing by ~60% when both NADPH and BaP are present. Peak II follows a similar trend, although with a smaller magnitude of change. Previous studies have demonstrated that ligand-induced conformational changes can substantially change protein conductance, as observed for streptavidin^22^. Given that enzymatic catalysis involves a sequence of coordinated steps accompanied by conformational rearrangements, it is reasonable to infer that coenzyme binding and BaP turnover may induce structural changes in CYP1A1 that, in turn, modulate charge-transport efficiency. Notably, these effects are directionally distinct: coenzyme binding enhances conductance, whereas substrate metabolism reduces it, with the metal-mediated pathway (Peak III) being more susceptible than the protein-matrix pathway (Peak II).

### Effect of conformational changes on CYP1A1 conductance

**Fig. 2:**
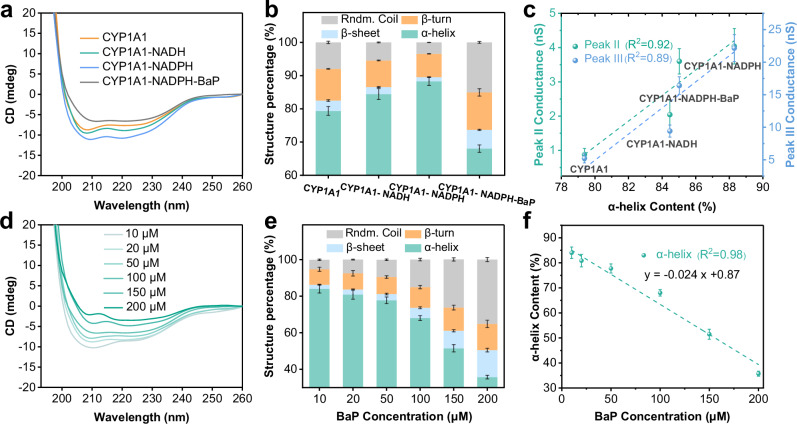
Conformational change of CYP1A1 under different catalytic conditions. **a**, **b** CD spectra and corresponding secondary-structure contents of CYP1A1, CYP1A1-NADH, CYP1A1-NADPH, and CYP1A1-NADPH-BaP. **c** Correlation between α-helical content and measured conductance. **d**, **e** CD spectra and secondary-structure analysis of CYP1A1-NADPH at different BaP concentrations. **f** Dependence of α-helical content on BaP concentration. Data are presented as mean ± s.d. (*n* = 3). Source data are provided as a Source data file.

The conformational changes of CYP1A1 under different enzymatic conditions were characterized by CD spectroscopy. As shown in Fig. 2a, holo-CYP1A1 displays two pronounced negative bands at 222 and 208 nm, characteristic of a predominantly α-helical secondary structure. This spectral signature is consistent with the crystal structure of CYP1A1, in which α-helices constitute the core scaffold surrounding the heme prosthetic group (Fig. S1). Removal of the heme center resulted in a significant attenuation of the negative band at ~208 nm, whereas the band at ~222 nm remained largely preserved. This selective spectral change indicates that heme extraction disrupts local α-helical packing and tertiary interactions in the vicinity of the prosthetic group (Fig. S20). The influence of coenzymes on protein conformation was further evaluated by introducing 1 mM NADPH or NADH into the buffer. Both coenzymes induced more negative ellipticity at α-helix-characteristic wavelengths, indicative of conformational rearrangements upon coenzyme binding. Quantitative secondary-structure analysis reveals that the α-helical content increases by ~9% in the presence of NADPH (Fig. 2b), suggesting conformational stabilization of CYP1A1 and a transition toward a more ordered, catalytically competent state^34^. In contrast, the addition of BaP (100 μM) led to a substantial decrease in α-helical content of ~20%, likely reflecting partial helix unwinding or rearrangement of the protein structures to accommodate the bulky substrate during catalysis^34,35^.

To bridge the gap between electrical signals and protein structural features, a correlation analysis was performed by plotting peak conductance values against the α-helical content of the enzyme (Fig. 2c). A strong positive linear correlation was observed for both Peak II and Peak III (*R*^2^ ≈ 0.90). In contrast, other secondary-structure components, including β-sheets, β-turns, and random coils, exhibited inverse correlations with conductance (Fig. S21 and Table S1). This quantitative relationship indicates that electron-transport efficiency through CYP1A1 is closely coupled to the degree of α-helical order and packing density within the protein scaffold. Such behavior is consistent with crystallographic studies of CYP1A1, which reveal that the active-site pocket is predominantly defined by the F and I helices^34^. These helices contain dense networks of aromatic residues (e.g., Tyr, Trp, and Phe), which are known to facilitate charge transport^36,37^. Thus, increased structural disorder is expected to disrupt aromatic stacking and increase the effective tunneling barrier. Notably, a similar dependence of conductance on α-helical content has been reported for bovine serum albumin, where ligand binding reduced α-helical content and led to a corresponding decrease in conductance^38^. Collectively, these results demonstrate that protein conductance can potentially serve as a sensitive reporter of secondary-structure integrity and its dynamic remodeling during enzymatic catalysis.

The dependence of CYP1A1 conformation on substrate concentration was further examined. As shown in Fig. 2d, e, increasing concentrations of BaP lead to a gradual decrease in α-helical content, accompanied by compensatory increases in other secondary structural elements. Correlation analysis reveals a strong inverse relationship between α-helical content and BaP concentration (*R*^2^ = 0.98; Fig. 2f). This behavior is consistent with an induced-fit mechanism, in which the F and I helices that frame the active-site pocket undergo localized remodeling to accommodate substrate binding^34^. This process is accompanied by increased conformational flexibility, enhanced exposure of the active-site topology, and formation of a more planar binding platform. In the CD spectra, these conformational adaptations manifest as a progressive attenuation of the negative ellipticity at 222 nm (Fig. 2d). Since the enzyme conductance is strongly correlated with α-helical content, the BaP-induced loss of α-helicity is expected to result in a corresponding decrease in CYP1A1 conductance with increasing substrate concentration, as discussed later in Fig. 4. Collectively, these substrate-concentration-dependent changes in protein conformation and conductance establish electrical measurements as a sensitive approach to study the catalytic kinetics of enzymes during substrate turnover.

### Effect of heme redox state on CYP1A1 conductance

**Fig. 3:**
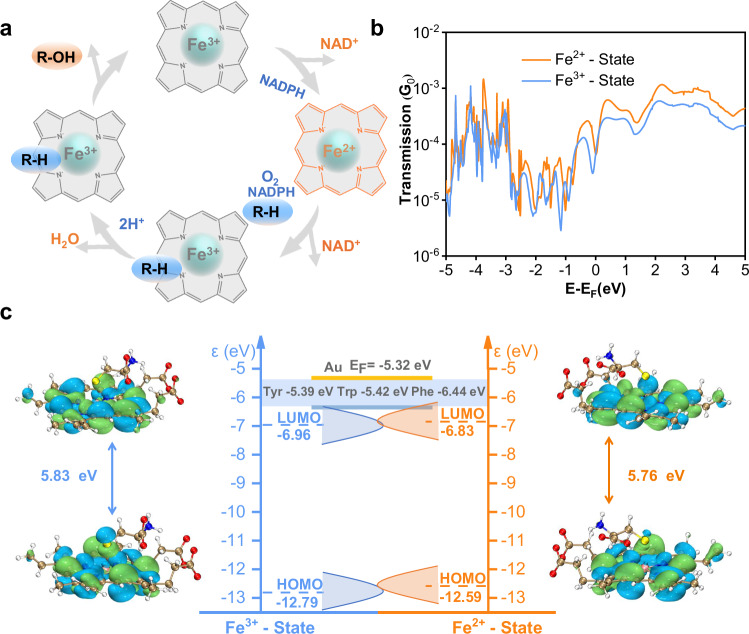
Effect of heme redox state on CYP1A1 conductance. **a** Schematic illustration of the catalytic cycle of CYP1A1 heme iron during substrate metabolism. R-H denotes the substrate, and R-OH denotes the hydroxylated product. **b** The transmission function of the CYP1A1 active center in the Fe^2+^ and Fe^3+^ states. **c** HOMO-LUMO energy levels of the heme iron in ferric (Fe^3+^) and ferrous (Fe^2+^) states, calculated within a protein-solvent environment, relative to the Fermi level of Au (*E*_F_ = −5.32 eV). Source data are provided as a Source data file.

In conductance measurement of CYP1A1, the metal center acts as an efficient stepping stone for charge transport, giving rise to the high‑conductance state (Peak III; Fig. 1g, h). In a canonical CYP1A1 catalytic cycle, NADPH binding not only increases the α-helical content but also drives reduction of the heme iron from the ferric (Fe^3+^) to the ferrous (Fe^2+^) state, thereby enabling molecular oxygen binding and initiating substrate oxidation (Fig. 3a)^39^. This redox transition is expected to modify the electronic structure of the heme center and its coupling to surrounding residues and cofactors, ultimately influencing charge transport efficiency. To verify this hypothesis, density functional theory (DFT) calculations were performed to analyze the electronic structure of the heme center in both oxidation states. A hybrid quantum mechanics/molecular mechanics (QM/MM) framework with electrostatic embedding was employed to account for protein environmental effects and solvent contributions (see “Methods”). The model comprised the porphyrin macrocycle and the axial cysteine ligand coordinated to the iron center. As shown in Fig. 3c, in the resting ferric state, the lowest unoccupied molecular orbital (LUMO) is primarily localized on the porphyrin ring at −6.96 eV, whereas the highest occupied molecular orbital (HOMO) is distributed over the porphyrin and cysteine ligand at −12.79 eV. Reduction of the iron center to the ferrous state results in higher orbital delocalization, with the LUMO and HOMO shifting upward to −6.83 and −12.59 eV, respectively. Consequently, the HOMO-LUMO gap narrows from 5.83 to 5.76 eV. When considering a protein junction within gold electrodes, the elevated LUMO level in the ferrous state brings it closer to the Fermi level of the electrode (−5.32 eV)^40^, as well as the redox energy levels of nearby aromatic residues (Tyr, −5.39 eV; Trp, −5.42 eV; and Phe, −6.44 eV) located within ~2 nm of the active center (Fig. S22)^41^. This improved energetic alignment, combined with the reduced HOMO-LUMO gap, implies that the reduced state of CYP1A1 is inherently more conductive^26^.

To quantitatively evaluate the effect of heme redox state on charge-transport through CYP1A1, quantum transport calculations were performed using the non-equilibrium Green’s function formalism combined with DFT (NEGF-DFT). Due to the high computational cost, the transport calculations were carried out on the active-site cluster with the geometries and orientations extracted from fully optimized QM/MM structures. As shown in Fig. 3b, the Fe^2+^ state exhibits a significantly higher transmission coefficient near the Fermi energy than the Fe^3+^ state, providing theoretical support for the enhanced conductance observed experimentally in the reduced enzyme. This result may account for the conductance decrease in the presence of BaP, where the heme iron is oxidized to the ferric state, thereby increasing the energy barrier for electron transport. However, it should be noted that both coenzyme binding and substrate turnover induce significant conformational changes that also modulate protein conductance (Fig. 2). Thus, isolating the individual contribution of the heme redox state from that of conformational effects remains challenging. These factors likely act synergistically to regulate electronic coupling and charge-transport efficiency.

### Substrate concentration dependence of conductance change in CYP1A1

**Fig. 4:**
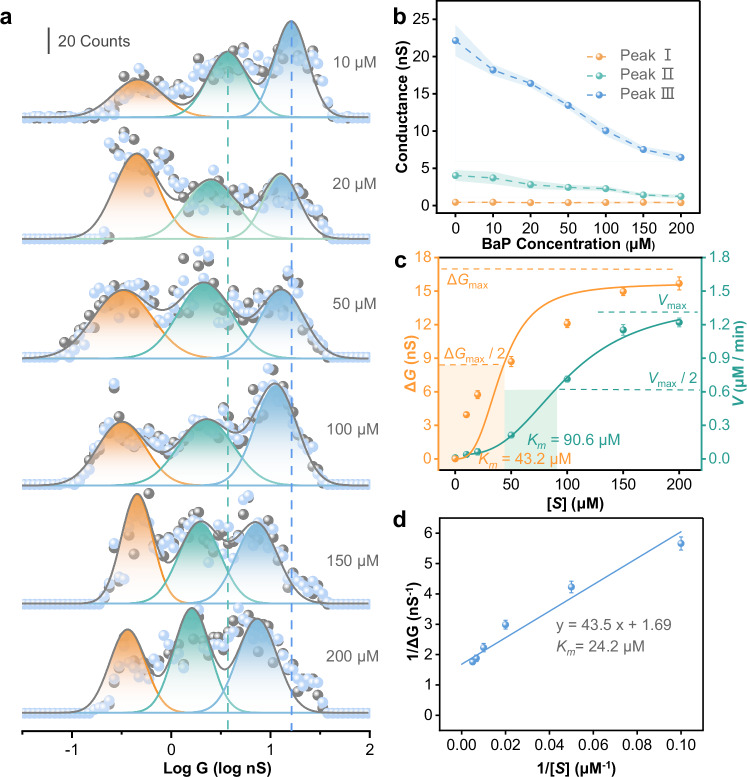
Enzymatic reaction kinetics based on single-molecule CYP1A1 conductance. **a** Stacked conductance distribution of CYP1A1 measured at varying concentrations of BaP. **b** Dependence of conductance peak values on BaP concentration. **c** Michaelis–Menten plots derived from conductance changes of CYP1A1 (orange curve), and enzymatic reaction rates measured by GC-MS (green curve). **d** Lineweaver–Burk double-reciprocal plot constructed from conductance-based data, showing the inverse relationship between Δ*G* and substrate concentration. Data are presented as mean ± s.d., *n* = 3. Source data are provided as a Source data file.

The dependence of CYP1A1 conductance on substrate concentration was investigated by varying BaP concentration from 10 to 200 μM (Figs. 4a and S23). The stacked plot of the conductance distributions clearly reveals a shift in peak position toward lower conductance, though this trend appears visually less distinct due to the logarithmic scale of the x-axis. When the peak values are plotted as a function of BaP concentration, a negative correlation is observed for both Peaks II and III, with the conductance approaching a plateau at concentrations beyond 200 μM (Fig. 4b). This substrate-dependent conductance change is consistent with the variation in α-helical content extracted from CD measurements (Fig. 2f), yielding linear correlations for both Peaks II and III (Figs. S24 and S25). Moreover, as discussed above, BaP binding promotes oxidation of the heme iron, thereby reducing the efficiency of charge transport. Since the conductance histogram represents an ensemble-averaged distribution derived from thousands of individual single-molecule measurements, the observed concentration dependence indicates that increasing BaP concentration shifts a larger fraction of CYP1A1 molecules into the enzyme-substrate complex. This progressive population shift leads to a lower average conductance until saturation is reached at high substrate concentrations.

In enzymatic catalysis, the reaction rate depends on substrate concentration and is commonly described by the Michaelis–Menten equation:

1 $$v=\frac{{V}_{\max }\times [S]}{{K}_{m}+[S]}$$

where *v* is the reaction rate, [*S*] is the concentration of substrate, *K*_*m*_ is the Michaelis constant, and *V*_max_ is the maximum reaction rate. The underlying mechanism involves the binding of the enzyme (*E*) to the substrate (*S*) to form an enzyme-substrate complex (*ES*), which is then converted into products (*P*):

2 $$E+S\rightleftharpoons {ES}\to E+P$$

The rate of product formation is thus proportional to the concentration of the *ES* complex and can be expressed as:

3 $$v={K}_{{{\rm{cat}}}}\times \left[{ES}\right]$$

where *K*_cat_ is the catalytic rate constant (turnover number, in s^−1^), and [*ES*] is the concentration of the enzyme-substrate complex. If we define Δ*G* as the change in conductance of CYP1A1 at different BaP concentrations, it can serve as a direct indicator of the population of enzyme molecules in the *ES* state (i.e., with the active center occupied). Accordingly, the Michaelis–Menten relationship can be approximated by:

4 $$v={K}_{{{\rm{cat}}}}\times \alpha \times \Delta G=\frac{{V}_{\max }\times [S]}{{K}_{m}+[S]}$$

Where *α* is a conversion coefficient relating conductance to *ES* concentration (μM nS^−1^). Based on Eq. 4, a kinetic model for enzymatic catalysis can be constructed, predicting a sigmoidal profile when plotting Δ*G* as a function of BaP concentration (Fig. 4c, orange line). From this fit, the apparent Michaelis constant (${K}_{m}^{{{\rm{app}}}}$), defined as the substrate concentration at which the reaction rate reaches half of *V*_max_, was estimated to be approximately 43.2 μM. For comparison, the classic Lineweaver–Burk plot was applied to the same data set, yielding a ${K}_{m}^{{{\rm{app}}}}$ of 24.2 μM (Fig. 4d). The discrepancy between the two calculation methods likely arises because the double-reciprocal transformation distorts the experimental error structure, particularly at low substrate concentrations, making it inherently less accurate than nonlinear regression^42^.

To further validate the model, gas chromatography-mass spectrometry (GC-MS) was used to independently determine the reaction rate under identical conditions, yielding a *K*_*m*_ of 90.6 μM (Fig. 4c, green line). This value is about twofold higher than that obtained from the conductance measurements, likely reflecting differences in the underlying observables and model assumptions. In the GC-MS assay, *K*_*m*_ is derived from the rate of BaP consumption, whereas in the conductance measurements, it is inferred from changes in enzyme conductance that reflect the population of the enzyme-substrate complex. Although these quantities are related, they probe distinct aspects of the catalytic cycle and may therefore yield systematically different kinetic parameters.

Compared with previous literature values reporting *K*_*m*_ for CYP1A1-catalyzed BaP metabolism (19.0–25.6 μM)^43,44^, the 43.2 μM obtained from conductance measurements is significantly higher. This deviation likely reflects the heterogeneous nature of the conductance assay, in which the enzyme is immobilized on a gold surface. Under these conditions, the observed reaction rate is governed by both intrinsic catalytic kinetics and the mass transport of substrate to a spatially confined active site. To quantify the influence of diffusion, we calculated the Damköhler number (Da) and obtained a value of approximately 0.23 (see “Methods”). This places the system in the mixed kinetic-diffusion regime (0.1 < Da < 10), rather than in the limits of purely kinetic-controlled (Da ≪ 0.1) or purely diffusion-controlled (Da ≫ 10) behavior. In this regime, the experimentally determined ${K}_{m}^{{{\rm{app}}}}$ is expected to be larger than the intrinsic kinetic constant by a factor of approximately (1 + Da) ≈ 1.23. These results collectively demonstrate that the conductance response of CYP1A1 provides a sensitive and quantitative measure of enzymatic kinetics. This establishes a robust framework for employing protein-based bioelectronic devices to monitor enzymatic reactions, offering significant advantages in terms of speed and cost compared with conventional analytical methods.

### Monitoring catalytic dynamics based on protein conductance

**Fig. 5:**
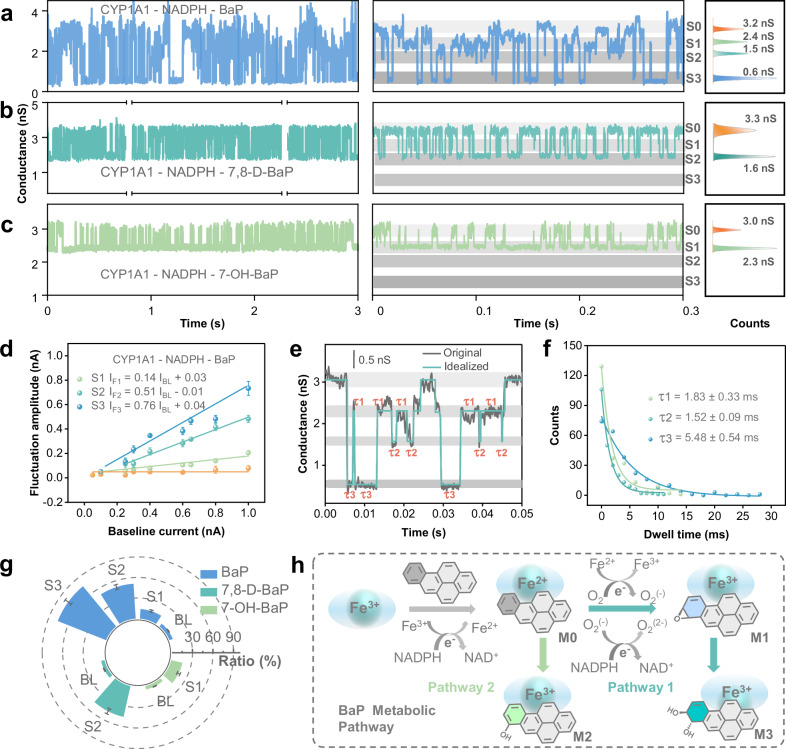
Monitoring catalytic dynamics of CYP1A1 based on conductance measurement. **a**–**c** Representative 3-s *i–t* traces during CYP1A1-mediated metabolism of BaP, 7,8-D-BaP, and 7-OH-BaP, respectively. The full traces (left), zoomed-in 0.3-s segments (middle), and corresponding current histograms (right) are shown. **d** Dependence of TN amplitude (*I*_F_) on the baseline current (*I*_BL_). TN amplitude of S1, S2, and S3 exhibits an approximately linear correlation with the baseline current, with characteristic slopes for each state. Data are presented as mean ± s.d. The error bars represent the standard deviation from the statistical analysis of TN events extracted from multiple measurements (*n* ≥ 3). **e** Example *i*–*t* trace segment idealized using a segmental k-means algorithm with hidden Markov modeling, fitted using QUB software (green trace). **f** Dwell time distributions for conductance states S1, S2, and S3 obtained from QUB fits. Lifetimes were extracted via monoexponential fitting. **g** Comparison of TN amplitude proportionality relative to baseline current for BaP and its metabolic intermediates. BL denotes baseline current fluctuation. Data are presented as mean ± s.d. (*n* ≥ 3). **h** Proposed BaP metabolic pathways based on conductance measurements combined with HPLC-MS/MS analysis. Pathway 1 corresponds to metabolic activation; pathway 2 corresponds to detoxification. The CYP1A1 heme active site is schematically represented by a blue ellipse. Source data are provided as a Source data file.

Capturing intermediate steps in enzymatic catalysis is essential for elucidating the molecular mechanism underlying enzyme function. However, this remains challenging because transient catalytic events occur on microsecond-to-millisecond timescales, exceeding the temporal resolution of most conventional techniques. Given that protein conductance is sensitive to conformational changes, it offers a promising approach for monitoring catalytic intermediate states in real time. To explore this possibility, we performed high-resolution current–time (*i–t*) measurements on single-molecule CYP1A1 junctions at a sampling rate of 50 kHz, enabling the detection of events as brief as 20 μs. A molecular fishing strategy was employed to capture individual CYP1A1 molecules^27^, during which *i–t* traces were recorded for 90 s at a constant bias of −200 mV (see “Methods”). Based on the reported catalytic rate constant of CYP1A1 (~2.0 s^−1^)^43^, this acquisition window is sufficient to capture multiple complete catalytic cycles. Among the collected *i–t* traces, approximately 40% exhibited large current fluctuations superimposed on the baseline (referred to as telegraph noise, TN), a characteristic signal previously associated with enzymatic activity, such as in DNA polymerase systems^27^. A representative TN trace is shown in Figs. 5a and S26, S27. Notably, TN signals appeared only in the presence of both NADPH and BaP. Control experiments conducted under non-enzymatic conditions showed no significant TN activity, confirming the catalysis-dependent origin of the observed fluctuations (Fig. S28).

Approximately 300 *i–t* traces with valid TN signals were collected and analyzed. Most current fluctuations were negative-going, indicating transitions into lower conductance states. Detailed analysis revealed four distinct conductance states, denoted S0 through S3 in descending order (middle and right panels in Fig. 5a). The S0 state, corresponding to the baseline conductance, is likely associated with the initial enzyme-substrate complex. The lower conductance states (S1–S3) are hypothesized to represent successive conformational intermediates of CYP1A1 during the catalytic cycle. To elucidate the transition pathways among these states, we performed a statistical analysis of state-to-state transitions using more than 1500 events collected from ~50 measurements. The resulting transition-density heatmap (Fig. S29) reveals a clear kinetic pattern: transitions originate predominantly from the baseline state S0 and bifurcate into either S1 or S3. Transitions between S2 and S3, as well as between S1 and S3, are also prominent, whereas transitions, such as S0 → S2 and S1 → S2 occur only rarely. This transition pattern likely reflects distinct catalytic steps during BaP turnover.

Interestingly, the absolute amplitude of TN signals was strongly dependent on the baseline current, consistent with our prior observations^27^. To quantify this relationship, we selected eight TN segments with relatively stable baselines and extracted the fluctuation amplitudes for each conductance state. The mean amplitudes were then plotted against their corresponding baseline currents (Fig. 5d), revealing an approximately linear correlation as indicated by the fitted lines. Based on the slopes of these fits, the fluctuation amplitudes for the S1, S2, and S3 states correspond to 14%, 51%, and 76% of the baseline current, respectively. These relative amplitudes provide characteristic signatures for distinguishing among the different conductance states. In contrast, the fluctuation amplitude in the S0 state remained minimal and was also observed in control measurements lacking BaP, suggesting that TN fluctuations on the baseline current are not related to enzymatic activity.

To elucidate the mechanistic basis underlying transitions between conductance states, we employed high-resolution mass spectrometry (Q-exactive Orbitrap) to identify intermediates formed during CYP1A1-catalyzed BaP metabolism. Under complete catalytic conditions, two primary metabolites were detected, including 7-hydroxybenzo[a]pyrene (7-OH-BaP) and benzo[a]pyrene-7,8-dihydrodiol (7,8-D-BaP) (Fig. S30), consistent with the known metabolic pathways of BaP^45^. These two intermediates were selected as representative species to examine their individual contributions to conductance transitions. As intermediates can be recognized by CYP1A1 and engage in subsequent catalytic transformations, they allow a focused investigation of distinct reaction steps^46^. Upon addition of 7,8-D-BaP, TN signals featuring a two-state transition were observed (Fig. 5b). To characterize the associated conductance states, we plotted the fluctuation amplitude against the baseline current, which yielded a relative amplitude of 46% (Fig. S31a). This value closely matches the S0 → S2 transition observed during BaP metabolism (Fig. 5d, g), suggesting that S2 may correspond to the catalytic processing of 7,8-D-BaP. Similarly, the introduction of 7-OH-BaP generated a distinct two-state TN signal with an amplitude-to-baseline ratio of ~17% (Figs. 5c and S31b), consistent with the S0 → S1 transition. Accordingly, the S1 state can be assigned to a conformational intermediate of CYP1A1 with 7-OH-BaP bound.

Using these two intermediates, we successfully identified the S1 and S2 conductance states based on the relative amplitude characteristics of the TN signals. However, the S3 state could not be directly assigned due to the absence of a stable, isolatable intermediate. Based on the established BaP metabolic sequence, (+)-BaP-7,8-epoxide (7,8-E-BaP), the precursor of 7,8-D-BaP^45^, is the most likely candidate for the S3 state. However, 7,8-E-BaP is unstable, prone to hydrolysis, and therefore unsuitable for direct state assignment. Nevertheless, it is reasonable to associate S3 with the epoxidation step of BaP catalysis. Aligning these results with the canonical CYP1A1-mediated BaP metabolic pathway, we propose the following correspondence between conductance states and catalytic intermediates: S0 (initial enzyme-substrate complex) → S3 (7,8-E-BaP) → S2 (7,8-D-BaP) and S0 → S1 (7-OH-BaP). These two distinct metabolism pathways are consistent with the observed transition patterns among the conductance states (Fig. S29). For example, S0 frequently transitions to both S3 and S1. The reversions from S3 to S0 reflect the premature release or spontaneous hydrolysis of 7,8-E-BaP^47^. Additionally, infrequent direct transitions from S1 to S3 were detected, which may result from competitive substrate binding, wherein 7,8-E-BaP rebinds to the enzyme, displacing 7-OH-BaP. These findings collectively demonstrate that real-time tracking of single-molecule conductance during enzymatic catalysis can resolve transient intermediate states with high temporal resolution.

To gain further insights into the transient kinetics of the intermediate steps, hidden Markov modeling and kinetic simulations were performed using the Quantify Unknown Biophysics (QUB) software (qub.mandelics.com) on the *i–t* trajectories recorded during the metabolism of 50 μM BaP by CYP1A1^48^. The multilevel traces were first idealized using a piecewise k-means segmentation approach (Figs. 5e and S32). This approach enabled the extraction of the dwell times (*τ*_D_) for each conductance state, reflecting the lifetimes of enzyme-bound intermediates and their subsequent catalytic transformations. As shown in Fig. 5f, the dwell time distributions for states S1, S2, and S3 follow monoexponential decay, yielding average lifetimes of *τ*₁ = 1.83 ± 0.33 ms (S1), *τ*₂ = 1.52 ± 0.09 ms (S2), and *τ*₃ = 5.48 ± 0.54 ms (S3), respectively. The relatively long lifetime of S3 is consistent with previous theoretical studies suggesting that the electrophilic epoxidation step, leading to the formation of 7,8-E-BaP, is the rate-determining step in the canonical BaP activation pathway^49^. This correlation provides indirect support for assigning S3 to the epoxidation step. According to the established metabolic sequence of CYP1A1-catalyzed BaP oxidation^50^, 7,8-D-BaP undergoes a second epoxidation at the C10 position to yield benzo[a]pyrene diol epoxide (BPDE). The shorter lifetime of S2 compared to S3 (*τ*₂ ≈ 1/3 *τ*₃) suggests that this second epoxidation occurs more rapidly, potentially due to a reduced activation energy barrier^49^. On the other hand, 7-OH-BaP is a representative product of the detoxification pathway and does not undergo further activation. The *τ*₁ value observed following the addition of 7-OH-BaP may reflect the lifetime of a stabilized enzyme-substrate complex that is not further catalytically transformed. Based on the analysis of these conductance fluctuations, a putative catalytic sequence for CYP1A1-mediated BaP metabolism can be proposed (Fig. 5h). The enzyme-substrate complex (M0) follows two competing pathways: in the dominant pathway, M0 undergoes initial epoxidation to form 7,8-E-BaP (M1), which is subsequently hydrolyzed by epoxide hydrolase to 7,8-D-BaP (M3), and then further epoxidized by CYP1A1 to generate BPDE. In the alternative detoxification route, M0 is converted into phenolic derivatives, such as 7-OH-BaP (M2), which are not subject to further catalytic processing. These findings demonstrate that single-molecule conductance measurements, when performed with sufficient temporal resolution, allow direct observation of enzymatic kinetics and the resolution of transient catalytic intermediates. Nonetheless, limitations in junction stability and bandwidth may restrict the detection of faster, short-lived events. Future work leveraging solid-state bioelectronic platforms may overcome these constraints and further advance the real-time electronic interrogation of enzyme catalysis^24^.

## Discussion

In summary, we demonstrate that enzymatic catalytic dynamics can be directly monitored in real time through electrical conductance measurements of individual enzyme molecules. By incorporating CYP1A1 into a fixed-gap STM junction, we achieved single-molecule sensitivity to conductance fluctuations associated with enzymatic activity. Periodic conformational fluctuations during catalysis modulate junction conductance, either through structural rearrangement of the protein and/or changes in the oxidation state of the heme cofactor, thereby establishing a direct correlation between enzymatic function and electrical response. Our results demonstrate that conductance changes as a function of substrate enable the extraction of apparent kinetic parameters that align well with bulk assays. Notably, the high temporal resolution of electrical detection allows the identification of transient catalytic steps and the reconstruction of a stepwise reaction pathway. In the case of CYP1A1-catalyzed BaP metabolism, four distinct conductance states were resolved and correlated with specific catalytic intermediates, thus providing single-molecule insight into competing activation and detoxification pathways.

Despite these advancements, several limitations of the present study should be acknowledged. The contributions of cofactor redox state, protein conformational changes, and electrode-protein contact geometry to the measured conductance are intrinsically coupled, complicating the isolation of individual factors under experimental conditions. Moreover, although theoretical calculations were extended to account for protein environmental effects and solvent contributions, they remain necessarily simplified owing to the computational complexity of electronic structure calculations for large biomolecular systems. Nonetheless, this study establishes a successful proof of concept for protein-based bioelectronic platforms capable of resolving transient enzymatic kinetics with high temporal resolution, offering a promising approach for future investigations in mechanistic enzymology, bioelectronics, and drug discovery.

## Methods

### Enzyme activity assessment

Enzymatic activity was evaluated by quantifying cofactor consumption (NADH or NADPH) via UV–vis spectroscopy, along with fluorescence-based detection of the reaction product, resorufin, using 7-ER as the substrate. Assays were initiated by separately adding 1 mM NADH or NADPH to reaction mixtures containing 1 µM CYP1A1, 1 µM CPR, and 1 mM 7-ER in 50 mM PBS (pH 7.4). Absorbance at 340 nm, characteristic of NADPH, was monitored at 30-min intervals to determine cofactor depletion (Fig. S2). Parallel control experiments confirmed that NADPH consumption was solely attributable to enzymatic activity, with negligible contributions from thermal degradation or auto-oxidation (Fig. S3). Formation of resorufin was further validated by measuring fluorescence emission at 590 nm (Fig. S5). Combined, these analyses revealed a CYP1A1-specific activity of 1.9 U mg^−1^, demonstrating its strict dependence on NADPH as a cofactor.

### Preparation of CYP1A1-modified substrate

CYP1A1-modified gold substrates were prepared through the formation of a self-assembled monolayer (SAM) of 3-mercaptopropionic acid (MPA) on a gold surface, followed by EDC/NHS-mediated activation of terminal carboxyl groups to enable covalent immobilization of CYP1A1 via amide bond formation. The procedure was as follows: (1) gold substrates (1.2 × 1.2 cm) were flame-annealed in hydrogen to improve surface crystallinity and cleanliness. (2) SAMs were formed by immersing the substrates in 1 mM MPA in ethanol for 1 h. (3) After rinsing with ethanol and drying under nitrogen, the carboxyl groups were activated in PBS buffer (pH 7.4) containing 0.4 mM EDC and 0.1 mM NHS for 3 h. (4) The activated surfaces were incubated overnight at 4 °C with 1 µM CYP1A1 in PBS (pH 7.4), enabling amide bond formation between the enzyme and activated carboxyl groups. (5) Unreacted NHS esters were quenched using 1 mM ethanolamine for 30 min to block nonspecific binding sites. A schematic illustration of the complete immobilization protocol is provided in Fig. S6.

### Protein conductance measurement

STM probes were fabricated by electrochemical etching of 0.25 mm diameter gold wire. To prevent current leakage, the etched tip is insulated with a high-density polyethylene coating^51^. Optical micrographs illustrating the tip morphology after etching and insulation are shown in Fig. S10. Gold substrates were prepared by electron-beam evaporation of a 10 nm chromium adhesion layer followed by a 200 nm gold film onto silicon wafers, using a Lesker PVD 75 system.

Protein conductance measurements were conducted using a PicoSPM scanning probe microscope (Agilent Technologies), with data acquisition managed by a PCI-6289 DAQ card (National Instruments). The STM probe was initially engaged at a set-point current of 4 pA under a −0.2 V bias and allowed to stabilize for 2 h prior to measurement^22^. Subsequently, *I*–*V* sweep and *i–t* measurements were performed. For each *I*–*V* measurement, over 1000 individual curves were collected, and those exhibiting overlapping forward and reverse sweeps were selected to construct conductance histograms. *I–t* traces were recorded with the bias voltage held constant at −0.2 V during the probe-hold phase.

All conductance measurements were repeated at least three times to ensure reproducibility. Measurements were performed in a reaction buffer consisting of 50 mM PBS (pH 7.4), supplemented with 1 mM NADPH or NADH, 1 µM CPR, and 1 µM epoxide hydrolase (EH; included only in BaP metabolism experiments).

### Preparation of Apo-CYP1A1

To investigate the contribution of the heme Fe^3+^ to electron transport, CYP1A1 was converted to its apo-form by acid denaturation to disrupt the porphyrin structure and release the iron, followed by EDTA chelation and filtration to obtain apo-CYP1A1^52,53^. Specifically, a 100 µM solution of CYP1A1 was acidified to pH 2.0 using HCl and incubated at 2–4 °C for 20 min to induce heme dissociation. The released Fe^3+^ was subsequently chelated with excess EDTA (5 mM) at pH 2.5 and 25 °C. The resulting EDTA-Fe^3+^ complexes and unbound EDTA were removed by ultrafiltration using a 30 kDa molecular weight cutoff (MWCO) membrane, followed by multiple washes with PBS. Successful removal of ~97% of Fe^3+^ from CYP1A1 was confirmed by UV–vis spectroscopic analysis of the 10-fold diluted ultrafiltrate, compared against reference spectra of Fe^3+^, EDTA, and EDTA-Fe^3+^ (Fig. S15). Functional assessment of the resulting apo-CYP1A1 showed no detectable NADPH consumption over a 3 h period, confirming the complete loss of enzymatic activity.

### CD spectroscopy and estimation of secondary structural contents

CD spectroscopy was performed at 25 °C using a Chirascan spectropolarimeter (Applied Photophysics Chirascan, UK). The CYP1A1 and other solutions were analyzed in a well-cleaned quartz cuvette with a path length of 0.1 cm at wavelengths in the far-ultraviolet region (190–260 nm). To analyze the CD spectra and estimate the secondary structure content, we employed the CDNN software or the SELCON3 program (available on the Dichroweb server; Birkbeck College, University of London, UK) combined with singular value decomposition (SVD) for data processing^54^.

### Computational chemistry methods

ORCA 6.0.1^55^ was used for all HOMO-LUMO calculations in this study. We systematically screened the spin multiplicities for the iron oxidation states (Table S2), followed by geometry optimization using the r2SCAN-3c composite method, which was selected to achieve a good balance between computational efficiency and geometric accuracy. To accurately resolve the molecular orbital distributions under physiological conditions, energy calculations were performed using the ωB97M-V functional in conjunction with the def2-TZVP basis within a QM/MM electrostatic embedding scheme^56^. The protein environment was modeled using a rigorous point-charge field generated via PDB2PQR server^57^ using the Amber force field, which accounts for pH-dependent protonation states (pH 7.0) and the specific electrostatic potential of the protein matrix, embedded within a conductor-like polarizable continuum model (CPCM) for implicit solvation. Wavefunction analyses were carried out using Multiwfn version 3.8 (dev)^58^, and molecular structures were visualized using VMD version 1.9 (Fig. S33 and Supplementary Data 1).

CP2K 2025.1^59^ was used for all transmission spectrum calculations in this study. Geometry optimizations and electronic structure calculations were performed using the Quickstep within the mixed Gaussian and plane waves (GPW) approach. The PBE exchange-correlation functional was employed in combination with Grimme’s D3 dispersion correction with Becke–Johnson damping (DFT-D3(BJ)). Core electrons were described by GTH pseudopotentials, while valence electrons were treated using DZVP-MOLOPT-SR-GTH basis sets for C, H, O, N, S, and Fe atoms, and SZV-MOLOPT-SR-GTH basis sets for Au atoms, with a plane-wave cutoff of 500 Ry. To ensure convergence in this metallic system, spin-polarized calculations (UKS) were performed using Fermi–Dirac smearing at an electronic temperature of 300 K. Quantum transport properties were simulated using the non-equilibrium Green’s function (NEGF) formalism combined with DFT (via the CP2K + SMEAGOL interface/module)^60^ (Fig. S34 and Supplementary Data 2).

### Mass spectrometric analysis of BaP metabolites

BaP metabolic consumption was quantified using gas chromatography-mass spectrometry (GC-MS; Agilent), while metabolite identification was performed via high-resolution liquid chromatography coupled with quadrupole Orbitrap mass spectrometry (LC-Q-Orbitrap MS; Q-exactive, Thermo Scientific). The reaction mixture consisted of 50 mM PBS (pH 7.4), 1 mM NADPH, 50 μM BaP (dissolved in 5 μL DMSO), 1 μM CYP1A1, 1 μM CPR, and 1 μM EH. The final DMSO concentration was maintained below 5.0%, a level previously shown not to impair enzymatic activity^45,50^. Reaction mixtures were incubated at 37 °C for 3 h. BaP metabolites were extracted twice with 1 mL ethyl acetate, and the combined organic layers were evaporated to dryness under reduced pressure. The resulting residue was reconstituted in 1 mL of methanol for subsequent MS analysis (Fig. S30).

### Characterization of surface functionalization

The successful fabrication of CYP1A1-functionalized chips was validated through comprehensive physicochemical characterization. Elemental composition was analyzed using X-ray photoelectron spectroscopy (XPS; PHI 5000 VersaProbe III) equipped with a monochromatic Al Kα source (hν = 1486.6 eV). Charge correction was applied using the C 1*s* peak at 284.8 eV as a reference. Protein immobilization was confirmed by identifying characteristic amide I and amide II vibrational bands via FTIR (Bruker Vertex 70 v) with a diamond attenuated total reflectance (ATR) accessory. The thickness of the CYP1A1 layer was measured by spectroscopic ellipsometry (TF-UVISEL).

### Calculation of the Damköhler number

The relative importance of reaction versus diffusion is commonly assessed by the Damköhler number (Da) (Eq. 5), which quantifies the ratio of the maximum reaction rate to the mass-transport rate^61^:

5 $${{\rm{Da}}}=\frac{{V}_{\max }}{{K}_{{{\rm{L}}}}{K}_{m}}=\frac{{K}_{{{\rm{cat}}}}\Gamma }{(D/{{\rm{\delta }}}){K}_{m}}$$

Where *K*_cat_ is the turnover number (≈2.0 s^−1^)^43^, *Γ* is the surface coverage of the immobilized enzyme, *D* is the diffusion coefficient of the substrate (≈3.3 × 10^−6^ cm^2^ s^−1^)^62^, δ is the diffusion layer thickness (assumed to be ≈200 μm), *K*_*m*_ is the intrinsic Michaelis constant, ${K}_{{{\rm{L}}}}$ is the mass transfer coefficient.

Assuming a closely packed monolayer, the surface coverage can be estimated from the molecular footprint of CYP1A1 (19.5 × 23.5 nm^2^, i.e., *A*_enz_ ≈ 458.25 nm^2^) as Eq. 6

6 $$\Gamma=\frac{1}{{N}_{{{\rm{A}}}}{A}_{{{\rm{enz}}}}}\approx 3.62\times {10}^{-13}\,{{\rm{mol}}}\,{{{\rm{cm}}}}^{-2}$$

Using an intrinsic *K*_*m*_ of 19 μM^43^, the resulting Da is approximately 0.23. This places our system in the mixed kinetic-diffusion control regime (0.1 < Da < 10), rather than in the regime of complete diffusion control (Da ≫ 10).

### Reporting summary

Further information on research design is available in the Nature Portfolio Reporting Summary linked to this article.

## Supplementary information

Supplementary Information

Description of Additional Supplementary Files

Supplementary Data1

Supplementary Data2

Reporting Summary

Transparent Peer Review File

## Source data

Source Data

Source Data

## Data Availability

The experimental datasets generated and analyzed during the current study, including single-molecule conductance measurements, CD spectroscopy, and enzymatic activity assays, are provided in the Source data file. The atomic coordinates of the optimized computational models from the electronic structure calculations (QM/MM and NEGF-DFT) are provided as separate plain, unformatted text files in Supplementary Data 1 and 2. All other relevant data supporting the findings of this study are available within the paper and its Supplementary Information, and from the corresponding author upon request. Source data are provided with this paper.
