# Supplementary material for: Real-time electrical monitoring of enzymatic catalytic dynamics at the single-molecule level
Source: Nat Commun. 2026 Jun 5;17:7198. doi: 10.1038/s41467-026-74020-0 (PMC13396217; doi:10.1038/s41467-026-74020-0)
Supplement: Supplementary file 1 — Supplementary Information [file 41467_2026_74020_MOESM1_ESM.pdf]

Supporting Information

Real-Time Electrical Monitoring of Enzymatic Catalytic Dynamics at the Single-Molecule Level

Zhimin Fan[1], Zusen Chen[1], Zhengwen Gong[2], Sanjun Shi[1], Mingdi Xu[1], Xiaonan Feng[1], Yulu Liu[1], Ya Hu[1], Xiaoduo Chen[1], Guomao Zheng[1], and Bintian Zhang[1,*]

[1] Shenzhen Key Laboratory of Precision Measurement and Early Warning Technology for Urban Environmental Health Risks, School of Environmental Science and Engineering, Southern University of Science and Technology, Shenzhen 518055, China

[2] Key Laboratory of Systems Health Science of Zhejiang Province, School of Life Science, Hangzhou Institute for Advanced Study, University of Chinese Academy of Sciences, Hangzhou 310024, China

* Corresponding author, E-mail: zhangbintian@sustech.edu.cn (Dr. Zhang)

Table of Contents

Materials and reagents ……………………………………………………………..3

Supplementary Figures 1-34…………………………………………………… 3-23

Supplementary Tables 1-2………………………………………………………24

Materials and reagents

Recombinant Human Cytochrome P450 1A1 (CYP1A1) and benzo[a]pyrene (BaP) were obtained from Sigma-Aldrich. 7-Hydroxybenzo[a]pyrene and BaP-7,8-dihydrodiol were obtained from J&K Scientific. Ethylenediaminetetraacetic acid (EDTA), 50 mM phosphate-buffered saline (PBS, pH 7.4), 1-(3-dimethylaminopropyl)-3-ethylcarbodiimide (EDC), and N-hydroxysuccinimide (NHS) were purchased from Macklin. Gold wire (99.99%, 0.25 mm diameter) was purchased from Beijing Jiaming Platinum Co. All chemicals were of reagent grade or higher and were used as received without further purification. The reaction buffer consisted of 50 mM PBS (pH 7.4) supplemented with 1 μM cytochrome P450 reductase (CPR). Ultrapure water, purified using a Milli-Q system (>18.2 MΩ·cm), was used in all experiments.

Supplementary Figures

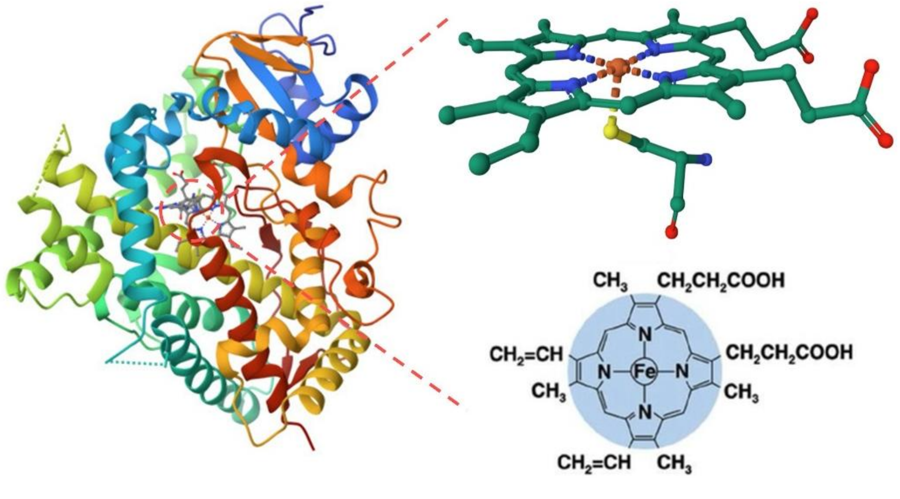

Fig. S1. Structural schematic of CYP1A1 (left) and catalytic heme center (right). The heme iron is coordinated by a porphyrin ring with cysteine thiolate as the proximal axial ligand.

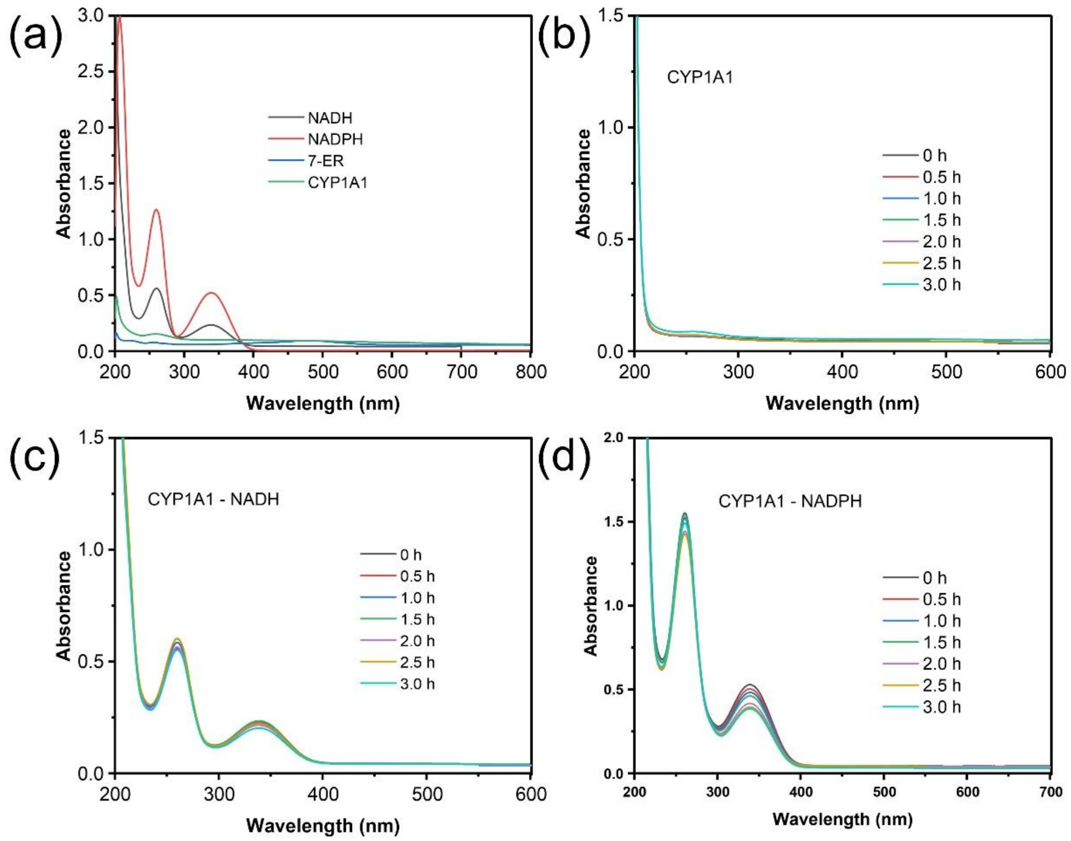

Fig. S2. Enzyme activity assays under varying conditions. **(a)** UV-Vis absorption spectra of individual components: CYP1A1 (1 μM) , 7-ethoxyresorufin (7-ER, 1 mM), NADH (1 mM), and NADPH (1 mM) in reaction buffer at 25°C. Time-dependent UV-Vis absorption spectra of **(b)** CYP1A1, **(c)** CYP1A1-NADH, and **(d)** CYP1A1-NADPH, in the presence of 1 mM 7-ER. Spectra were recorded at 30-min intervals. Significant decrease in NADPH absorption at 340 nm indicates progressive cofactor depletion, contrasting with that of the CYP1A1-NADH system, where the NADH consumption is negligible.

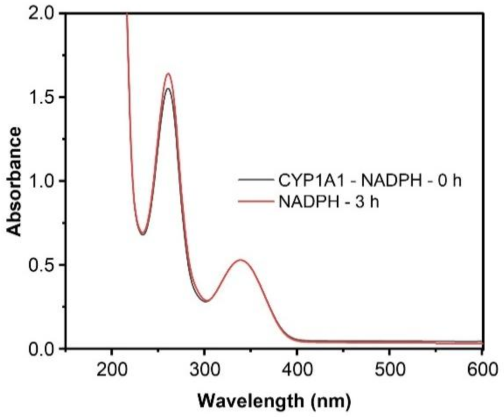

Fig. S3. Control Experiment for Validating Enzymatic NADPH consumption. NADPH alone and CYP1A1-NADPH complex in reaction buffer at 25°C. Absorbance at 340 nm remained stable over 3 h in NADPH systems. This confirms the spectral decrease observed in activity assays exclusively results from CYP1A1-mediated NADPH oxidation, ruling out significant contributions from thermal degradation or dissolved oxygen.

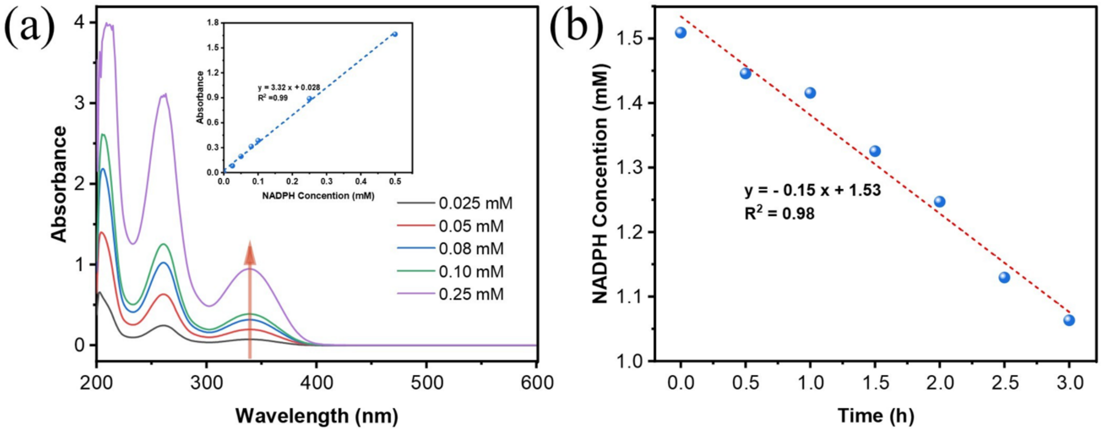

Fig. S4. Quantification of NADPH consumption and enzyme-activity assessment. (a) UV-Vis spectra of NADPH (0.025-0.25 mM) in reaction buffer at 25°C. Inset: Linear correlation (R^2 > 0.99) between NADPH concentration and absorbance at 340 nm. **(b)** Enzymatic NADPH consumption kinetics quantified by ΔA_{340} nm decrease over 3 h. CYP1A1 specific activity was calculated as 1.9 U/mg from NADPH oxidation rates.

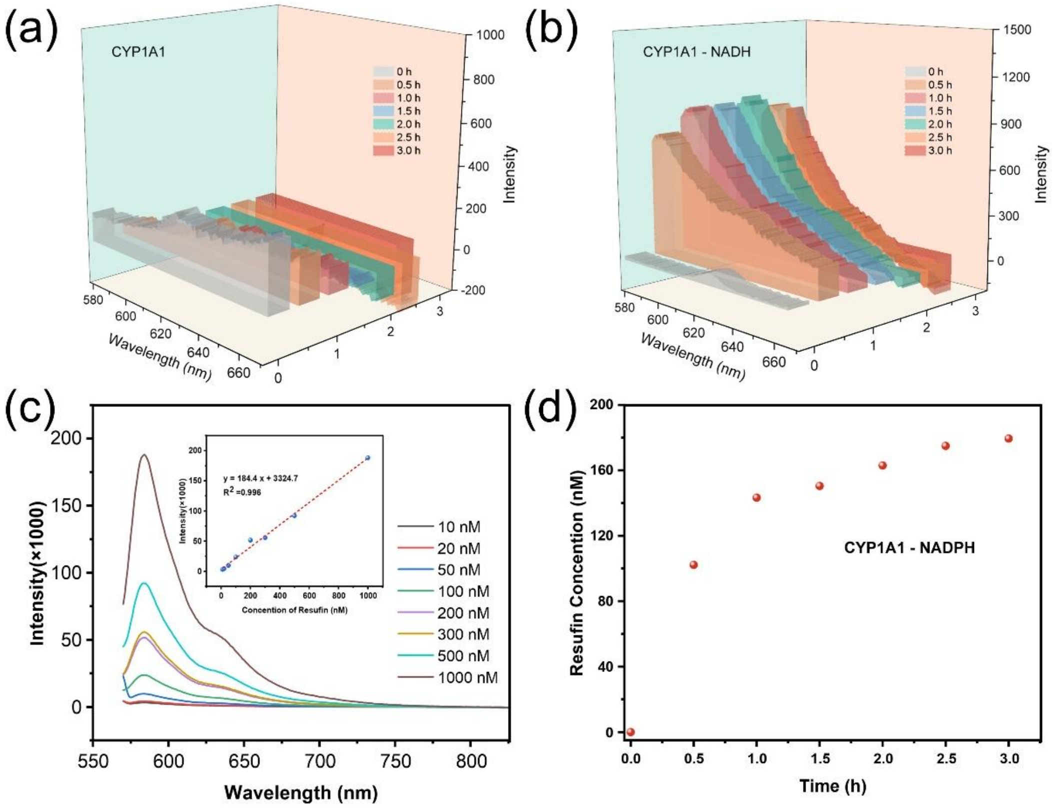

Fig. S5. Resorufin fluorescence spectra for enzyme-activity assessment. Fluorescence spectra of **(a)** CYP1A1 (1 μM) reacts with 7-ER (1 mM), **(b)** CYP1A1-NADH reacts with 7-ER (1 mM), **(c)** Resorufin standard curve (10-1000 nM); inset shows linear calibration ($R^2 > 0.99$). **(d)** Time-dependent resorufin formation in CYP1A1-NADPH system. Reactions were performed in reaction buffer at 25°C. 7-ER conversion yielded ~200 nM resorufin. Fluorescence measurements were performed with an excitation wavelength of 570 nm and an emission wavelength of 590 nm.

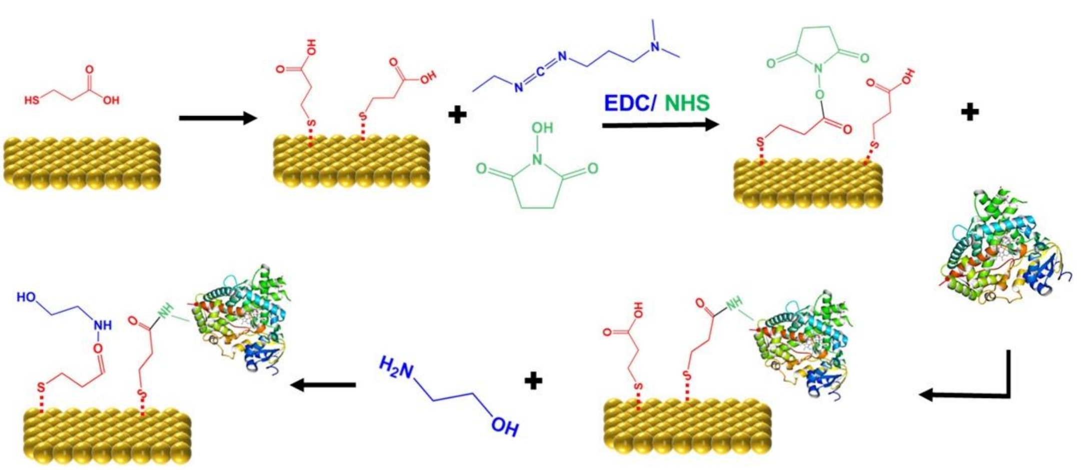

Fig. S6. Surface functionalization scheme for CYP1A1 immobilization.

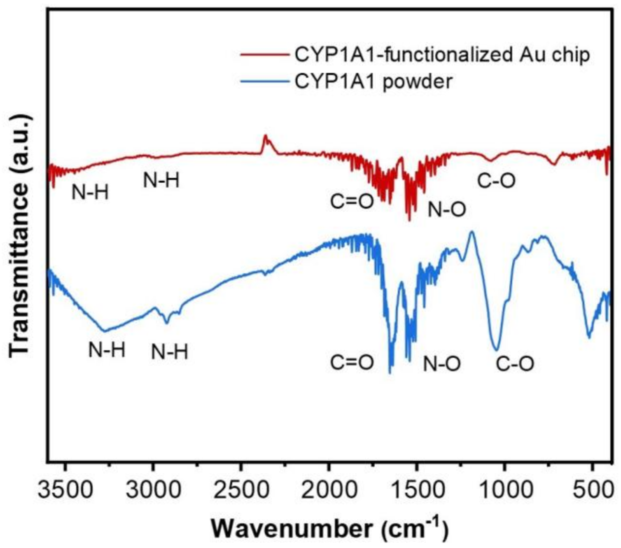

Fig. S7. FTIR spectroscopic validation of CYP1A1 immobilization. CYP1A1-functionalized Au chip (red), CYP1A1 powder (blue). Characteristic amide I (1640-1670 cm^{-1}) and amide II (1540-1570 cm^{-1}) bands in CYP1A1-functionalized chip and powder demonstrate spectral congruence, confirming successful enzyme immobilization via surface conjugation.

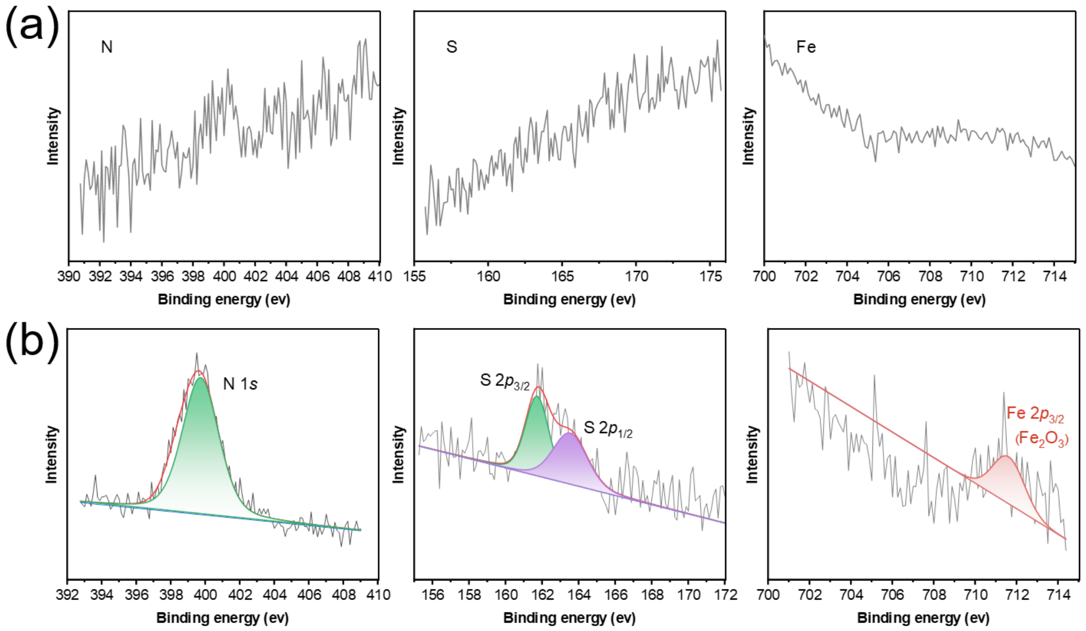

Fig. S8. XPS spectra of the CYP1A1-modified chip. (a) XPS spectra of a bare Au chip. **(b)** XPS spectra of the CYP1A1-modified chip. The presence and detection of characteristic elements N (N 1*s* peak at 399.5 - 400.5 eV), S (S 2*p* peak at 162 - 164.5 eV), and Fe (Fe 2*p* peak at 710.5 - 711.5 eV) on the CYP1A1-modified chip surface confirm successful surface modification.

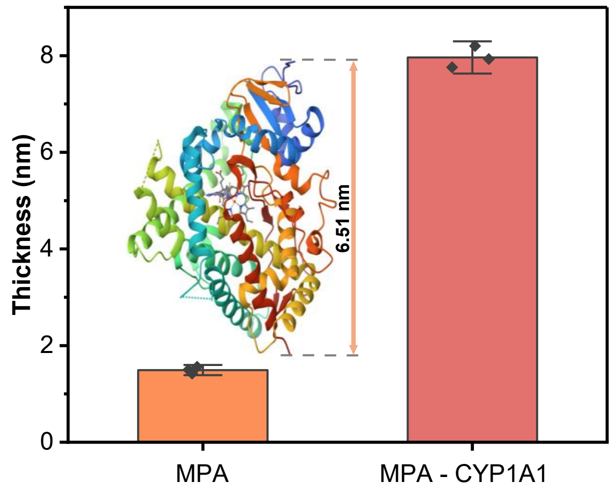

Fig. S9. Ellipsometry analysis of the CYP1A1-modified chip. The thickness of the self-assembled monolayer (SAM) of MPA on the Au surface is 1.50 nm. Following protein immobilization, the thickness of the MPA-CYP1A1 layer is 7.93 nm. The resulting increase in thickness of 6.43 nm corresponds to the thickness of the

immobilized CYP1A1 protein layer. This value is consistent with the crystallographic dimensions of CYP1A1 (6.51 nm). (Data are presented as mean ± s.d., n = 3)

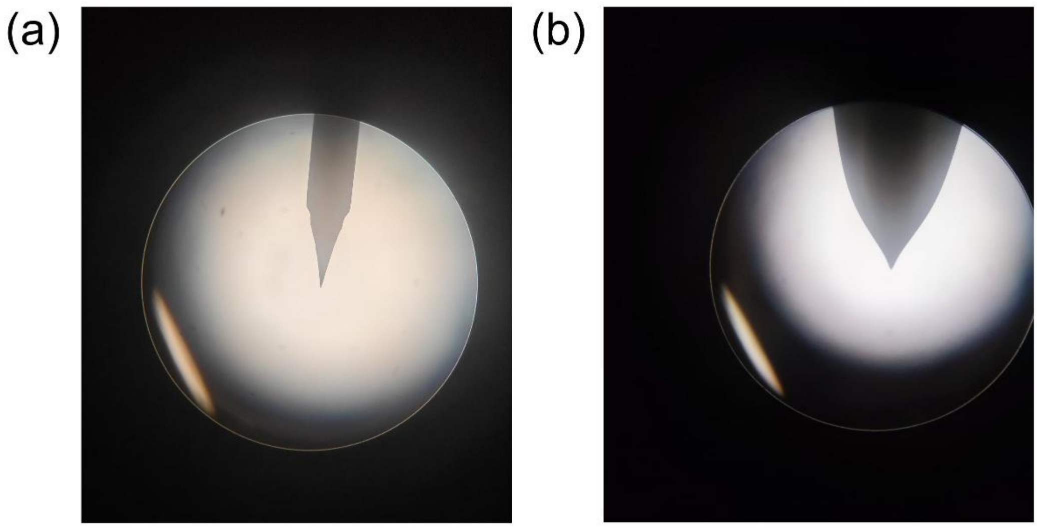

Fig. S10. Optical images of the STM probe. (a) STM probe after electrochemical etching. **(b)** Tip coated with high density polyethylene (HDPE).

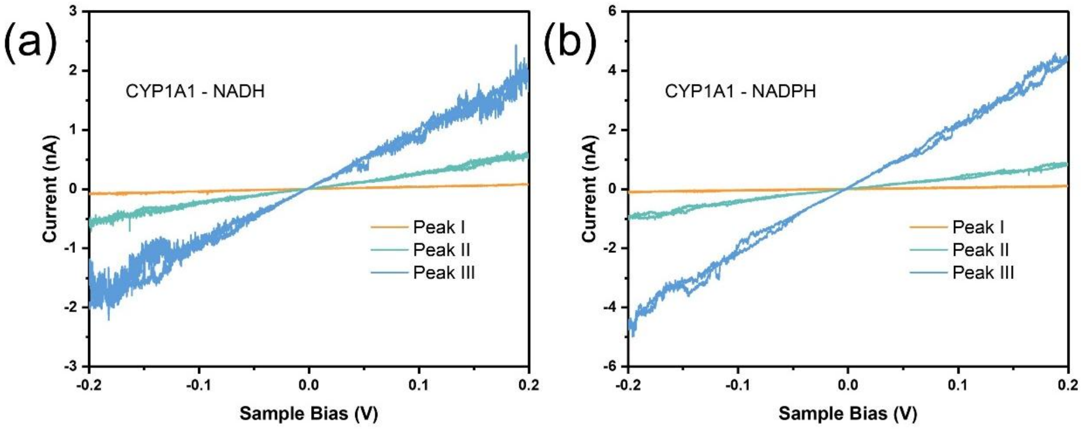

Fig. S11. Representative I-V curves. (a) CYP1A1-NADH. **(b)** CYP1A1-NADPH. In both systems, the forward (up-sweep) and reverse (down-sweep) voltage scan curves overlap. The yellow, green, and blue I-V traces correspond to representative traces for Peaks I, II, and III, respectively.

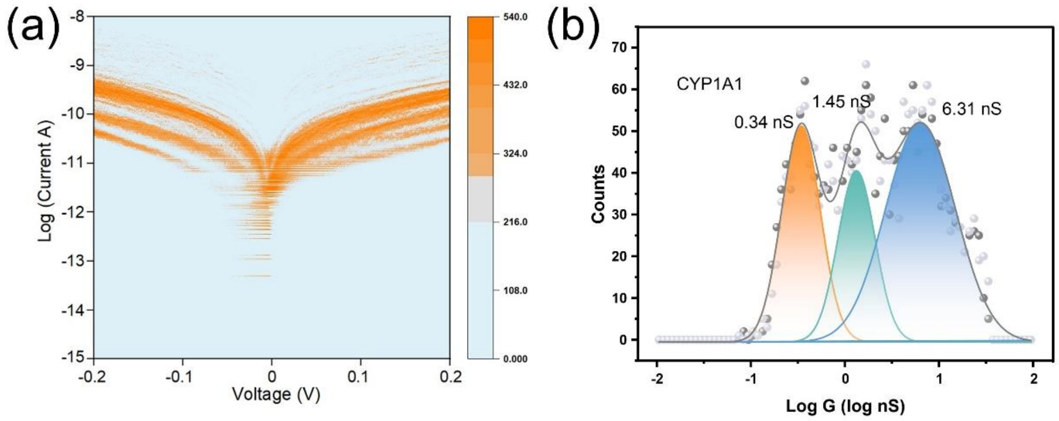

Fig. S12. Conductance distributions of CYP1A1. (a) Two-dimensional current-voltage map of CYP1A1 obtained by log-transforming ~1,000 I-V curves. **(b)** Conductance distribution histogram of CYP1A1, derived from the slopes of the I-V traces, shows a three-peak distribution.

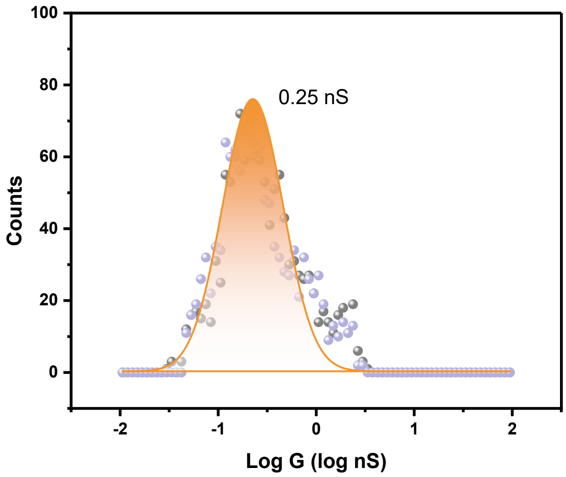

Fig. S13. Conductance distribution for protein conductance measurements using MCE-passivated gold tips. Protein conductance showed only Peak I (Peaks II and III absent), with triplicate values of 0.23, 0.25, and 0.26 nS.

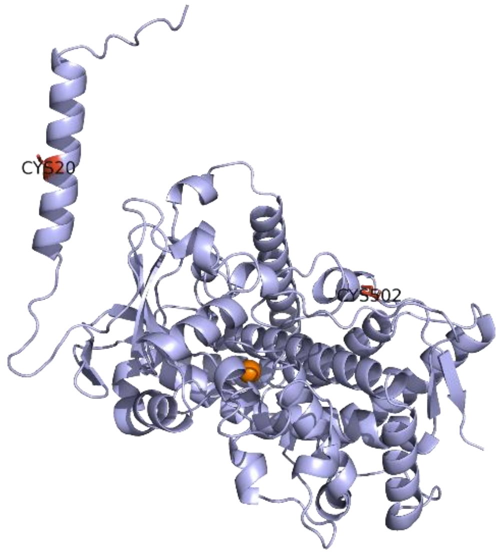

Fig. S14. Surface-exposed cysteines in CYP1A1. Protein backbone, purple; active-site Fe^{3+}, orange; surface-exposed cysteines Cys20 and Cys502, red. These cysteines may mediate specific contacts.

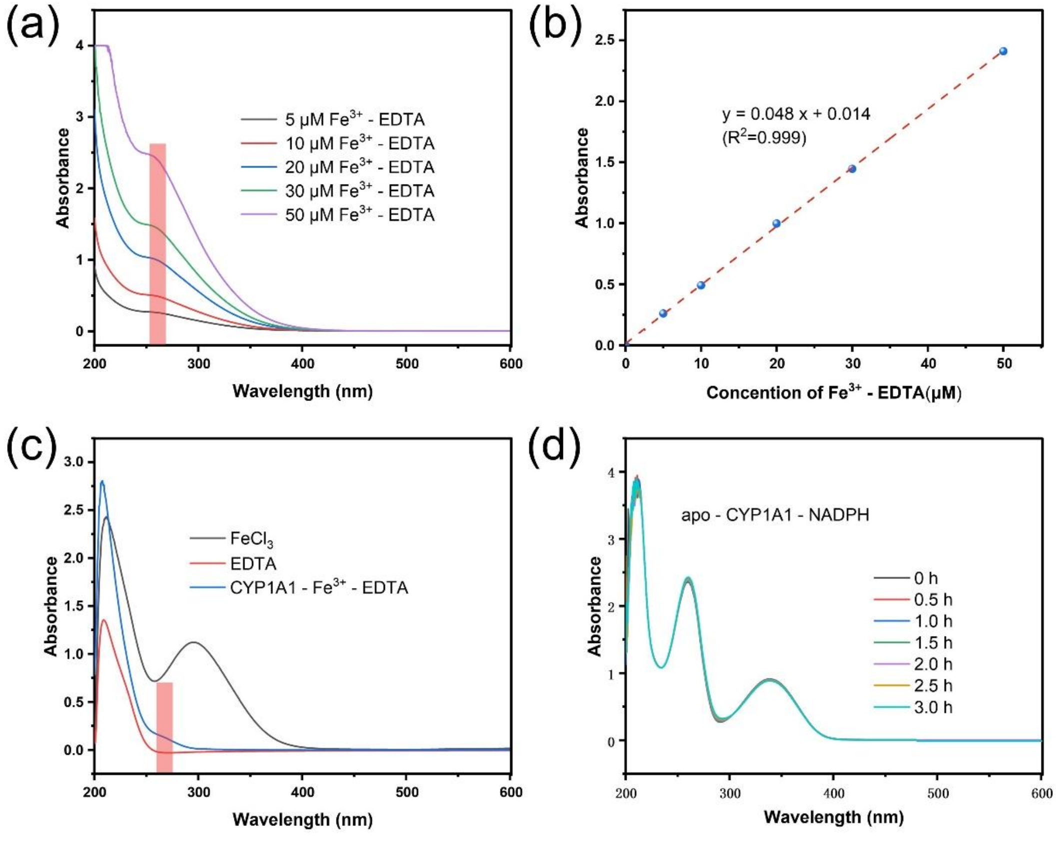

Fig. S15. Validation of Fe^{3+} removal from CYP1A1 via EDTA chelation. (a) UV-vis

spectra of Fe^{3+}-EDTA complexes (5-50 μM), showing a characteristic absorption peak at 265 nm. **(b)** Linear calibration curve (R^2 > 0.99) for Fe^{3+}-EDTA quantification. **(c)** Spectral comparison of aqueous Fe^{3+} (absorption ~300 nm), EDTA, and Fe^{3+}-EDTA formed by chelating Fe^{3+} from CYP1A1. The characteristic absorption peak of Fe^{3+} shifted from ~300 nm to 265 nm, indicating Fe^{3+}-EDTA complex formation. Quantification based on the standard curve indicated that approximately 97% of the Fe^{3+} was chelated from CYP1A1. **(d)** Enzymatic activity assessment shows no detectable NADPH oxidation with apo-CYP1A1 (ΔA_{340} < 0.02 over 3 h), demonstrating complete loss of enzymatic activity.

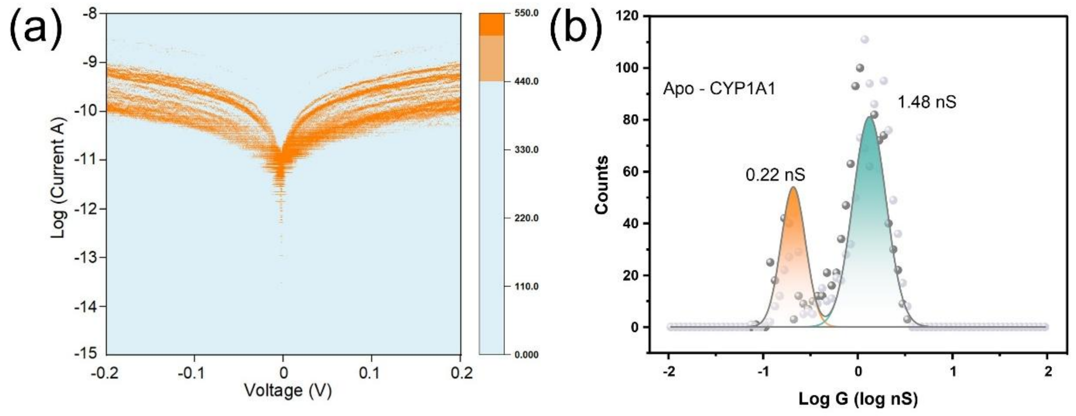

Fig. S16. Conductance distributions of apo-CYP1A1. (a) Two-dimensional current-voltage map of apo-CYP1A1 obtained by log-transforming ~1,000 I-V traces. **(b)** Conductance distribution histogram, derived from the slopes of the I-V traces, shows a bimodal distribution. These results indicate the disappearance of conductance Peak III, resulting in a bimodal distribution characterized solely by Peaks I and II. This observation suggests that the Fe^{3+} likely plays a role in the electron transport pathway associated with the high-conductance state (Peak III).

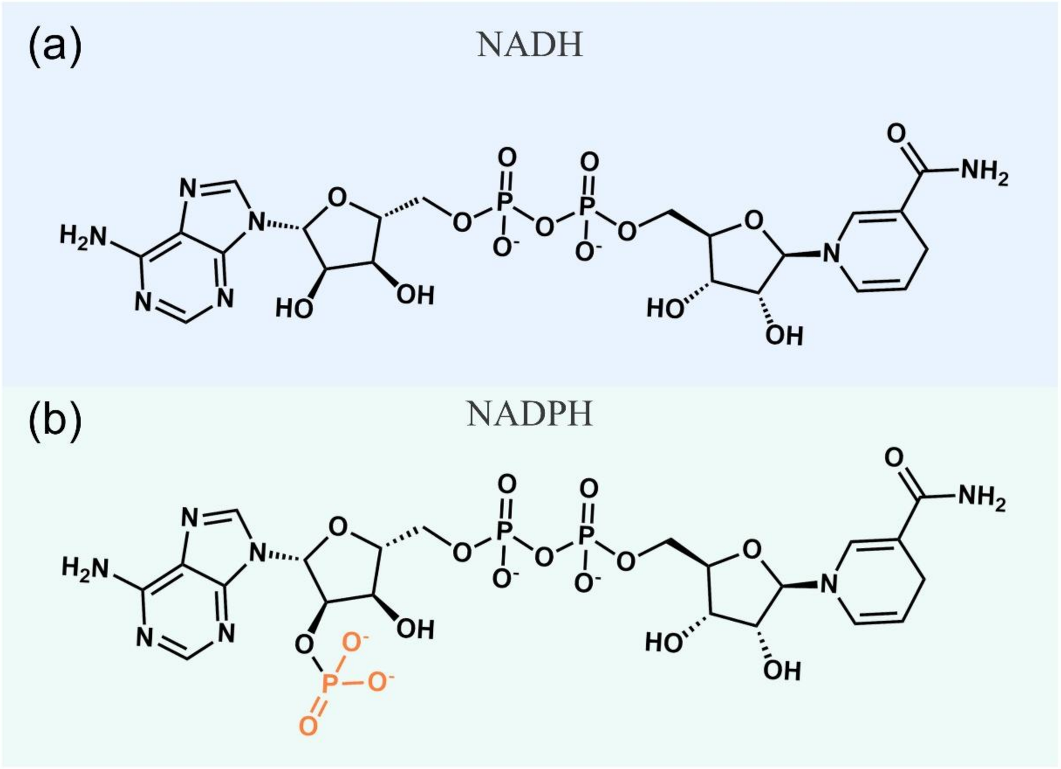

Fig. S17. Chemical structures of (a) NADH and (b) NADPH. NADPH bears an additional 2′-phosphate group on the adenosine ribose moiety relative to NADH, imparting enhanced binding affinity and enzymatic selectivity.

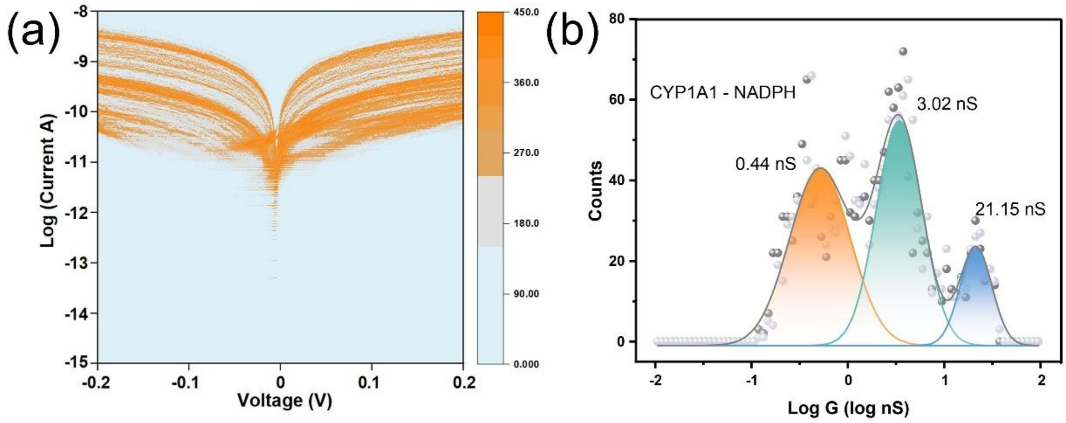

Fig. S18. Conductance distributions of CYP1A1-NADPH. (a) Two-dimensional current-voltage map of CYP1A1-NADPH obtained by log-transforming ~1,000 I-V traces. **(b)** Conductance distribution histogram, derived from the slopes of the I-V traces, shows a three-peak distribution.

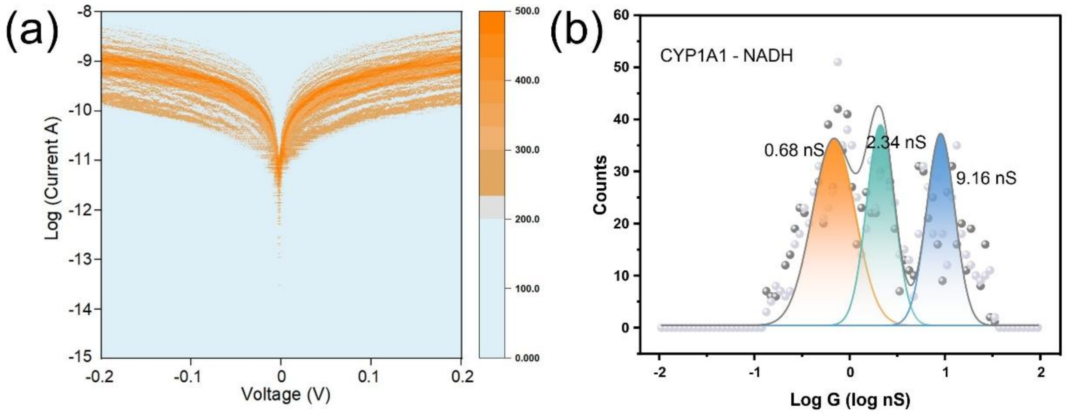

Fig. S19. Conductance distributions of CYP1A1-NADH. (a) Two-dimensional current-voltage map of CYP1A1-NADH obtained by log-transforming ~1,000 I-V traces. **(b)** Conductance distribution histogram, derived from the slopes of the I-V traces, shows a three-peak distribution.

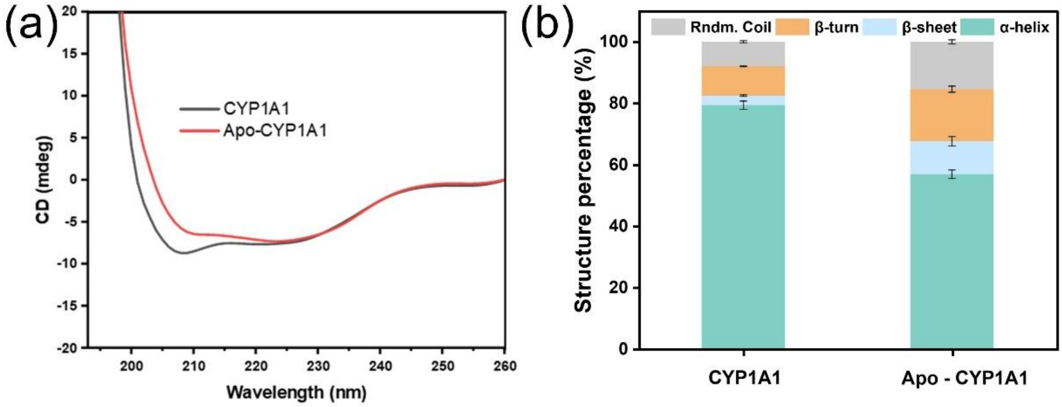

Fig.S20 Comparison of CD spectra and secondary structures between CYP1A1 and apo-CYP1A1. (a) CD spectra of CYP1A1 and apo-CYP1A1. **(b)** Changes in secondary structure content of CYP1A1 and apo-CYP1A1. Data point is presented as mean ± s.d., n = 3.

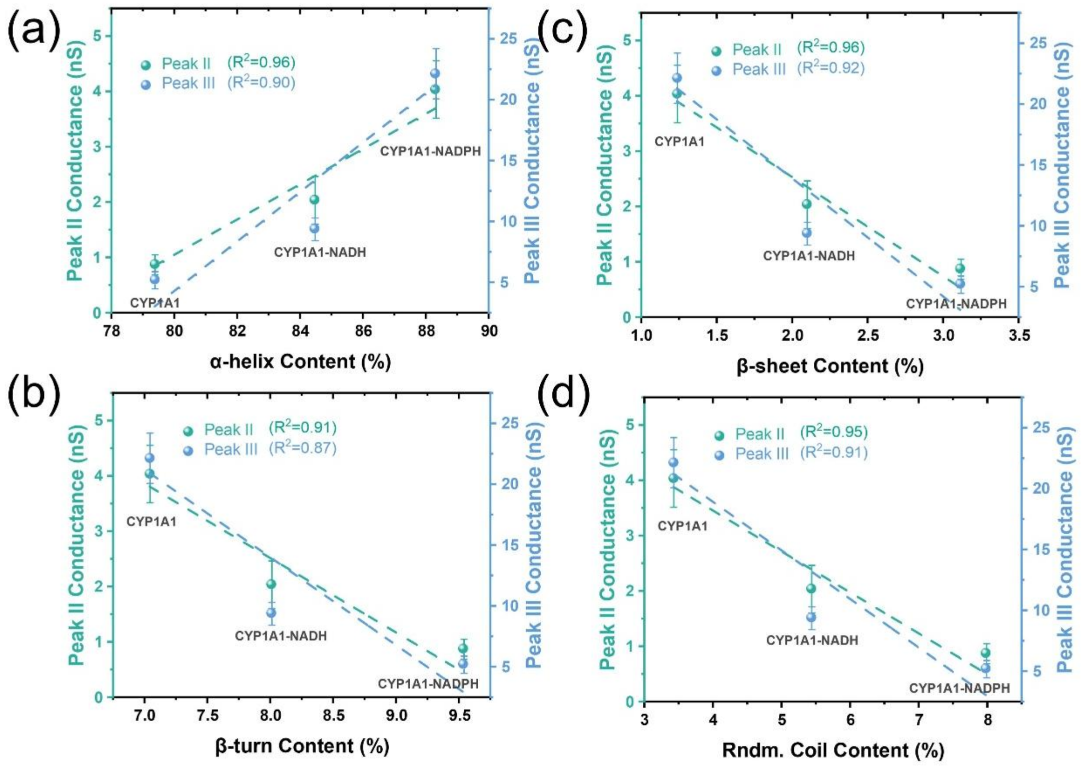

Fig. S21. **Relationship between secondary structures and conductance.** Results show that α-helix **(a)**, β-sheet **(b)**, β-turn **(c)**, and random coil content **(d)** in CYP1A1, CYP1A1-NADH, and CYP1A1-NADPH all correlate negatively with protein conductance. Data points represent mean ± s.d. (n = 3).

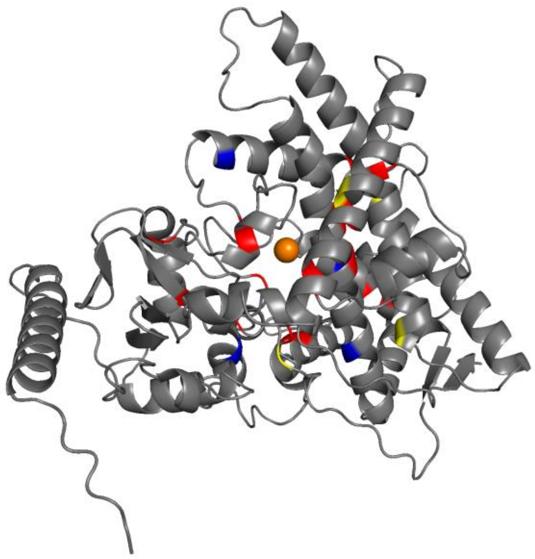

Figure S22. **Aromatic amino acids within 2 nm of Fe^{3+} in the active center of CYP1A1, phenylalanine (red), tryptophan (blue) and tyrosine (yellow)**. These aromatic amino acids encircle the catalytic Fe^{3+} center. The Fe^{2+} LUMO energy aligns

closely with the redox potential of aromatic residues, promoting efficient electron transport.

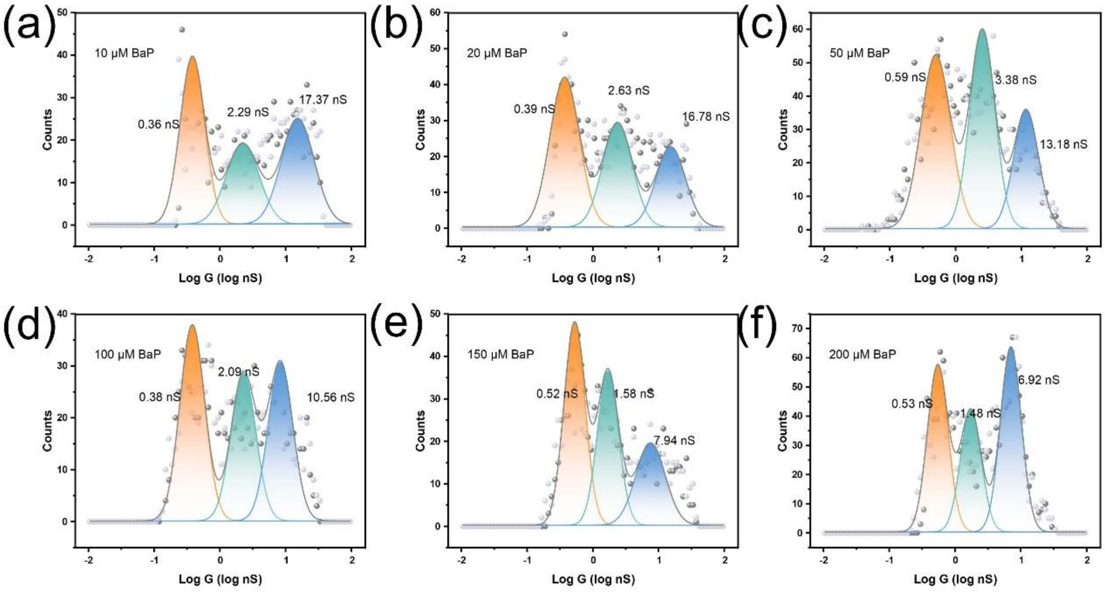

Fig. S23. Conductance distributions of CYP1A1 measured at varying concentrations of BaP. (a-f) The conductance distribution showed three distinct peaks across a BaP concentration range of 10-200 μM. Peaks II and III progressively decreased with increasing BaP concentration, while Peak I remained unchanged.

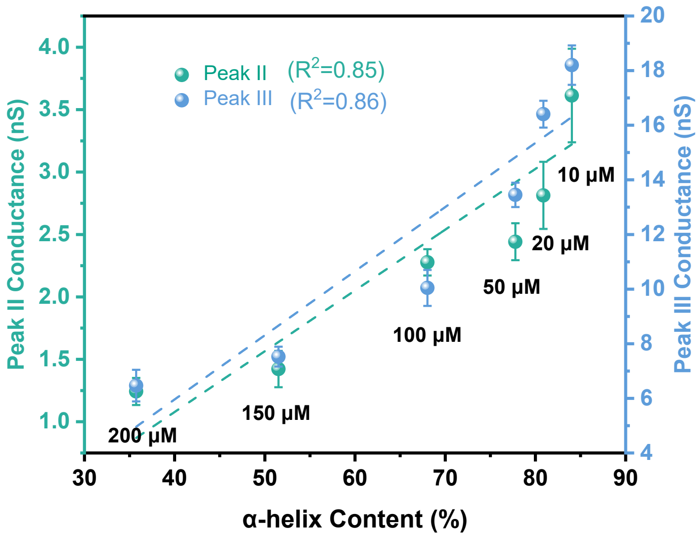

Fig. S24. Positive correlation between α-Helix content and protein conductance (Peaks II and III) in the CYP1A1-NADPH-BaP system. BaP concentrations ranged

from 10 μM to 200 μM. Data points represent mean ± s.d. (n = 3)

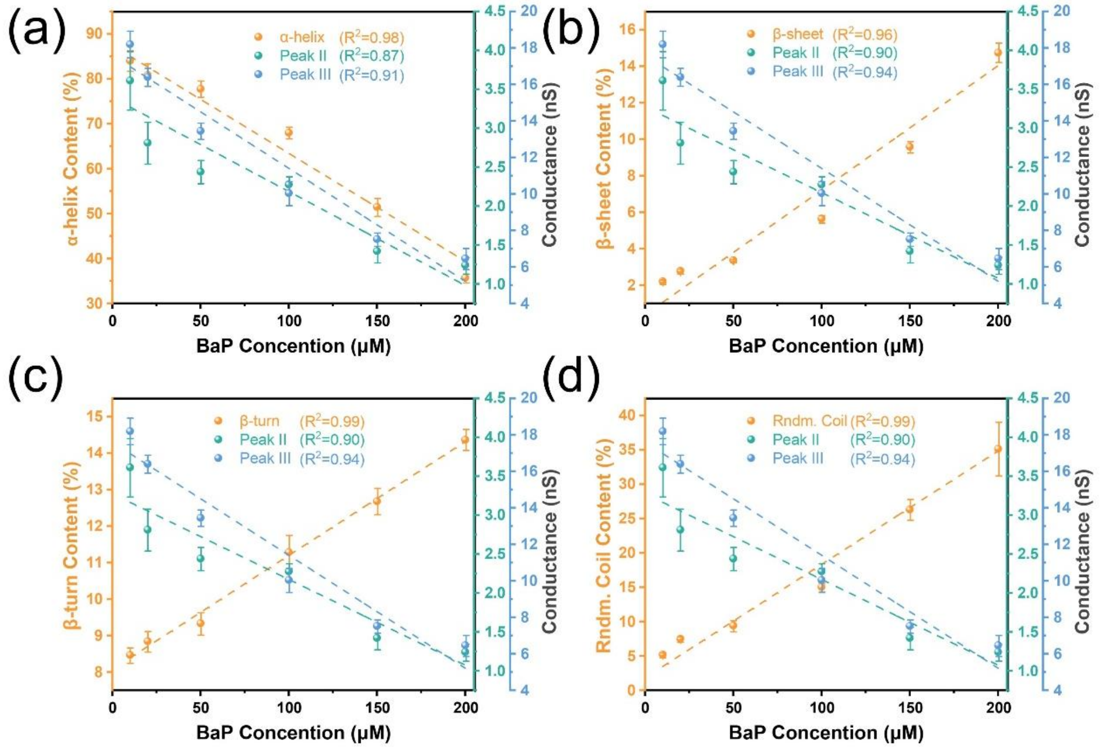

Fig. S25. Relationship between BaP concentration, secondary structure, and conductance. Under varying BaP concentrations, the α-helix **(a)**, β-sheet **(b)**, β-turn **(c)** and random coil **(d)** are all negatively correlated with protein conductance. Data are presented as mean ± s.d. (n = 3).

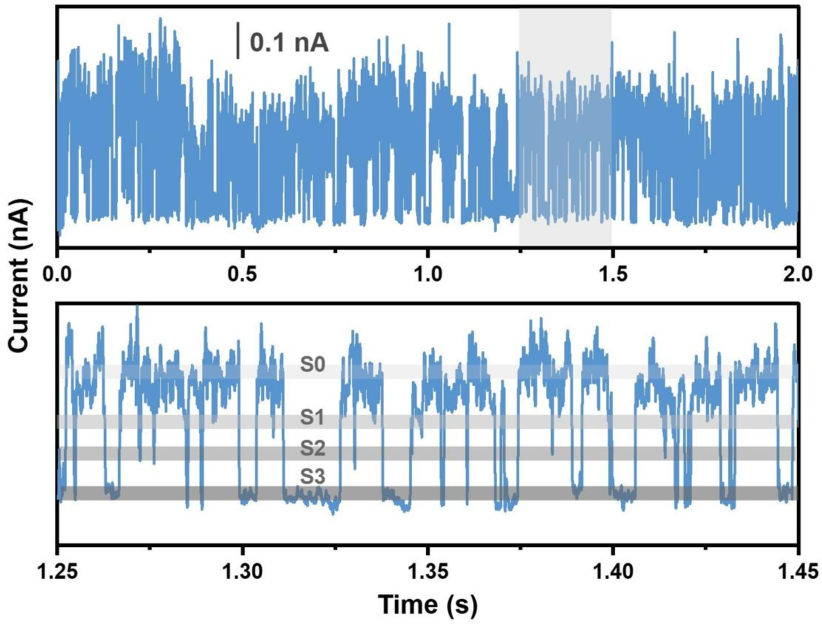

Fig. S26 I-t traces of BaP metabolism, the below panel extracted from the grey area. The four bands in the below panel represent different conductance states,

corresponding to S0-S3 from top to bottom.

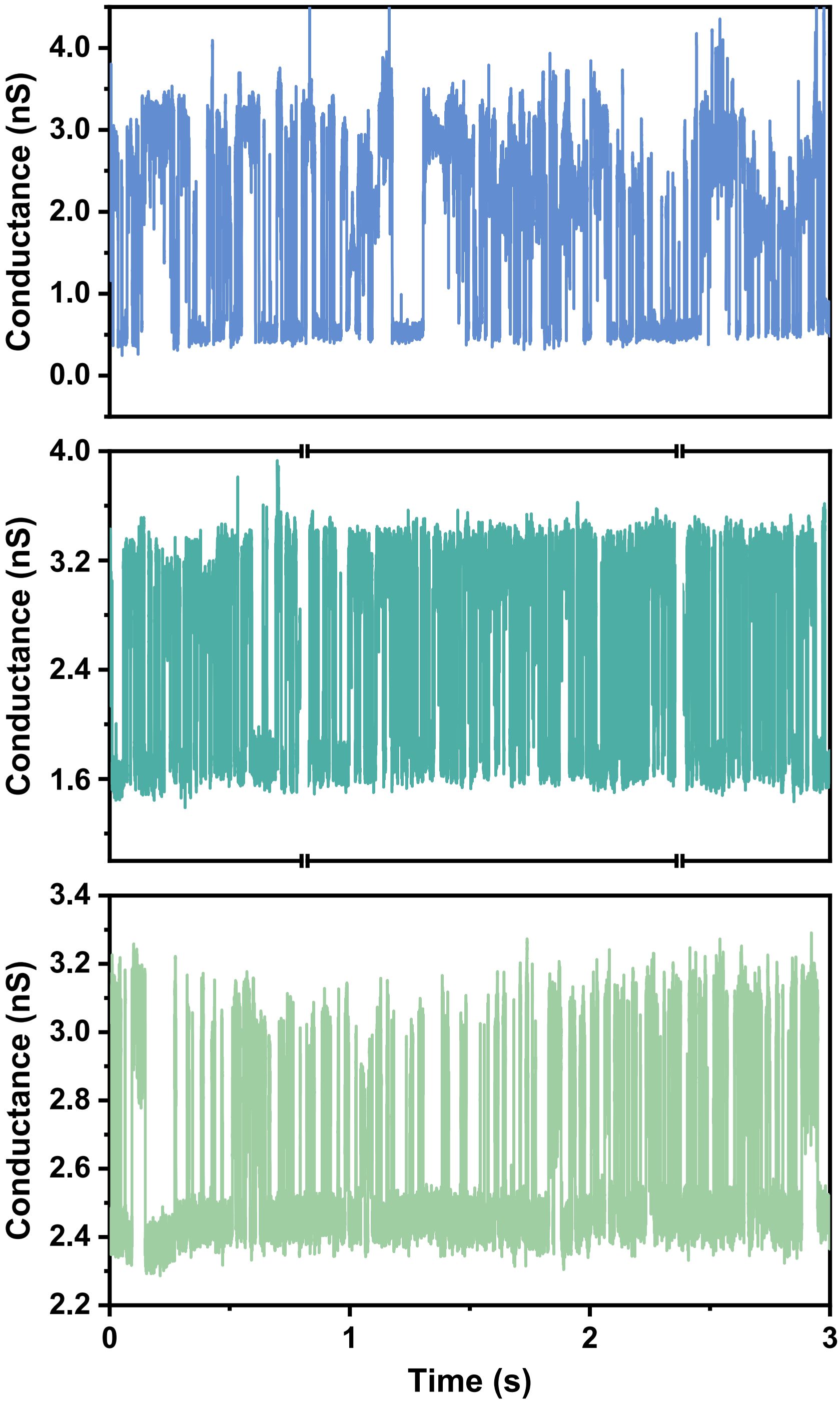

Fig. S27 Representative 3-s i-t traces during CYP1A1-mediated metabolism of BaP, 7,8-D-BaP, and 7-OH-BaP, respectively.

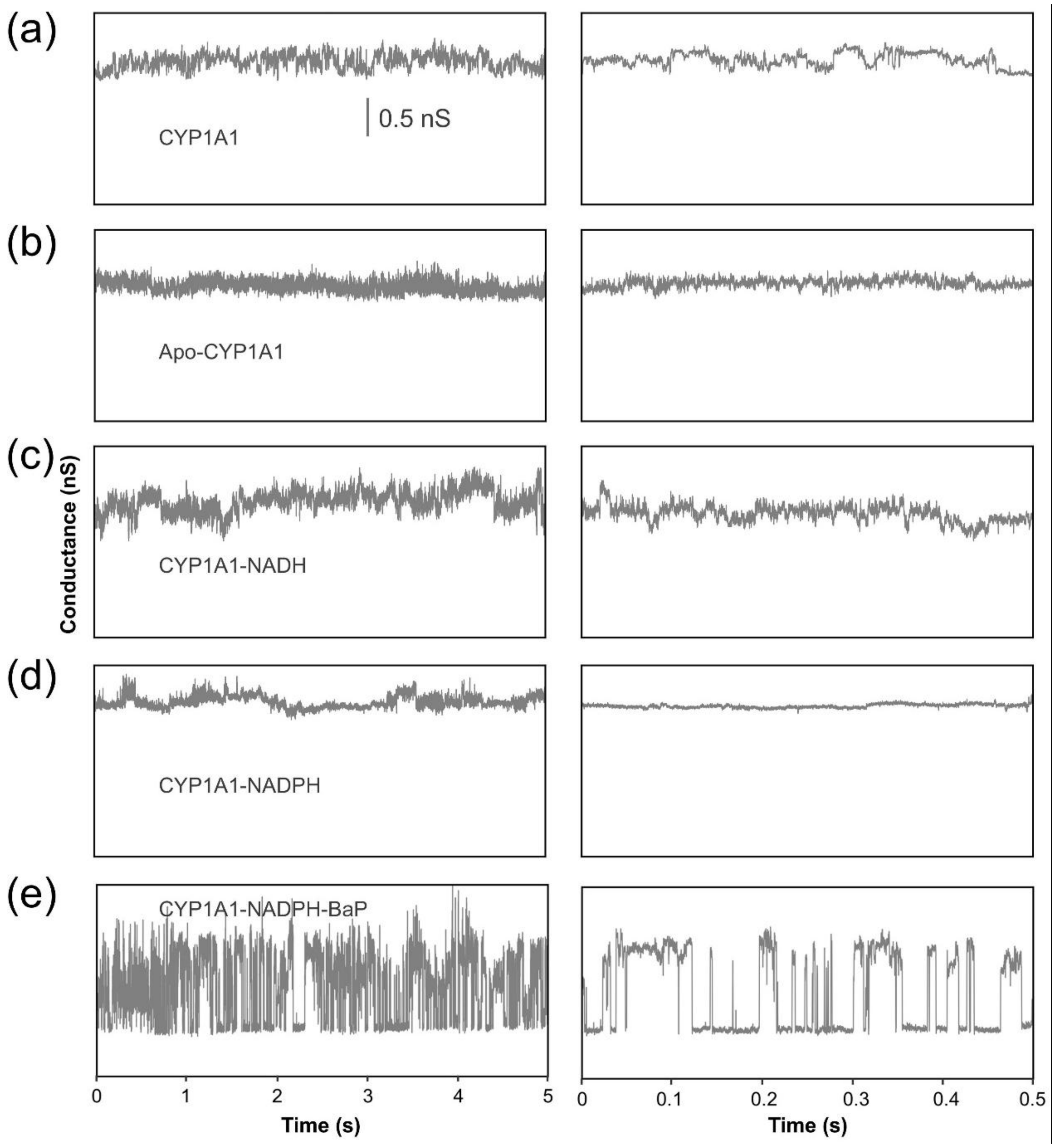

Fig. S28. Representative *I-t* traces of each possible single protein state. (a) CYP1A1, **(b)** Apo-CYP1A1, **(c)** CYP1A1-NADH, **(d)** CYP1A1-NADPH, and **(e)** CYP1A1-NADPH-BaP. In the left panel, the 5-second current-time (*i-t*) trajectories are presented. The right panel displays representative 0.5-second expanded sections randomly chosen from each trajectory.

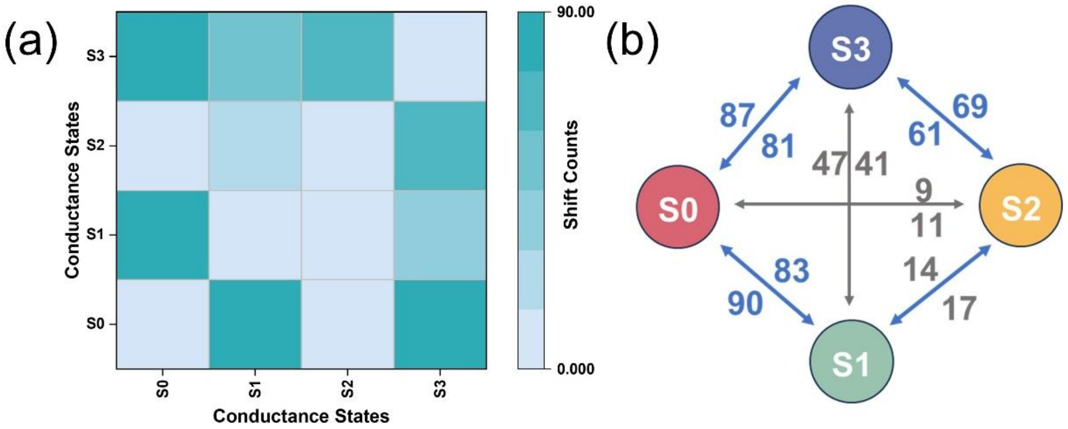

Fig. S29 Analysis of conductance-state transitions during BaP metabolism in *i-t* traces. (a) Transition-density heat-map for the four conductance states. **(b)** Statistical rules governing inter-state conversions.

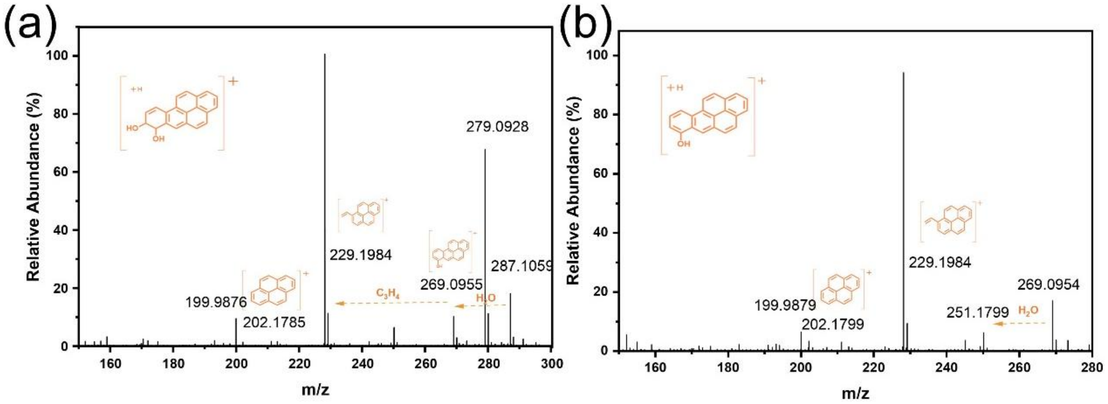

Fig. S30. Representative LC-HRMS spectra of BaP metabolites generated by the CYP1A1-NADPH. (a) Benzo[a]pyrene-7,8-dihydrodiol (7,8-D-BaP) and **(b)** 7-hydroxybenzo[a]pyrene (7-OH-BaP) were identified based on their accurate monoisotopic masses $[M+H]^+$ at m/z 287.1071 and 269.0966, respectively, together with the corresponding diagnostic fragment ions.

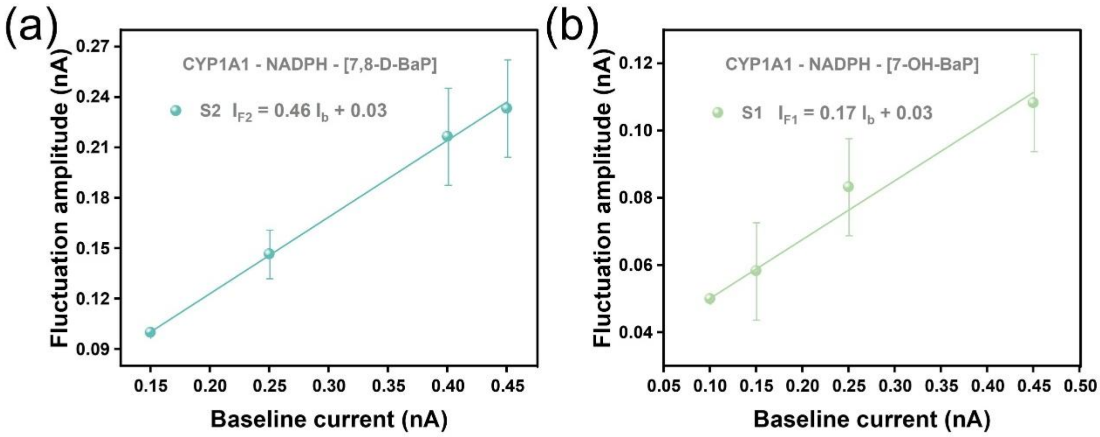

Fig. S31. Linear correlation between telegraph-noise amplitude and baseline current. (a) CYP1A1-NADPH-7,8-D-BaP, and **(b)** CYP1A1-NADPH-7-OH-BaP. (Data presented as mean ± s.d. (n = 3))

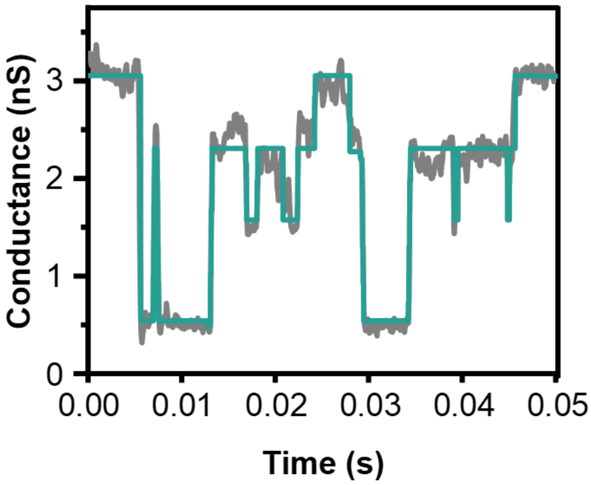

Fig. S32. Example i-t trace segment idealized using a segmental k-means algorithm with hidden Markov modeling, fitted using QUB software (green trace).

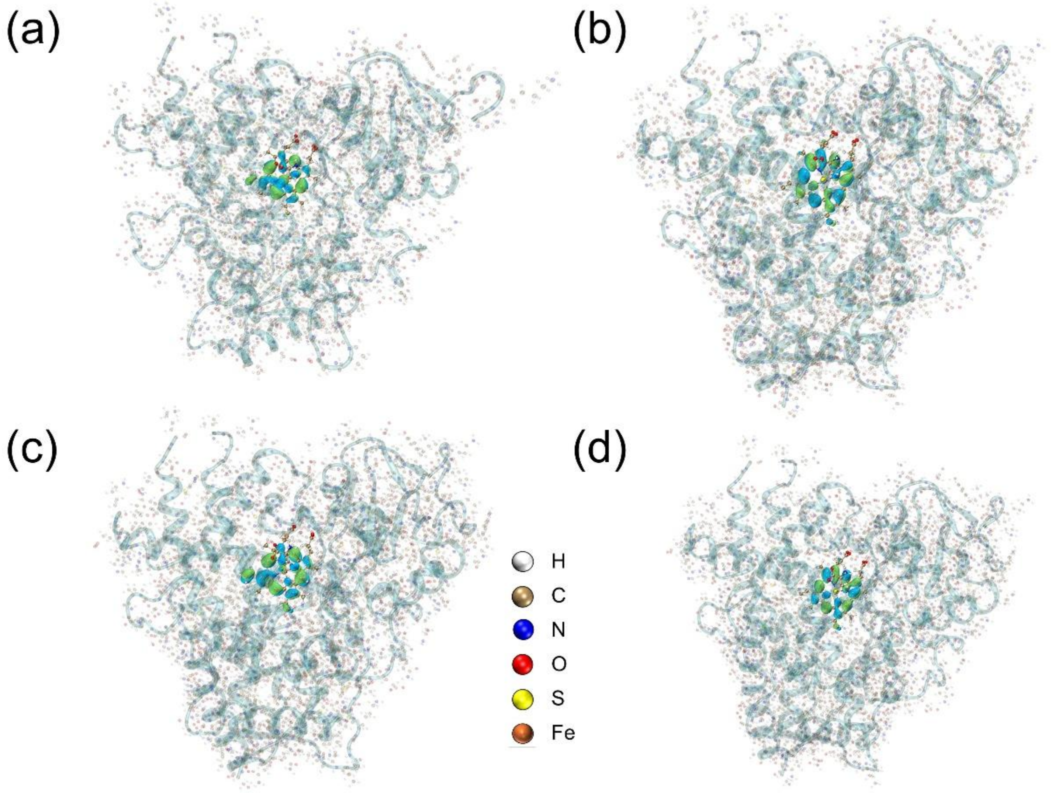

Fig. S33. QM/MM computational model and frontier molecular orbital distributions of the iron-centered ligand. The HOMO and LUMO are shown for the Fe^{2+} (a, b) and Fe^{3+} (c, d) states. The central QM region (ball-and-stick) is embedded within the MM region, which is visualized as translucent background atoms and light blue protein ribbons.

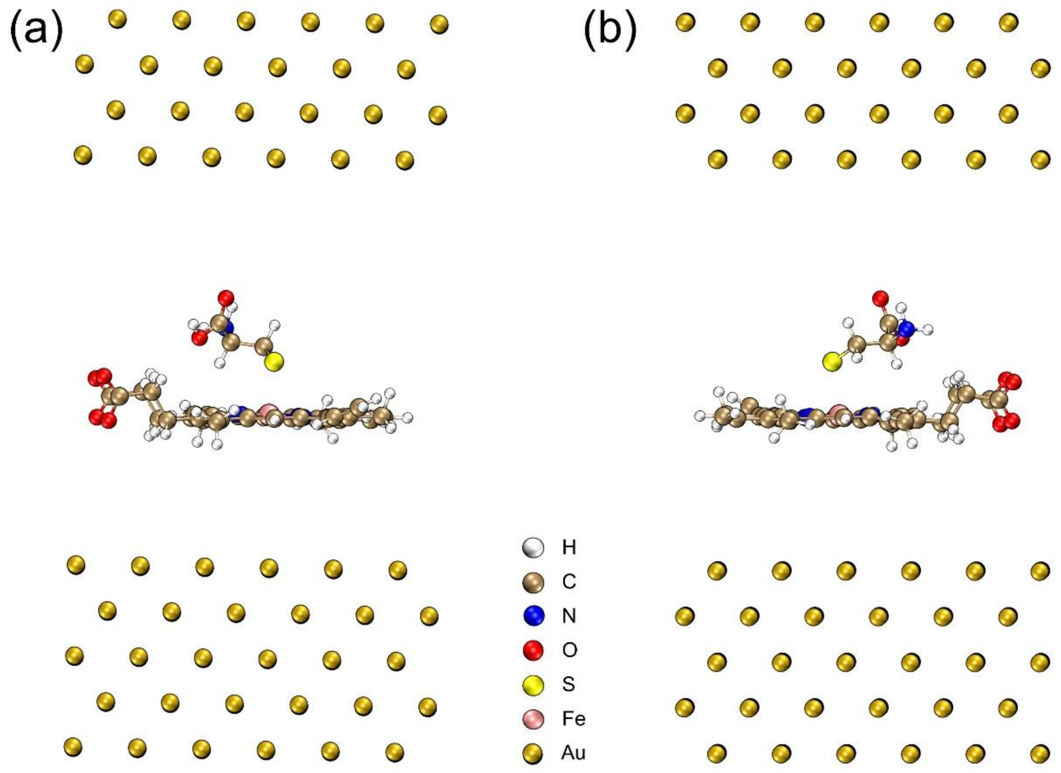

Fig.S34. Two-probe molecular junction models used for transmission spectrum calculations. The central scattering region, comprising the Cys-porphyrin iron complex, is sandwiched between two semi-infinite gold electrodes (shown as spheres). Panels (a) and (b) depict the junction configurations for the Fe^{2+} and Fe^{3+} oxidation states, respectively.

Table S1. Secondary Structure Content of CYP1A1 under Various Conditions

System	α-helix (%)	β-sheet(%)	β-turn(%)	Rndm. Coil (%)
CYP1A1	79.4±3.1	3.1±0.2	9.5±0.09	8.0±0.3
CYP1A1-NADH	84.8±1.6	2.1±0.1	8.0±0.1	5.4±0.1
CYP1A1-NADPH	88.3±1.3	1.2±0.08	7.0±0.07	3.4±0.02
APO-CYP1A1	56.8±1.4	10.8±1.5	17.0±1	15.4±0.7
CYP1A1-NADPH-BaP(10 μM)	84.0±2.3	2.2±0.3	8.4±1.1	5.1±0.4
CYP1A1-NADPH-BaP(20 μM)	80.9±2.5	2.7±0.3	8.8±1.5	7.5±0.2
CYP1A1-NADPH-BaP(50 μM)	77.8±1.8	3.4±0.4	9.3±0.7	9.4±0.4
CYP1A1-NADPH-BaP(100 μM)	68.0±1.3	5.6±0.5	11.3±0.9	15.1±0.7
CYP1A1-NADPH-BaP(150 μM)	51.5±2	9.5±0.6	12.7±1.3	26.3±0.9
CYP1A1-NADPH-BaP(200 μM)	35.7±1	14.7±1	14.4±2	35.2±1.1

Table S2. Exploration of spin multiplicities for iron species

Species	spin multiplicity	<S**2>	Ideal value S*(S+1)	Energy (a.u.)
Fe(II)	1			-3744.23
	3	3.23	2	-3744.34
	5	6.22	6	-3744.35
	7	13.18	12	-3744.33
Fe(III)	2	2.92	0.75	-3743.85
	4	4.92	3.75	-3743.84
	6	8.99	8.75	-3743.85
	8	15.92	15.75	-3743.84

S denotes the spin quantum number. <S**2> represents the total spin operator squared, whose theoretical eigenvalue is given by S(S+1).
