# Supplementary material for: Real-time electrical monitoring of enzymatic catalytic dynamics at the single-molecule level
Source: Nat Commun. 2026 Jun 5;17:7198. doi: 10.1038/s41467-026-74020-0 (PMC13396217; doi:10.1038/s41467-026-74020-0)
Supplement: Supplementary file 2 — Description of Additional Supplementary Files [file 41467_2026_74020_MOESM2_ESM.pdf]

## Description of Additional Supplementary Files

**Supplementary Data 1**: Atomic coordinates of the optimized computational models for the heme iron in the ferric (Fe3+) and ferrous (Fe2+) states derived from the QM/MM electronic structure calculations.

**Supplementary Data 2:** Atomic coordinates of the optimized two-probe molecular junction configurations used for the NEGF-DFT transmission spectrum calculations.
