# Supplementary material for: Real-time electrical monitoring of enzymatic catalytic dynamics at the single-molecule level
Source: Nat Commun. 2026 Jun 5;17:7198. doi: 10.1038/s41467-026-74020-0 (PMC13396217; doi:10.1038/s41467-026-74020-0)
Supplement: Supplementary file 5 — Reporting Summary [file 41467_2026_74020_MOESM5_ESM.pdf]

nature portfolio

Corresponding author(s): Bintian Zhang

Last updated by author(s): May 20, 2026

Reporting Summary

Nature Portfolio wishes to improve the reproducibility of the work that we publish. This form provides structure for consistency and transparency in reporting. For further information on Nature Portfolio policies, see our Editorial Policies and the Editorial Policy Checklist.

Statistics

For all statistical analyses, confirm that the following items are present in the figure legend, table legend, main text, or Methods section.

n/a	Confirmed	
☐	☒	The exact sample size (*n*) for each experimental group/condition, given as a discrete number and unit of measurement
☐	☒	A statement on whether measurements were taken from distinct samples or whether the same sample was measured repeatedly
☒	☐	The statistical test(s) used AND whether they are one- or two-sided<br>*Only common tests should be described solely by name; describe more complex techniques in the Methods section.*
☐	☒	A description of all covariates tested
☐	☒	A description of any assumptions or corrections, such as tests of normality and adjustment for multiple comparisons
☐	☒	A full description of the statistical parameters including central tendency (e.g. means) or other basic estimates (e.g. regression coefficient) AND variation (e.g. standard deviation) or associated estimates of uncertainty (e.g. confidence intervals)
☒	☐	For null hypothesis testing, the test statistic (e.g. *F*, *t*, *r*) with confidence intervals, effect sizes, degrees of freedom and *P* value noted<br>*Give P values as exact values whenever suitable.*
☒	☐	For Bayesian analysis, information on the choice of priors and Markov chain Monte Carlo settings
☒	☐	For hierarchical and complex designs, identification of the appropriate level for tests and full reporting of outcomes
☒	☐	Estimates of effect sizes (e.g. Cohen's *d*, Pearson's *r*), indicating how they were calculated

Our web collection on statistics for biologists contains articles on many of the points above.

Software and code

Policy information about availability of computer code

Data collection	I-V and I-t measurements were achieved by using a PCI-6289 DAQ card (National Instruments) in combination with a LabVIEW program (Version NI LabVIEW 2022 Q3), as described in our previous study (Proceedings of the National Academy of Sciences 116.13 (2019): 5886-5891.).
Data analysis	Conductance measurement results were analyzed and plotted using OriginPro 2021(9.8.0.200). Hidden Markov modeling (HMM) and kinetic simulations were performed using the Quantify Unknown Biophysics (QUB) software (qub.mandelics.com).Theoretical calculations and visualization were performed using ORCA 6.0.1, CP2K 2025.1, Multiwfn 3.8, and VMD 1.9.

For manuscripts utilizing custom algorithms or software that are central to the research but not yet described in published literature, software must be made available to editors and reviewers. We strongly encourage code deposition in a community repository (e.g. GitHub). See the Nature Portfolio guidelines for submitting code & software for further information.

Data

Policy information about availability of data

All manuscripts must include a data availability statement. This statement should provide the following information, where applicable:

- Accession codes, unique identifiers, or web links for publicly available datasets
- A description of any restrictions on data availability
- For clinical datasets or third party data, please ensure that the statement adheres to our policy

The experimental datasets generated and analyzed during the current study, including single-molecule conductance measurements, CD spectroscopy, and enzymatic activity assays, are provided in the Source Data file. The atomic coordinates of the optimized computational models from the electronic structure calculations (QM/MM and NEGF-DFT) are provided as separate plain, unformatted text files in Supplementary Data 1 and Supplementary Data 2. All other relevant data supporting the findings of this study are available within the paper and its Supplementary Information, and from the corresponding author upon request. Source data are provided with this paper.

Research involving human participants, their data, or biological material

Policy information about studies with human participants or human data. See also policy information about sex, gender (identity/presentation), and sexual orientation and race, ethnicity and racism.

Reporting on sex and gender	N.A.
Reporting on race, ethnicity, or other socially relevant groupings	N.A.
Population characteristics	N.A.
Recruitment	N.A.
Ethics oversight	N.A.

Note that full information on the approval of the study protocol must also be provided in the manuscript.

Field-specific reporting

Please select the one below that is the best fit for your research. If you are not sure, read the appropriate sections before making your selection.

☒ Life sciences ☐ Behavioural & social sciences ☐ Ecological, evolutionary & environmental sciences

For a reference copy of the document with all sections, see nature.com/documents/nr-reporting-summary-flat.pdf

Life sciences study design

All studies must disclose on these points even when the disclosure is negative.

Sample size	Sample sizes were determined based on typical single molecule STM studies. For each condition, we recorded I-V traces from at least 1000 individual protein–electrode junctions, which provided sufficient data to obtain stable conductance histograms and statistically robust results.
Data exclusions	Exclusion criteria were pre established. Approximately 20% of recorded I-V traces were excluded from the final analysis due to the unstable molecular junction, as detailed in the manuscript.
Replication	All experiments were independently replicated at least three times using freshly prepared samples, newly etched STM tips, and different protein batches. The measured conductance values and the shape of the I–V characteristics were reproducible across all replicates. All attempts at replication were successful.
Randomization	Randomization was not applicable because the study did not involve allocation to experimental groups. All measurements were performed on the same protein sample under different conditions, and the order of data acquisition was determined solely by the stability of the STM tip and sample.
Blinding	Blinding during data collection was not feasible due to the requirement for real time tip positioning and feedback control. However, data analysis was performed using automated peak finding algorithms and threshold based criteria without manual intervention, minimizing potential bias.

Reporting for specific materials, systems and methods

We require information from authors about some types of materials, experimental systems and methods used in many studies. Here, indicate whether each material, system or method listed is relevant to your study. If you are not sure if a list item applies to your research, read the appropriate section before selecting a response.

Materials & experimental systems

n/a	Involved in the study
☒	☐ Antibodies
☒	☐ Eukaryotic cell lines
☒	☐ Palaeontology and archaeology
☒	☐ Animals and other organisms
☒	☐ Clinical data
☒	☐ Dual use research of concern
☒	☐ Plants

Methods

n/a	Involved in the study
☒	☐ ChIP-seq
☒	☐ Flow cytometry
☒	☐ MRI-based neuroimaging

Plants

Seed stocks	N.A.
Novel plant genotypes	N.A.
Authentication	N.A.
