# Supplementary material for: Real-time electrical monitoring of enzymatic catalytic dynamics at the single-molecule level
Source: Nat Commun. 2026 Jun 5;17:7198. doi: 10.1038/s41467-026-74020-0 (PMC13396217; doi:10.1038/s41467-026-74020-0)
Supplement: Supplementary file 6 — Transparent Peer Review File [file 41467_2026_74020_MOESM6_ESM.pdf]

nature portfolio

Peer Review File

Real-Time Electrical Monitoring of Enzymatic Catalytic Dynamics at the Single-Molecule Level

Corresponding Author: Dr Bintian Zhang

This file contains all reviewer reports in order by version, followed by all author rebuttals in order by version.

Version 0:

Reviewer comments:

Reviewer #1

(Remarks to the Author)
The work reports some interesting results of a metalloenzyme with conductance STM. However, there seems not much new knowledge generated. The is still a lack of fundamental of understanding of the electron tunnelling channel within the specific enzyme and how it changes during catalytic process.

In the introduction section, the authors ignore some important studies of high speed AFM characterization on redox active enzymes.

In Fig 4 for real time measurement, it's expected that the fluctuation should be periodic. But it's quite random signal here. Can the authors explain?

The reviewer doesn't think the calculation of Km for immobilized enzyme make any sense. This is because Michaelis constant is only valid if there is no diffusion limitation.

Reviewer #2

(Remarks to the Author)
In the manuscript entitled "Real-Time Electrical Monitoring of Enzymatic Conformational Dynamics at the Single-Molecule Level", the authors employ a scanning tunneling microscope to investigate the enzymatic catalytic process through measurement of electrical conductance. Some major comments and amendments that need to be addressed are listed below:

1. The authors assert that enzymatic conformational dynamics during the catalytic process can be probed and monitored by utilizing a scanning tunneling microscope (STM) to measure electrical conductance. The author expressed incorrect concepts in the title, abstract, and results section that monitoring of enzymatic conformational dynamics through electrical signal. The authors have merely identified electrical signal variations during the P450 enzyme catalytic process, possibly indicating changes in electron transfer pathways or mechanisms. At present, there exists no theoretical or experimental evidence directly linking conductance changes with conformational dynamics. In contrast, established methods such as NMR, CD, X-ray crystallography, and Cryo-EM are well-validated techniques for studying conformational changes. Therefore, the author needs to revise these parts of the content.

2 “The authors assign Peak I (~0.22 nS)—commonly observed in other proteins such as antibodies and DNA polymerase—to an asymmetric junction configuration involving a specific contact (e.g., an Au–S bond at the substrate) and a nonspecific contact at the probe.” What is the basis for this assignment? Is it the conductance value, peak shape, or another criterion?

3 “It is suggested that the higher conductance Peaks II and III may originate from two specific contacts, given the two surface-exposed cysteine residues (Cys20 and Cys502, Fig. S13) in CYP1A1.” To which specific states or conformations do these two conductance values correspond? Why is there such a significant difference between the conductance values attached to these two cysteines, and how would the conductance change if these cysteines were mutated to residues (such as Ala), which are unable to form specific contacts?

4. In the case of apo-CYP1A1, Peak III disappears, which the authors attribute to the absence of the ferric ion that mediates an efficient charge transport pathway in the holoenzyme. However, if both Peaks II and III indeed arise from specific contacts at Cys20 and Cys502, why does Peak II not just persist but also exhibit a slight rise in conductance?

5. After the addition of the coenzyme NADPH, three conductance peaks remain, all showing increased values compared to those of the enzyme alone. If NADPH was introduced during the measurement, binding would require some time to reach equilibrium. If it was pre-incubated, the system should be at binding equilibrium. Which of the three peaks corresponds to the enzyme–NADPH complex in the bound state?

6. Upon addition of the substrate BaP, three peaks are still observable. Given that both the enzyme and the enzyme–NADPH complex each exhibit three characteristic conductance peaks, why do three new peaks, which differ from the conductance value before, appear during the reaction? Does this imply that the free enzyme and the enzyme–NADPH conformations are no longer present during the catalytic process?

7. There is a clear discrepancy between the conductance test results presented earlier and the subsequent high-resolution current–time (i–t) measurements. Using a “molecular fishing” strategy, the authors identify four states (S0 to S3). How exactly are these four conductance states related to specific stages in the catalytic cycle? Simple logic-based or exclusion-based assignment seems insufficient, as conductance variations can surface from multiple sources.

8. In the section discussing substrate concentration dependence of CYP1A1 conductance, the model mistakenly turns a data processing problem into a scientific problem. The generation of ΔG due to the different ratio of enzyme molecules in the ES state and the original state. But the experiment is performed in the reaction state, thus the identification of the different states' curves with the clustering algorithm is essential and possible to achieve, according to Fig.2b.

9. After the identification of the different states' curves, Fig.3d can be corrected. Now it looks like the logarithmic relationship rather than the linear one. Maybe there are other influencing factors for the reaction not modeled by Michaelis-Menten kinetics?

10. In Fig. 4f, the HMM and QuB software has been employed to idealize your experiment-based curves. How do you define your original transition model for the HMM estimation? In principle, QuB will only generate the segment types according to the number of transition states you define. But in Fig. 4f, the idealized curve shows more plateau types than four states, which is not aligned with the mechanism you proposed.

11. You use two kinds of I-V scan (Fig. 1e) and I-T records (Fig. 4abc). The first mode shows three peaks for each state, while the latter records one representative value per state. Could you clarify the relationship between these two modes and their outcomes? Only the Peak III in the I-V scan is recorded by the i-t curves? Why? And please use the same units (such as nS) in both tests, so that they can be compared.

12. To make it more credible, each possible single state should be tested under the current–time (i–t) measurements.

13. The electronic structure of hemoglobin was calculated using DFT, but the model only included porphyrin and cysteine ligands, thus overlooking protein environment and solvent effects. This omission could compromise energy level accuracy and requires amelioration using the QM/MM method.

Reviewer #3

(Remarks to the Author)
The manuscript reports single-molecule electrical measurements of CYP1A1 during benzo[a]pyrene metabolism, identifying several conductance states that are assigned to catalytic intermediates. The experimental work is technically adequate and addresses an important challenge in enzymology. The results are interesting, and, in my view, the manuscript could be suitable for publication in Nature Communications. However, several aspects require clarification or further discussion before acceptance.
1. My main concern is that the experimental observations are not sufficiently supported by a microscopic interpretation. The manuscript detects conductance changes and associates them with structural rearrangements, but no structural or computational analysis is provided to support such assignments. The work would benefit from a clearer mechanistic rationale explaining how the different conductance states relate to plausible conformational changes in CYP1A1, and from stronger connections with previous mechanistic or computational studies on P450 enzymes. At present, the interpretation is largely based on correlations, without independent evidence to validate the proposed sequence of states.
2. There are also some internal inconsistencies. Peak III is first attributed to contact geometries involving surface cysteines (Cys20 and Cys502), later to Fe-mediated charge transport (based on the absence of Peak III in the apo enzyme), and later again to conformational changes around the active site. These explanations are not fully compatible. The presence of the heme in the holo enzyme could itself induce conformational rearrangements that modify the protein–electrode contacts, which may explain the extra peak without invoking metal-mediated hopping. The discussion oscillates between “conformational effects” and “redox effects”, but the causal separation between them is not clearly established.
3. The manuscript further assumes that NADPH binding does not induce conformational changes because the substrate is absent. This is not necessarily correct, as cofactor binding often affects the structure or dynamics of P450 systems. Since this assumption is used to attribute the conductance enhancement solely to the redox change, it should be justified or treated

with more caution.
4. On the computational side, the DFT calculations are based on a highly truncated model (porphyrin + axial cysteine), which may not describe correctly the spin state or orbital distribution. The use of B3LYP for Fe(III)/Fe(II) redox systems is also questionable. Importantly, no transport-related quantities are computed, so the calculations do not directly support the interpretation of the conductance. At minimum, the limitations of this model should be explicitly addressed.
5. The kinetic analysis also requires clarification. The approximation that the change in conductance is proportional to the ES population is strong, especially in single-molecule experiments where conductance depends on contact geometry and molecular orientation. Moreover, the different Km values obtained (from conductance, GC-MS, and Lineweaver–Burk) differ by more than a factor of three. These discrepancies should be discussed more directly rather than simply stating that they are of the same order of magnitude.
6. Error bars must be included in all the data presented along the manuscript.
7. Finally, several conclusions in the manuscript rely on attributing conductance changes to a single effect (redox state, conformational change, or contact geometry), but the experiments do not fully isolate these contributions. A more critical discussion of these limitations would improve the clarity and credibility of the work.
In summary, I recommend publication after major revisions that strengthen the mechanistic interpretation, clarify the logic used to assign conductance states, and discuss more explicitly the limitations of the experimental and computational analyses.

Reviewer #4

(Remarks to the Author)
I co-reviewed this manuscript with one of the reviewers who provided the listed reports. This is part of the Nature Communications initiative to facilitate training in peer review and to provide appropriate recognition for Early Career Researchers who co-review manuscripts.

Version 1:

Reviewer comments:

Reviewer #1

(Remarks to the Author)
The reviewer is happy with the changes.

Reviewer #2

(Remarks to the Author)
This manuscript presents a conceptually appealing and innovative idea—"Electrical Monitoring of Enzymatic Catalytic Dynamics at the Single-Molecule Level." However, despite the novelty of the concept, I have substantial concerns regarding the validity of the experimental observations and the robustness of the data analysis. A critical issue arises from the authors' own acknowledgment that "Given the limited conductance resolution of the I-V histogram, the conductance features of the enzyme-NADPH complex and the ES complex are unlikely to be resolved as separate, well-defined peaks." This admission fundamentally undermines the central claim of the study. If the conductance technique lacks the resolution to distinguish between key catalytic states, it raises serious doubts as to whether the observed signals genuinely reflect dynamic enzymatic catalytic processes or merely arise from unresolved, averaged events. Furthermore, the data processing methodology exacerbates these concerns, suggesting that the observed "states" may represent ensemble averages rather than discrete, functionally relevant intermediates. One of the major scientific issues is that the authors mistake the duration distribution and dwell time/lifetime distribution. Based on this misunderstanding, their duration distribution follows the exponential distribution rather than bell-shape, increasing the unreliability of their work. Given these fundamental limitations, it remains unclear whether the electrical signals reported indeed capture genuine catalytic dynamics at the single-molecule level.

1. Regarding Fig. R3c (Fig 2c), the R^2 fit based on only three data points lacks sufficient statistical significance and does not support the conclusion of a strong correlation. Furthermore, I have substantial concerns about Fig. R3def (Fig 2def). First, α-helical structures in enzymes undergo continuous bending, unfolding, and refolding during catalysis, processes closely tied to catalytic functon(Biochemistry 2025, 64, 4281−4295). This implies that the proportion of α-helical content in an enzyme fluctuates dynamically throughout the catalytic cycle. Given that a single circular dichroism (CD) measurement takes approximately 5–10 minutes, and that both the reaction rate and total reaction time vary with BaP concentration, a critical question arises: do the α-helical percentages obtained from CD measurements at six different BaP concentrations represent the state before, during, or after the reaction? Can these values be considered comparable across different states? The CD experiments merely indicate that the α-helical content changes during the reaction; they do not substantiate the claimed correlation.

2. Although your explanation—"Given the limited conductance resolution of the I-V histogram, the conductance features of the enzyme-NADPH complex and the ES complex are unlikely to be resolved as separate, well-defined peaks"—may be an honest acknowledgment, it raises a concerning issue. The limited resolution may cause multiple states to merge into a single peak, obscuring the true underlying scientific reality. This could even lead to erroneous conclusions when such data are used as the basis for analysis (see comment 3). A similar concern arises: could this averaging effect, which masks rare

events due to insufficient resolution, also be present in other experimental measurements?

3. The amplitude-based analysis is concerning. In response to Point 6, the authors concede that the I-V method suffers from insufficient precision, such that conductance signals from distinct real-world events are averaged into peak shifts. Consequently, states exhibiting high-amplitude electronic fingerprints in I-t mode are more likely to represent a superposition of multiple substates—potentially corresponding to either the enzyme-NADPH complex or the ES complex. As long as conductance values cannot be decomposed into individual single-molecule events, subsequent analyses and interpretations, particularly the identification of S3, remain problematic.

4. The authors acknowledge that due to limitations in data analysis techniques, the observed peak shift represents an average of two types of events. However, the nature of this averaging remains ambiguous: is it a linear average of the two states or a nonlinear one? Given that both the E and ES complexes exhibit two peaks each, even a linear average would require appropriate weighting. In any case, ensemble averaging compromises the credibility of the analysis presented in this section.

5. Moreover, given the admission that the conductance value reflects an average of multiple events, certain statements in this section warrant reconsideration. For example: "This substrate-dependent conductance change is consistent with the variation in α-helical content extracted from CD measurements (Fig. 2f), yielding linear correlations for both Peaks II and III (Fig. S24-S25)." Similar concerns apply to the section title "Substrate concentration dependence of the CYP1A1 conductance" and the definition of ΔG.

6. Closely related to the previous point, without resolving the sub-states within each peak, it is difficult to determine whether the observed effects stem from data analysis limitations or from diffusion-related processes.

7. Although the authors reanalyzed the data using QUB and HMM, they did not update the dwell time statistics accordingly. Modifying the initial model and parameters in QUB inevitably alters the idealized trace, and thus the dwell time distributions must also change.

8. Given the unreliability of amplitude-based analysis, states exhibiting high-amplitude electronic fingerprints likely consist of multiple sub-states. Therefore, the reported lifetimes correspond to the aggregate of multiple events, rendering the statistical analysis of S3 lifetimes unreliable.

9. The definition of lifetime/dwell time used in the study is problematic. Dwell time should typically refer to the residence time in a specific state before transitioning to another state—not the distribution of all sojourn times within a given state. Based on the state transition matrix provided by the authors, there should be $4 \times 4 - 4 = 12$ distinct dwell times.

10. The fitting of dwell time distributions is also unreliable. A proper dwell time distribution should follow a single-exponential decay. However, the authors appear to have constructed a duration histogram, which is expected to exhibit a bell-shaped distribution.

11. For I-t measurements, please provide absolute conductance values instead of only scale bars. Absolute conductance is essential for assessing the stability and reliability of the measurements. Throughout the manuscript, I-t traces are presented with scale bars but without absolute values.

12. Absolute conductance values should be reported for the I-t measurements. Do the absolute conductance values observed in single-state measurements fall within the amplitude range of the high-amplitude state?

Reviewer #3

(Remarks to the Author)
The authors have addressed all the points raised in my previous report. In my opinion, the paper only needs to improve the description of the methods employed, at least regarding the computational simulations.
In the Methods section it is first stated that “ORCA 6.0.156 was used for all quantum-chemical calculations in this study”, but later it is said that “CP4K 4045.160 was used for all first-principles calculations in this study”. The authors should clarify which methods were employed for each type of calculation. In addition, they need to provide more details about which atoms were included in the QM region (in QM/MM calculations) and which atoms were considered in the QM calculations. A figure in the Supporting Information could be useful for this purpose.
Finally, the authors should provide the key structures in a public repository to facilitate reproducibility.

Reviewer #4

(Remarks to the Author)
I co-reviewed this manuscript with one of the reviewers who provided the listed reports. This is part of the Nature Communications initiative to facilitate training in peer review and to provide appropriate recognition for Early Career Researchers who co-review manuscripts.

Version 2:

Reviewer comments:

Reviewer #2

(Remarks to the Author)
I have no additional comments on this manuscript and recommend that it be accepted for publication.

Open Access This Peer Review File is licensed under a Creative Commons Attribution 4.0 International License, which permits use, sharing, adaptation, distribution and reproduction in any medium or format, as long as you give appropriate credit to the original author(s) and the source, provide a link to the Creative Commons license, and indicate if changes were made.
In cases where reviewers are anonymous, credit should be given to 'Anonymous Referee' and the source.
The images or other third party material in this Peer Review File are included in the article’s Creative Commons license, unless indicated otherwise in a credit line to the material. If material is not included in the article’s Creative Commons license and your intended use is not permitted by statutory regulation or exceeds the permitted use, you will need to obtain permission directly from the copyright holder.
To view a copy of this license, visit https://creativecommons.org/licenses/by/4.0/

Point-to-point responses to the reviewers' comments

We sincerely thank the reviewers for their insightful comments and valuable suggestions. We have carefully considered each point and revised the manuscript accordingly. Our point-by-point responses are provided below. For clarity, reviewer comments are presented in plain text, followed by our responses in blue, and the revised text in the manuscript is shown in *italics*.

Reviewer #1 (Remarks to the Author):

General Comments: The work reports some interesting results of a metalloenzyme with conductance STM. However, there seems not much new knowledge generated. The is still a lack of fundamental of understanding of the electron tunnelling channel within the specific enzyme and how it changes during catalytic process.

Response: We sincerely thank the reviewer for the insightful comments regarding the fundamental nature of the electron tunneling channels and their evolution during the catalytic process. We fully agree that providing a deeper mechanistic understanding of how specific enzyme states modulate charge transport is essential. To address this, we have incorporated new experimental evidence (circular dichroism spectroscopy and additional control experiments) together with theoretical analyses (QM/MM and NEGF-DFT calculations) to explicitly correlate conductance variations with defined structural and electronic changes in CYP1A1. Our detailed responses are provided below.

(1) Elucidation of distinct electron tunneling channels

To establish the physical origin of the observed conductance states, we systematically analyzed the transport pathways and identified two distinct tunneling channels.

a) The metal-mediated pathway (Peak III): This high-conductance state (4.47 nS) is dependent on the heme iron (Fig. 1g). Control experiments with iron-depleted Apo-CYP1A1 show the complete disappearance of Peak III (Fig. 1h), indicating that Peak III corresponds to a charge transport pathway through the metal active center.

b) The protein-matrix pathway (Peak II): This intermediate state (0.93 nS) persists even in the absence of the metal center but requires specific cysteine contacts (Fig. 1h). This represents a tunneling pathway through the peptide backbone and aromatic networks, independent of the heme iron.

c) Contact dependence: Both pathways depend on strong chemical coupling through Au-S bonds. To verify this contact dependence, we performed an additional control experiment using an STM tip passivated with mercaptoethanol (MCE). Under these conditions, both Peak II and Peak III disappeared (Fig. S13), indicating that these high-conductance channels require specific anchoring through surface-exposed cysteine residues.

(2) Modulation of conductance via conformational gating

To correlate enzyme structural changes with conductance, we performed CD spectroscopy under different catalytic conditions, including apo-enzyme, holo-enzyme, and complexes with cofactors (NADH/NADPH) and at varying concentrations of BaP (Fig. R1, also Fig. 2 in the main text and Figs. S20-S21, S24-S25 in the SI). Quantitative analysis of the enzyme's secondary structures reveals a linear and positive correlation between α-helical content and the measured conductance (Fig. 2c).

Crystallographic studies show that the active-site pocket of CYP1A1 is framed by the F and I helices (*J. Biol. Chem.* 282, 14348 (2007)), which host dense networks of aromatic residues (e.g., Tyr, Trp, and Phe) that are known to facilitate electron tunneling (*ACS Phys. Chem Au* 3, 444-455 (2023) and *Proc. Natl. Acad. Sci.*121, e2405156121 (2024)). Our CD results indicate that NADPH binding stabilizes a more ordered, catalytically competent conformation with increased α-helicity, whereas binding of the bulky substrate BaP induces partial unwinding or rearrangement of these helices to accommodate the ligand. Such induced-fit remodeling is expected to modify the tunneling barrier within the protein junction, thereby altering the charge transport efficiency. This provides experimental evidence that the measured conductance is modulated by conformational changes of the enzyme during the catalytic cycle.

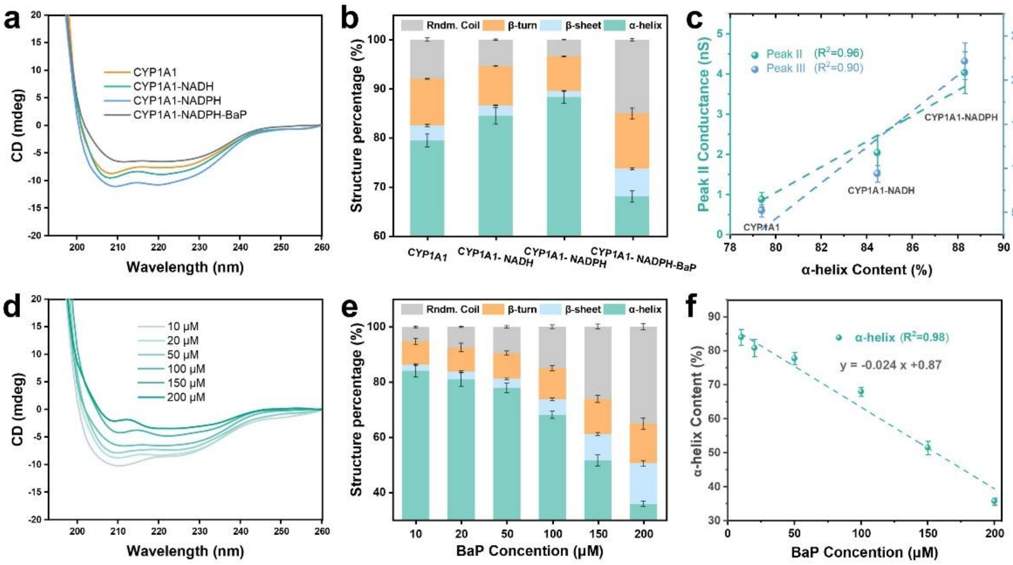

Fig. R1 Relationship between protein secondary structure and conductance under different catalytic conditions. (a, b) CD spectra and corresponding secondary-structure contents of CYP1A1, CYP1A1-NADH, CYP1A1-NADPH, and CYP1A1-NADPH-BaP. (c) Correlation between α-helical content and measured conductance. (d, e) CD spectra and secondary-structure analysis of CYP1A1-NADPH at different BaP concentrations. (f) Dependence of α-helical content on BaP concentration. Data are presented as mean ± s.d. (n = 3).

(3) Effect of heme redox state on protein conductance

To explain how the catalytic process alters the charge transport channels at the electronic level, we performed QM/MM calculations on the full protein-solvent system (See Method in the main text). It shows that the reduction of the heme iron from Fe^{3+} to Fe^{2+} (induced by NADPH binding) results in a narrowing of the HOMO-LUMO gap. Importantly, the LUMO of the Fe^{2+} state shifts to align energetically with both the Fermi level of the gold electrode and the redox potential levels of the surrounding aromatic residues. (Fig. 3c and Fig. S22 in the SI). Quantum transport calculations (NEGF-DFT) further confirm that this alignment results in a pronounced increase in zero-bias transmission for the Fe^{2+} state relative to Fe^{3+} state (Fig. 3b), providing a fundamental electronic-structure explanation for the high-conductance Peak III observed experimentally.

Collectively, these integrated results indicate the conductance features to distinct transport pathways and demonstrate how catalytic events, specifically conformational

changes and redox chemistry, significantly modulate the efficiency of these pathways. We hope that these clarifications and the new data provided effectively address the reviewer's concerns regarding the fundamental understanding of electron transport in CYP1A1.

1. In the introduction section, the authors ignore some important studies of high speed AFM characterization on redox active enzymes.

<u>Response:</u> We apologize for the oversight and thank the reviewer for pointing out these important studies. We fully agree that High-Speed Atomic Force Microscopy (HS-AFM) represents a pioneering technique for directly visualizing the conformational dynamics of proteins, and that these contributions are essential for appropriately contextualizing the present study. As suggested, we have now incorporated these important studies into the Introduction Section, as follows:

"Furthermore, high-speed AFM (HS-AFM) provides exceptionally high spatiotemporal resolution, enabling the direct, real-time observation of protein dynamic processes under near physiological conditions[15-17]*."* (Page 5, lines 79-81)

2. In Fig 4 for real time measurement, it's expected that the fluctuation should be periodic. But it's quite random signal here. Can the authors explain?

<u>Response:</u> We thank the reviewer for this insightful comment. The fluctuations appear relatively stochastic rather than strictly periodic could be rationalized by the fact that single-molecule enzymatic turnover is inherently probabilistic (*Nat. Catal.* 6, 266-275 (2023)), particularly for a complex metabolic enzyme such as CYP1A1, whose catalytic cycle involves branching reaction pathways (*ACS Catal.* 8, 10964-10976 (2018)).

Unlike a simple reaction governed by a single rate-limiting step, the metabolism of BaP branches into competing activation (epoxide formation) and detoxification (phenol formation) pathways. Consequently, the enzyme does not follow a rigid, repetitive sequence of states (e.g., A→B→C→A). Instead, after the initial substrate binding (State S0), the enzyme stochastically "selects" a pathway based on its instantaneous

conformation and local reactant availability, transitioning to either State S3 (activation) or State S1 (detoxification) with different probabilities (*Chem. Res. Toxicol.* 29, 1325-1334 (2016)).

To quantitatively assess this behavior, we performed a statistical analysis of state-to-state transitions based on more than 1500 events collected from ~50 measurements. The resulting transition-density heatmap (Fig. R2, also Fig. S28 in the SI) reveals a clear underlying kinetic organization: transitions originate predominantly from the baseline state S0 and bifurcate into S1 or S3, consistent with the parallel formation of distinct metabolic intermediates. Subsequent transitions from S3 to S2 are also prominent, corresponding to a downstream catalytic step along the activation pathway. In contrast, transitions such as S0→S2 and S1→S2 occur only rarely, likely reflecting off-pathway processes such as premature substrate dissociation or transient trapping of an alternative intermediate (*Environ. Sci. Technol.* 50, 8155-8163 (2016)).

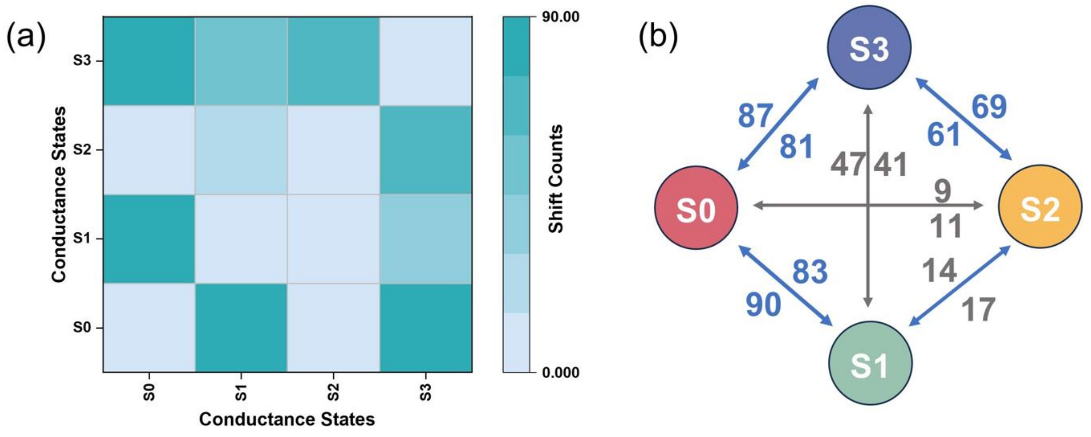

Fig. R2 (Also Fig. S28 in the revised Supporting Information) (a) Heatmap of transition density between different conductance states derived from the same *i*-*t* curves, and (b) statistics of transition counts, where directional arrows and numerical values indicate the transition direction and frequency, respectively.

Thus, the apparent "randomness" in the time traces may reflect the intrinsic stochasticity and kinetic partitioning of a single enzyme molecule among competing catalytic routes in real time. We have incorporated this additional analysis and discussion into the revised manuscript. (Page 20, lines 478-495; Page 23, lines 543-545 in the revised manuscript)

3. The reviewer doesn't think the calculation of Km for immobilized enzyme make any sense. This is because Michaelis constant is only valid if there is no diffusion limitation.

Response: We thank the reviewer for this insightful comment regarding the validity of the Michaelis-Menten kinetic analysis to surface-confined enzymatic systems. We fully agree that, in the presence of strong mass-transport limitations, the intrinsic K_m cannot be directly extracted, as the observed kinetics may be dominated by diffusion rather than enzymatic turnover.

Nevertheless, we respectfully suggest that, under our experimental conditions, the determination of an apparent K_m could remain physically meaningful and useful for characterizing the system. In homogeneous catalysis, the reaction is ideally under kinetic control, whereas in heterogeneous configurations (e.g., ELISA assays, surface plasmon resonance, and biosensors) mass transport can contribute significantly, potentially leading to diffusion-limited behavior. The relative importance of reaction versus diffusion is commonly assessed by the Damköhler number (*Da*), which quantifies the ratio of the maximum reaction rate to the mass-transport rate (*Nat. Biotechnol.* 26, 417-426 (2008)):

$$Da = \frac{V_{max}}{K_L K_m} = \frac{K_{cat}\Gamma}{(D/\delta)K_m}$$

Where K_{cat} is the turnover number (≈ 2 s^{-1}， *J. Electroanal. Chem.* 804, 23-28 (2017)), Γ is the surface coverage of the immobilized enzyme, D is the diffusion coefficient of the substrate (≈ 3.3 × 10^{-6} cm^2 s^{-1}， *J. Chem. Eng. Data* 1994, 39, 2, 281-285), δ is the diffusion layer thickness (assumed to be ≈ 200 μm， *Arch. Pharmacol.* 304, 175-181 (1978) and *Int. J. Pharm.* 71, 55-64 (1991)), K_m is the intrinsic Michaelis constant.

Assuming a closely packed monolayer, the surface coverage can be estimated from the molecular footprint of CYP1A1 (19.5 × 23.5 nm^2, i.e., A_{nez} ≈ 458.25 nm^2) as

$$\Gamma = \frac{1}{N_A A_{enz}} \approx 3.62 \times 10^{-13}\ mol\ cm^{-2}$$

Using an intrinsic K_m of 19 μM (*J. Electroanal. Chem.* 804, 23-28 (2017)), the resulting *Da* is approximately 0.23. This places our system in the mixed kinetic-diffusion control regime (0.1 < Da < 10), rather than in the regime of complete diffusion control (Da ≫ 10). In this intermediate regime, particularly at relatively small *Da* values,

the Michaelis-Menten model remains a valid and widely used approximation for benchmarking enzymatic activity in surface-based assays and biosensors (*Anal. Chem.* 89, 10311-10320 (2017) and *Anal. Chem.* 85, 4546-4553 (2013)), although the extracted parameter should be regarded as an apparent constant, K_m^{app}, expected to be larger than the intrinsic value of roughly $(1 + Da) \approx 1.23$. This indicates that mass-transport effects play a minor role in the observed kinetics.

This theoretical assessment is consistent with our experimental observations. The conductance change (ΔG) exhibits a clear hyperbolic saturation with increasing BaP concentration (Fig. 4c), rather than the linear dependence characteristic of purely diffusion-limited processes. Moreover, the K_m^{app} values obtained from the conductance measurements (24.2-43.2 μM) are in close agreement with reported values for free CYP1A1 in solution (19.0-25.6 μM, *J. Electroanal. Chem.* 804, 23-28 (2017). Such correspondence indicates that, within our experimental window, the reaction is not mass diffusion-limited.

A key factor may be due to the geometry of the system, where the enzyme forms a self-assembled monolayer on a planar gold surface and is directly exposed to the bulk solution, in contrast to conventional enzymatic immobilization in porous beads or gels, where severe intra-pore diffusion resistance is present. Furthermore, immobilization via EDC/NHS coupling with MPA spacers minimizes direct metal-protein contact and preserves enzymatic activity, as previously demonstrated in our study of DNA polymerase (*ACS Nano* 14, 1360-1368 (2020)).

We thank the reviewer again for this important and constructive comment, and we hope that this clarification adequately addresses the concern. To improve accuracy and scientific rigor, we now consistently refer to the extracted parameter as the apparent Michaelis constant, K_m^{app}, in the revised manuscript. We have also added a discussion of mass-transport effects and a Damköhler number analysis to clarify the kinetic regime and support a more rigorous interpretation. (Pages 18-19, lines 436-449).

"Under these conditions, the observed reaction rate is governed by both intrinsic catalytic kinetics and the mass transport of substrate to a spatially confined active site.

To quantify the influence of diffusion, we calculated the Damköhler number (Da) and obtained a value of approximately 0.23 (Methods). This places the system in the mixed kinetic-diffusion regime (0.1 < Da < 10), rather than in the limits of purely kinetic-controlled (Da ≪ 0.1) or purely diffusion-controlled (Da ≫ 10) behavior. In this regime, the experimentally determined K_m^{app} *is expected to be larger than the intrinsic kinetic constant by a factor of approximately (1 + Da) ≈ 1.23. These results collectively demonstrate that the conductance response of CYP1A1 provides a sensitive and quantitative measure of enzymatic kinetics. This establishes a robust framework for employing protein-based bioelectronic devices to monitor enzymatic reactions, offering significant advantages in terms of speed and cost compared with conventional analytical methods."*

Reviewer #2 (Remarks to the Author):

General Comments: In the manuscript entitled "Real-Time Electrical Monitoring of Enzymatic Conformational Dynamics at the Single-Molecule Level", the authors employ a scanning tunneling microscope to investigate the enzymatic catalytic process through measurement of electrical conductance. Some major comments and amendments that need to be addressed are listed below:

Response: We thank the reviewer for the careful evaluation of our manuscript and for the constructive and helpful comments. We have revised the manuscript accordingly and addressed each point in a comprehensive, point-by-point manner below. We hope that these revisions have substantially improved the clarity, rigor, and overall quality of the work.

1. The authors assert that enzymatic conformational dynamics during the catalytic process can be probed and monitored by utilizing a scanning tunneling microscope (STM) to measure electrical conductance. The author expressed incorrect concepts in the title, abstract, and results section that monitoring of enzymatic conformational dynamics through electrical signal. The authors have merely identified electrical signal

variations during the P450 enzyme catalytic process, possibly indicating changes in electron transfer pathways or mechanisms. At present, there exists no theoretical or experimental evidence directly linking conductance changes with conformational dynamics. In contrast, established methods such as NMR, CD, X-ray crystallography, and Cryo-EM are well-validated techniques for studying conformational changes. Therefore, the author needs to revise these parts of the content.

Response: We thank the reviewer for these critical and constructive comments. We fully agree that establishing a rigorous link between single-molecule conductance variations and protein conformational dynamics is essential. To address this concern and provide both experimental and theoretical support for this correlation, we have performed the following additional studies and revised the manuscript accordingly.

(1) Experimental evidence: direct correlation via CD spectroscopy

To bridge the gap between electrical signals and conformational changes, we conducted systematic CD spectroscopy measurements on CYP1A1 across its complete functional cycle (Fig. R3, also Fig. 2 in the main text), including the apo enzyme, holo enzyme, and complexes with cofactors (NADH/NADPH) and at varying concentrations of substrate (BaP). Quantitative analysis of the secondary structures reveals that the α-helical content increases upon binding of reduced cofactors (Figs. R3a, b), whereas BaP binding induces a concentration-dependent decrease in α-helical content (Figs. R3d, e). Importantly, we observed a strong linear and positive correlation between the α-helical content and the conductance of Peak II and Peak III (Figs. R3c and R3f), while β-sheet and random coil contents exhibit a clear negative correlation with conductance (Figs. S21 and S25, newly added in Supporting Information). Notably, a similar dependence of conductance on α-helical content has been reported for bovine serum albumin (BSA), where ligand binding reduced α-helical content and led to a corresponding decrease in conductance (*Materials* 12, 1022 (2019)).

These observations can be rationalized by crystallographic studies of CYP1A1, which show that the active-site pocket is primarily framed by the F and I helices (*J. Biol. Chem.* 282, 14348 (2007) and *Biochem.* 59, 2012-2021 (2020)). These helices host

dense networks of aromatic residues, such as Tyr, Trp, and Phe, which are known to facilitate electron tunneling (*Chem. Commun.* 61, 11538-11550 (2025)). CD spectroscopy indicates that BaP binding induces partial unwinding or rearrangement of the F and I helices to accommodate the bulky substrate. Such conformational rearrangements are expected to alter the intermolecular distances and change the alignment between these aromatic "stepping stones," thereby increasing the tunneling barrier. Together, these results provide direct experimental evidence that the measured conductance is modulated by conformational changes of the enzyme during the catalytic cycle.

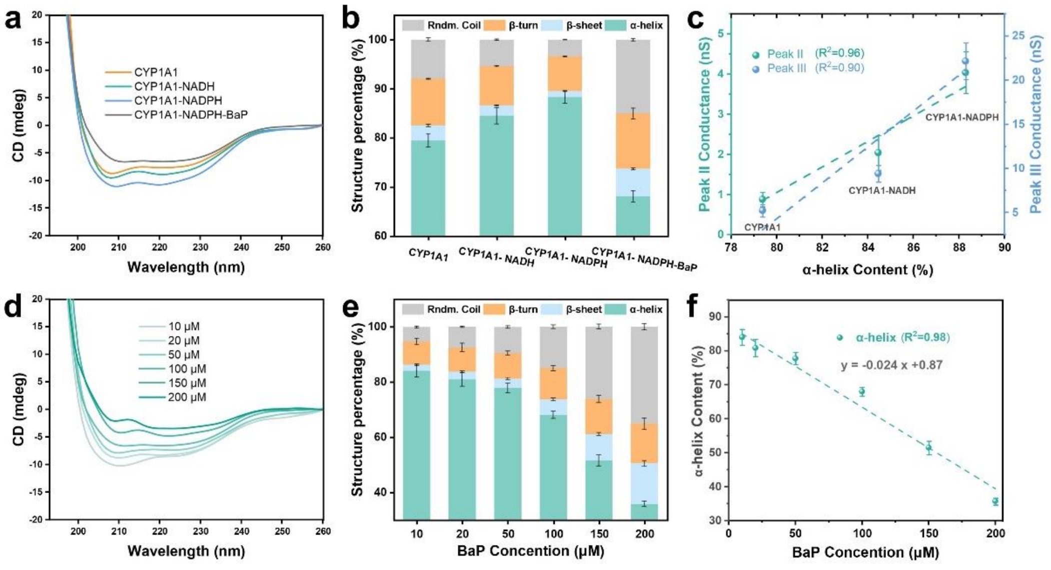

Fig. R3 Relationship between protein secondary structure and conductance under different catalytic conditions. (a, b) CD spectra and corresponding secondary-structure contents of CYP1A1, CYP1A1-NADH, CYP1A1-NADPH, and CYP1A1-NADPH-BaP. (c) Correlation between α-helical content and measured conductance. (d, e) CD spectra and secondary-structure analysis of CYP1A1-NADPH at different BaP concentrations. (f) Dependence of α-helical content on BaP concentration. Data are presented as mean ± s.d. (n = 3).

(2) Theoretical support: calculation of electronic structures

In addition to conformational effects, cofactor binding also reduces the heme iron from Fe^{3+} to Fe^{2+}. To elucidate how this redox process modulates the tunneling pathway at the electronic level, as suggested by the reviewer in Q13, we performed QM/MM calculations on the active center. The results show that reduction of the heme iron

narrows the HOMO-LUMO gap and shifts the heme orbitals into closer energetic alignment with the Fermi level of the gold electrodes and with the redox potential levels of nearby aromatic residues located within the α-helical scaffold (Fig. R4a and Fig. S22 in the SI). This redox-induced realignment enhances electronic coupling and facilitates charge injection through the metal center, providing a quantum-chemical rationale for the higher conductance. Consistently, NEGF-DFT transport calculations yield a substantially higher transmission near the Fermi level for the Fe^{2+} state than for Fe^{3+} (Fig. R4b), in agreement with the experimentally observed conductance enhancement.

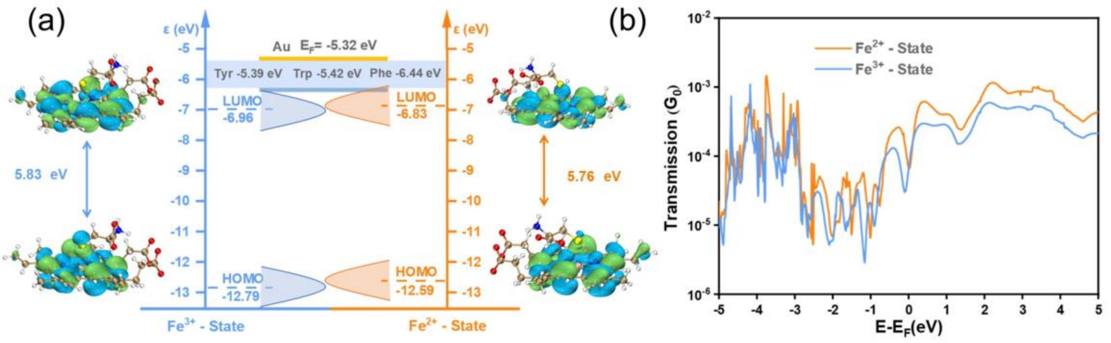

Fig. R4 Calculation of HOMO-LUMO Energy Levels and Transmission Spectra for Different Redox States in the Active Center of CYP1A1. (a) HOMO-LUMO energy levels of the heme iron in ferric (Fe^{3+}) and ferrous (Fe^{2+}) states, calculated within a protein-solvent environment, relative to the Fermi level of Au (EF = -5.32 eV). (b) The transmission function of the CYP1A1 active center in the Fe^{2+} and Fe^{3+} states.

In response to the reviewer's suggestion for improved accuracy, we have refined the presentation of our findings. The title of the manuscript has been revised to "*Real-Time Electrical Monitoring of Enzymatic Catalytic Dynamics at the Single-Molecule Level*" to clarify that catalytic processes are probed through electrical readouts. Throughout the Abstract, Results, and Conclusions, we now explicitly state that protein conductance correlates with and reflects conformational transitions, rather than implying direct structural visualization. We hope that these revisions adequately address the reviewer's concerns regarding the scientific rigor of our interpretation. The manuscript has been extensively updated to incorporate the experimental results and discussion. (Pages 11-15, lines 254-360).

2. “The authors assign Peak I (~0.22 nS)—commonly observed in other proteins such as antibodies and DNA polymerase—to an asymmetric junction configuration involving a specific contact (e.g., an Au–S bond at the substrate) and a nonspecific contact at the probe.” What is the basis for this assignment? Is it the conductance value, peak shape, or another criterion?

Response: We thank the reviewer for this question. The assignment of peak I (~0.22 nS) to an asymmetric junction configuration, comprising one specific covalent contact and one non-specific physical contact, is primarily based on its characteristic conductance value. In our previous study on non-redox proteins (*Proc. Natl. Acad. Sci.* 116, 5886-5891, (2019) and *ACS Nano* 14, 1360-1368(2020)), we demonstrated that conductance values in the range of 0.2-0.3 nS are a characteristic feature of such asymmetric contact geometries, clearly distinct from the higher conductance values (>1 nS) observed for symmetrically contacted configurations.

To further substantiate this assignment for the CYP1A1 system, we performed an additional control experiment in which the STM tip was functionalized with mercaptoethanol (MCE) to block specific thiol-gold interactions. As shown in Fig. R5 (also Fig. S13 in the SI), the higher-conductance features (Peaks II and III) completely disappeared, whereas Peak I (~0.25 nS) remained. This result demonstrates that Peak I does not require a specific chemical bond (e.g., via a surface-exposed cysteine) at the probe and instead arises from a non-specific physical contact between the tip and the protein.

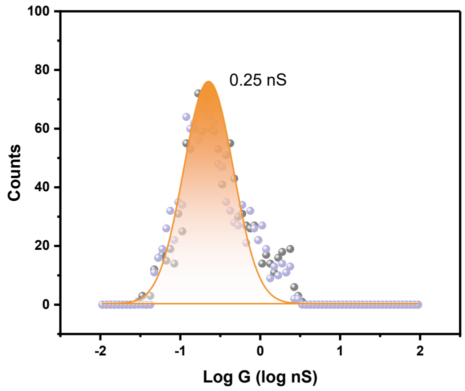

Fig. R5 (also Fig. S13 in the revised Supporting Information). Protein conductance measured with MCE-passivated Au tips showed only Peak I (Peaks II and III absent),

with triplicate values of 0.23, 0.25, and 0.26 nS.

We thank the reviewer again for pointing out this ambiguity. The results of this control experiment and the corresponding clarification have now been incorporated into the revised manuscript. (Page 8, lines 170-179).

"Our previous work demonstrated that reliable protein conductance measurements require at least one strongly coupled electrode-protein contact, formed either via covalent bonding or ligand-mediated interactions[22]. To evaluate the role of contact chemistry, the STM probe was passivated with mercaptoethanol (MCE) to suppress the formation of specific Au-S bonds. Under these conditions, Peaks II and III disappeared from conductance histogram (Fig. S13), whereas Peak I (0.25 nS) persisted. This observation indicates that Peak I originates from asymmetric junction configurations involving one specific contact (e.g., Au-S bond at the substrate) and one non-specific contact at the probe, a configuration previously observed in other protein junctions, such as antibodies and DNA polymerase[22,27]."

3. "It is suggested that the higher conductance Peaks II and III may originate from two specific contacts, given the two surface-exposed cysteine residues (Cys20 and Cys502, Fig. S13) in CYP1A1." To which specific states or conformations do these two conductance values correspond? Why is there such a significant difference between the conductance values attached to these two cysteines, and how would the conductance change if these cysteines were mutated to residues (such as Ala), which are unable to form specific contacts?

Response: We thank the reviewer for this insightful question regarding the assignment of the conductance peaks. We apologize for any confusion arising from insufficient clarity in the previous version of the manuscript. In essence, the formation of specific chemical contacts through surface-exposed cysteine residues is a prerequisite for the appearance of both Peak II and Peak III, whereas the pronounced difference in their conductance values originates from two distinct internal charge-transport pathways, namely a protein matrix-mediated pathway and a metal-mediated pathway. We

appreciate the opportunity to clarify the respective roles of electrode contacts and charge-transport mechanisms underlying the observed signals.

We first attribute the emergence of both the high-conductance states (Peaks II and III) to the formation of specific chemical contacts, i.e., Au-S covalent bonds, between the gold electrodes and the surface-exposed cysteine residues Cys20 and Cys502. To verify this assignment, we performed a control experiment in which the STM tip was passivated with MCE, thereby suppressing the formation of thiol-gold bonds. Under these conditions, both Peaks II and III disappeared completely, leaving only the low-conductance Peak I (~0.25 nS; Fig. R5). This result demonstrates that the higher-conductance features critically depend on the establishment of specific chemical contacts. We appreciate the reviewer's suggestion to perform Cys-to-Ala mutagenesis; however, we note that point mutations in a complex metalloenzyme such as CYP1A1 may inadvertently induce structural collapse or misfolding, complicating the interpretation of the conductance data. In this context, tip passivation may provide a more reliable and noninvasive control, as also demonstrated in our previous work (*Nano Lett.* 19, 4017-4022 (2019))

Importantly, we clarify that Peaks II and III do not arise from binding to different cysteine residues (i.e., Cys20 versus Cys502), but instead correspond to two distinct internal charge-transport pathways once specific electrode contacts are established. As shown by experiments using iron-depleted apo-CYP1A1, Peak III vanishes entirely (Fig. 1h). Accordingly, Peak III (4.47 nS) is assigned to a metal-mediated transport pathway that critically depends on the presence of the heme iron. In contrast, Peak II (0.93 nS) persists in the apo-enzyme and is therefore attributed to a protein-matrix pathway, in which charge transport occurs through the peptide backbone and networks of aromatic residues. The coexistence of these two transport channels is consistent with previous observations in other metalloproteins, such as azurin, reported by David and co-workers (*Proc. Natl. Acad. Sci.* 121, e2405156121 (2024)). The approximately fivefold difference in conductance between Peaks II and III thus primarily reflects the

higher efficiency of redox-assisted transport through the heme relative to matrix-based tunneling.

Regarding whether attachment at Cys20 and Cys502 would yield resolvable conductance differences, our analysis suggests this may be unlikely. Although binding at different cysteine sites may alter the effective transport length, our previous studies have shown that protein conductance in systems with two specific chemical contacts exhibited only a weak dependence on molecular length once strong chemical contacts are established (*Nano Lett.* 19, 4017-4022 (2019)). Moreover, given the broad distribution of peaks in I-V histograms (Fig. 1), corresponding to limited conductance resolution of the technique, such minor geometric differences are not expected to be resolvable.

In summary, Peak III reports on a metal-mediated transport pathway, whereas Peak II reflects a protein-matrix pathway; the formation of both states requires specific cysteine-gold contacts via either Cys20 or Cys502. The pronounced conductance difference between the two peaks originates from the distinct charge-transport efficiencies of these pathways. We have incorporated this clarification into the revised manuscript and hope that it satisfactorily addresses the reviewer's concerns. (Pages 8, lines 170-192).

4. In the case of apo-CYP1A1, Peak III disappears, which the authors attribute to the absence of the ferric ion that mediates an efficient charge transport pathway in the holoenzyme. However, if both Peaks II and III indeed arise from specific contacts at Cys20 and Cys502, why does Peak II not just persist but also exhibit a slight rise in conductance?

Response: We thank the reviewer for this insightful observation. As demonstrated in our study, Peak III is assigned to a metal-mediated transport pathway, whereas Peak II corresponds to a protein-matrix pathway, as confirmed by conductance measurements of the apo-enzyme in the absence of the heme metal center.

The slight shift of Peak II toward higher conductance in apo-CYP1A1 can be rationalized by structural rearrangements of the protein scaffold upon removal of the bulky, centrally located heme prosthetic group. This interpretation is supported by our newly acquired circular dichroism (CD) data, which reveal that the transition from the holo- to the apo-enzyme is accompanied by significant changes in secondary structures (Fig. R6, also Fig. S20 in the SI). Such conformational reorganization is expected to alter the packing and electronic coupling of the peptide backbone and aromatic residues that constitute the matrix transport pathway (Peak II), thereby modulating its tunneling efficiency and accounting for the observed increase in conductance.

We hope that this explanation addresses the reviewer's question. The corresponding clarification has been incorporated into the revised manuscript to explain the conductance shift observed in apo-CYP1A1. It reads as follows:

"Moreover, compared with holo-CYP1A1, the conductance of Peak II increases from 0.93 nS to 1.59 nS upon removal of the metal center. This enhancement likely reflects structural distortion or altered secondary-structure organization induced by heme extraction, as further evidenced by CD spectroscopy discussed below." (Page 10, lines 210-214).

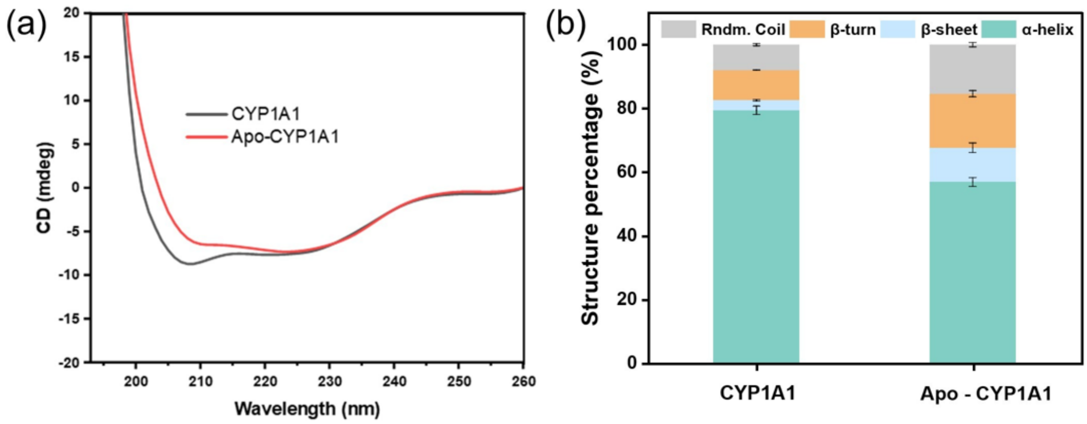

Fig. R6 Comparison of CD spectra and secondary structures between CYP1A1 and Apo-CYP1A1 (newly added Supplementary Fig. S20 in supporting information). (a) CD spectra of CYP1A1 and Apo-CYP1A1. (b) Changes in secondary structure content of CYP1A1 and Apo-CYP1A1.

5. After the addition of the coenzyme NADPH, three conductance peaks remain, all showing increased values compared to those of the enzyme alone. If NADPH was introduced during the measurement, binding would require some time to reach equilibrium. If it was pre-incubated, the system should be at binding equilibrium. Which of the three peaks corresponds to the enzyme–NADPH complex in the bound state?

Response: Thank you for raising this question regarding the equilibrium state and the assignment of the conductance peaks in the presence of the coenzyme. We appreciate the opportunity to clarify this point. In our experiments, the enzyme was pre-incubated and measured in a buffer containing 1 mM NADPH, a concentration well above its dissociation constant, thereby ensuring that the binding equilibrium is established and the enzyme is fully saturated with the coenzyme.

Under this condition, all three peaks in the CYP1A1-NADPH conductance histogram arise from the enzyme-NADPH complex. The addition of NADPH induces two major effects: conformational changes in the protein and reduction of the heme center to the Fe^{2+} state. The former is supported by newly performed circular dichroism measurements (Figs. R3a, b), which show a significant increase in α-helical content upon NADPH binding; this structural change correlates positively with the conductance enhancement (Fig. R3c). The latter effect was examined by electronic structure calculations of the active center via QM/MM and DFT (Fig. R4), which indicate that reduction of the heme iron narrows the HOMO-LUMO gap and improves energetic alignment of the LUMO with the Fermi level of the gold electrodes. We therefore conclude that these two effects act synergistically to strengthen electronic coupling and charge transmission through both the metal-mediated pathway (Peak III) and the protein-matrix pathway (Peak II), leading to an overall shift of all conductance peaks toward higher values in the statistical histogram.

We have revised the manuscript to explicitly clarify the conductance signatures of the enzyme-NADPH complex and the origin of the observed peak shifts. We hope that

this explanation, together with the updated discussion, adequately addresses the reviewer's concern.

"The influence of coenzymes on protein conformation was further evaluated by introducing 1 mM NADPH or NADH into the buffer. Both coenzymes induced more negative ellipticity at α-helix–characteristic wavelengths, indicative of conformational rearrangements upon coenzyme binding. Quantitative secondary-structure analysis reveals that the α-helical content increases by ~9% in the presence of NADPH (Fig. 2b), suggesting conformational stabilization of CYP1A1 and a transition toward a more ordered, catalytically competent state[35]." (Pages 11-12, lines 264-270)

"In a canonical CYP1A1 catalytic cycle, NADPH binding not only increases the α-helical content but also drives reduction of the heme iron from the ferric (Fe^{3+}) to the ferrous (Fe^{2+}) state, thereby enabling molecular oxygen binding and initiating substrate oxidation (Fig. 3a)[41]." (Page 14, lines 322-325)

"As shown in Fig. 3b, the Fe^{2+} state exhibits a significantly higher transmission coefficient near the Fermi energy than the Fe^{3+} state, providing theoretical support for the enhanced conductance observed experimentally in the reduced enzyme." (Page 15, lines 351-353)

6. Upon addition of the substrate BaP, three peaks are still observable. Given that both the enzyme and the enzyme–NADPH complex each exhibit three characteristic conductance peaks, why do three new peaks, which differ from the conductance value before, appear during the reaction? Does this imply that the free enzyme and the enzyme–NADPH conformations are no longer present during the catalytic process?

Response: The reviewer has raised an important question regarding the interpretation of the conductance signals after substrate addition, and we appreciate the opportunity to clarify this point. It appears that certain aspects of the measurement principle may not have been sufficiently clarified in the previous version of the manuscript, and we thank the reviewer for drawing our attention to this issue.

First, we would like to emphasize that the I-V characterization of protein conductance is inherently statistical in nature. In each I-V sweep, the STM tip randomly captures a single enzyme molecule, and more than one thousand such traces are collected to construct a conductance histogram. The most probable conductance values, which correspond to the dominant electron-transport pathways, are then extracted from this statistical distribution. Similar ensemble-based approaches are widely used in single-molecule electronics, such as STM break-junction measurements (*Science* 301,1221-1223(2003)). As shown in Fig. 1, even when plotted on a logarithmic scale, the conductance peaks are still relatively broad, reflecting significant molecule-to-molecule and junction-to-junction variability. Consequently, the intrinsic resolution of this technique for discriminating closely spaced conductance states is limited, which necessitates a histogram-based, ensemble-averaged analysis rather than the assignment of individual I-V traces to discrete conductance states.

After the addition of BaP, the catalytic cycle is initiated, and in principle three species may coexist: the free enzyme, the enzyme-NADPH complex, and the enzyme-NADPH-BaP ternary (ES) complex. However, their populations and, correspondingly, their probabilities of being captured by the STM tip are not equal. Since NADPH is present at a high concentration (1 mM), the population of free enzyme is expected to be negligible, and junction formation is dominated by either the enzyme-NADPH complex or the ES complex. The fraction of the ES complex increases with increasing BaP concentration. As a result, for a given substrate concentration, the ~1000 I-V traces used to construct the histogram represent a mixture of these two bound states. Given the limited conductance resolution of the I-V histogram, the conductance features of the enzyme-NADPH complex and the ES complex are unlikely to be resolved as separate, well-defined peaks. Instead of yielding six distinct peaks, their contributions merge into a single trimodal distribution, with substrate binding manifested as systematic shifts in the peak positions. These conductance shifts, therefore, report on the changing population of the ES complex in a BaP-concentration-dependent manner, rather than on the appearance of entirely new, resolvable conductance states.

In summary, the absence of additional peaks upon BaP addition arises from the intrinsic breadth of the conductance distributions and the limited ability of the statistical STM approach to resolve small differences between closely related bound states. For this reason, peak shifts, rather than peak multiplicity, were used to quantify the relative population of the ES complex. We hope that this clarification adequately addresses the reviewer's concerns.

7. There is a clear discrepancy between the conductance test results presented earlier and the subsequent high-resolution current–time (i–t) measurements. Using a "molecular fishing" strategy, the authors identify four states (S0 to S3). How exactly are these four conductance states related to specific stages in the catalytic cycle? Simple logic-based or exclusion-based assignment seems insufficient, as conductance variations can surface from multiple sources.

Response: We thank the reviewer for this question. We employed the *i-t* measurement to monitor the catalytic dynamics of CYP1A1 during BaP metabolism because of its high temporal resolution. We would like to clarify that the I-V and *i-t* techniques probe different dimensions of the single-molecule junction. The conductance histogram obtained from I-V sweeps represents an ensemble-averaged distribution of conductance values and thus reveals the most probable electron-transport pathways through the enzyme. In contrast, each *i-t* trace is recorded from a single, stable junction configuration, and the superimposed telegraph-noise (TN) fluctuations reflect the real-time temporal evolution of the catalytic process. Such dynamic information cannot be readily accessed by I-V measurements owing to their limited temporal resolution.

In the presence of BaP, four distinct TN states (S0-S3) were resolved in the *i-t* measurements. We would like to emphasize that the assignment of these states is not based on logical exclusion but on dedicated control experiments combined with quantitative statistical analysis. Mass spectrometry (Fig. S29) identified two major reaction intermediates, 7-OH-BaP and 7,8-dihydrodiol-BaP. When these compounds were individually introduced as substrates, the corresponding *i-t* traces exhibited well-

defined two-state TN signals with characteristic relative current changes. The amplitudes of these changes (approximately 17% and 46%) coincide with the S0→S1 and S0→S2 transitions observed during complete BaP turnover (Figs. 5a-d), providing electronic "fingerprints" that allow S1 to be assigned to the phenolic product (7-OH-BaP) and S2 to the dihydrodiol intermediate (7,8-D-BaP). The S3 state, characterized by a larger conductance decrease (~76%), is assigned to the highly reactive epoxide intermediate 7,8-epoxy-BaP. This assignment is supported by hidden Markov modeling and dwell-time analysis, which show that S3 has the longest mean lifetime among all states (Fig. 5f-g), consistent with the epoxidation step being rate-limiting in the CYP1A1-catalyzed BaP activation pathway. In addition, the newly added transition-density heatmap analysis (Fig. S28) reveals that S0 most frequently transitions to S3 and S1, in agreement with the known initial branching of the metabolic pathway (*Chem. Res. Toxicol.* 29, 1325-1334 (2016)).

We hope that these clarifications address the reviewer's concerns. The manuscript has been revised to expand this discussion and to more explicitly articulate the experimental basis for the state assignments. It reads as follows:

"To elucidate the transition pathways among these states, we performed a statistical analysis of state-to-state transitions using more than 1,500 events collected from ~50 measurements. The resulting transition-density heatmap (Fig. S28) reveals a clear kinetic pattern: transitions originate predominantly from the baseline state S0 and bifurcate into either S1 or S3. Transitions between S2 and S3, as well as between S1 and S3, are also prominent, whereas transitions such as S0→S2 and S1→S2 occur only rarely. This transition pattern likely reflects distinct catalytic steps during BaP turnover." (Page 20, lines 478-485)

"Using these two intermediates, we successfully identified the S1 and S2 conductance states based on the relative amplitude characteristics of the TN signals. However, the S3 state could not be directly assigned due to the absence of a stable, isolatable intermediate. Based on the established BaP metabolic sequence, (+)-BaP-7,8-epoxide (7,8-E-BaP), the precursor of 7,8-D-BaP[47], is the most likely candidate for

the S3 state. However, 7,8-E-BaP is unstable, prone to hydrolysis, and therefore unsuitable for direct state assignment. Nevertheless, it is reasonable to associate S3 with the epoxidation step of BaP catalysis. Aligning these results with the canonical CYP1A1-mediated BaP metabolic pathway, we propose the following correspondence between conductance states and catalytic intermediates: S0 (initial enzyme-substrate complex)→S3 (7,8-E-BaP)→S2 (7,8-D-BaP) and S0→S1 (7-OH-BaP). These two distinct metabolism pathways are consistent with the observed transition patterns among the conductance states (Fig. S28).” (Page 23, lines 533-545)

8. In the section discussing substrate concentration dependence of CYP1A1 conductance, the model mistakenly turns a data processing problem into a scientific problem. The generation of ΔG due to the different ratio of enzyme molecules in the ES state and the original state. But the experiment is performed in the reaction state, thus the identification of the different states' curves with the clustering algorithm is essential and possible to achieve, according to Fig.2b.

Response: We appreciate the reviewer’s constructive suggestion. We agree that, if a reliable clustering algorithm could be developed to classify I-V curves corresponding to different enzymatic states, this would, in principle, provide a more direct route to extracting the kinetic parameters of CYP1A1 catalysis. However, in practice, using conductance alone as the classification criterion presents challenges for two main reasons. First, as discussed in Q6, the I-V technique is inherently statistical. For an individual I-V trace, the conductance obtained from the slope can vary significantly, as reflected by the broad distributions in the conductance histograms (Fig. 1). Second, the enzyme captured by the STM probe does not populate a simple two-state system, but rather exists in a dynamic steady state comprising multiple intermediates (ES, S1, S2, and S3). Consequently, these states exhibit partially overlapping conductance values, making it challenging to unambiguously classify them solely on the basis of conductance without invoking more sophisticated algorithms that exploit additional features of the I-V curves.

Since these enzymatic states have overlapping conductance signatures and interconvert rapidly, the shift in the ensemble-averaged peak position (ΔG) provides a more robust and reproducible metric that reflects the population-weighted average of all active species within the junction. This approach is conceptually analogous to conventional bulk kinetic assays (*Anal. Chem.* 98, 1503-1513 (2026).), in which global observables, such as total product formation or cofactor consumption, are used as integrated readouts of the entire catalytic cycle rather than of individual intermediates. In our system, the conductance shift relative to that of the enzyme-NADPH complex serves as a sensitive indicator of the population of the ES state, i.e., the fraction of enzymes with an occupied active site. Importantly, the resulting K_m values (24.2-43.2 μM) are in close agreement with reported literature values for free CYP1A1, supporting that ΔG reliably captures the underlying substrate binding affinity and catalytic kinetics.

We thank the reviewer again for this valuable suggestion. We will explore in future work whether advanced data-analysis approaches, such as machine-learning-based pattern recognition, could be applied to classify I-V traces according to distinct enzymatic states. We hope that the above explanation satisfactorily addresses the reviewer's concern.

9. After the identification of the different states' curves, Fig.3d can be corrected. Now it looks like the logarithmic relationship rather than the linear one. Maybe there are other influencing factors for the reaction not modeled by Michaelis-Menten kinetics?

Response: We appreciate the reviewer's careful observation regarding the linearity of the Lineweaver-Burk plot. We agree that there is slight deviation from perfect linearity, indicating that the system is not fully described by the ideal Michaelis-Menten model. This deviation likely arises from the fact that our measurements are performed on an immobilized enzyme within a single-molecule junction, where the overall reaction rate is governed by a combination of intrinsic catalytic kinetics and mass transport of substrate to a spatially confined active site. Such mixed kinetic-diffusion control is well

documented for surface-bound enzymes and is known to cause deviations from ideal saturation behavior (*Anal. Chem.* 89, 10311-10320 (2017)).

To quantitatively evaluate the role of diffusion, we calculated the Damköhler number (*Da*; see Method) and obtained a value of approximately 0.23. This places our system in the mixed kinetic-diffusion regime (0.1 < Da < 10), rather than in the limits of purely kinetic-controlled (Da ≪ 0.1) or purely diffusion-controlled (Da ≫ 10) behavior. Under such conditions, the experimentally determined Michaelis constant is expected to be an apparent value, K_m^{app}, larger than the intrinsic kinetic constant by a factor of approximately (1 + Da) ≈ 1.23.

To be more accurate, we have revised the manuscript to consistently refer to the extracted parameter as the apparent Michaelis constant, K_m^{app}, and have added a discussion of mass-transport effects and Damköhler analysis to clarify the kinetic regime and ensure a rigorous interpretation. We thank the reviewer again for this important and helpful comment.

"This deviation likely reflects the heterogeneous nature of the conductance assay, in which the enzyme is immobilized on a gold surface. Under these conditions, the observed reaction rate is governed by both intrinsic catalytic kinetics and the mass transport of substrate to a spatially confined active site. To quantify the influence of diffusion,we calculated the Damköhler number (Da) and obtained a value of approximately 0.23 (Methods). This places the system in the mixed kinetic-diffusion regime (0.1 < Da < 10), rather than in the limits of purely kinetic-controlled (Da ≪ 0.1) or purely diffusion-controlled (Da ≫ 10) behavior. In this regime, the experimentally determined K_m^{app} *is expected to be larger than the intrinsic kinetic constant by a factor of approximately (1 + Da) ≈ 1.23. These results collectively demonstrate that the conductance response of CYP1A1 provide a sensitive and quantitative measure of enzymatic kinetics. This establishes a robust framework for employing protein-based bioelectronic devices to monitor enzymatic reactions, offering significant advantages in terms of speed and cost compared with conventional analytical methods."* (Pages 18, lines 435-449)

10. In Fig. 4f, the HMM and QuB software has been employed to idealize your experiment-based curves. How do you define your original transition model for the HMM estimation? In principle, QuB will only generate the segment types according to the number of transition states you define. But in Fig. 4f, the idealized curve shows more plateau types than four states, which is not aligned with the mechanism you proposed.

Response: We thank the reviewer for this careful observation and for pointing out the fitting issue in the HMM/QuB analysis. The model for HMM estimation was initialized with four discrete current levels extracted from the all-points current histograms in Figs. 5a-5c. We fully agree that, with the number of states fixed, the QuB algorithm should yield only the corresponding number of plateau levels. The appearance of additional minor plateaus beyond the four dominant ones was therefore most likely caused by inappropriate fitting parameters, particularly the current and dwell-time variances, which led to a fraction of the noise being misidentified as additional states.

We have now corrected the HMM/QuB analysis by optimizing these parameters, which results in a segmentation consistent with four well-defined states, as illustrated in Fig. R7 (also Fig. 5e in the revised manuscript). The manuscript has been updated accordingly. We thank the reviewer again for this important and helpful comment.

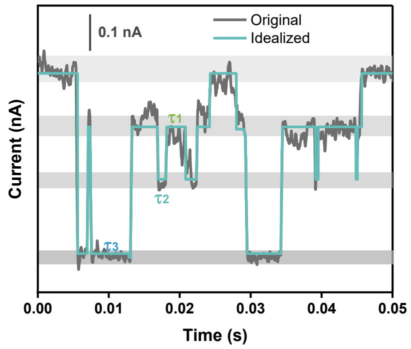

Fig. R7 (Updated as Fig. 5e in the revised Manuscript). Example *i-t* trace segment idealized using a segmental k-means algorithm with hidden Markov modeling, fitted using QUB software (green trace).

11. You use two kinds of I-V scan (Fig. 1e) and I-T records (Fig. 4abc). The first mode shows three peaks for each state, while the latter records one representative value per state. Could you clarify the relationship between these two modes and their outcomes? Only the Peak III in the I-V scan is recorded by the i-t curves? Why? And please use the same units (such as nS) in both tests, so that they can be compared.

Response: We thank the reviewer for requesting clarification of the relationship between the statistical I-V histograms and the real-time I-t measurements. These two recording modes probe complementary aspects of the single-molecule junction. The I-V histograms represent an ensemble-averaged distribution of conductance values and reveal three dominant electron-transport pathways through the enzyme: the metal-mediated pathway (Peak III), the protein-matrix pathway (Peak II), and the contact-resistance-dominated pathway (Peak I). The efficiencies of these pathways are modulated by both the redox state of the heme iron (upon cofactor binding) and conformational changes during catalysis. In contrast, the *i-t* traces monitor the temporal evolution of a single, stable junction configuration. Each trace consists of a steady DC component, corresponding to the baseline conductance of that junction, and superimposed telegraph-noise (TN) fluctuations that arise from stochastic switching between catalytic states and thus report directly on enzyme activity in real time (*ACS Nano* 2020, 14, 1360-1368). An intuitive analogy is that the baseline current reflects the dominant transport pathway of a given junction, whereas the TN signals capture dynamic conformational and chemical transitions that are necessarily averaged out in the ensemble I-V histograms.

For the *i-t* measurements, it is not necessary to select a baseline current corresponding specifically to Peak III. As shown in Fig. 1j, in the presence of BaP, both Peaks II and III shift to lower conductance values. Moreover, in the *i-t* measurements, we found that the TN amplitudes scale with the baseline current, with the relative current changes for states S1, S2, and S3 being approximately 14%, 51%, and 76% (Fig. 5d), respectively. Therefore, identification of the TN states relies on their relative amplitudes rather than on the absolute amplitudes. Nevertheless, since Peak III

corresponds to the metal-mediated pathway through the active center, it is expected to be more sensitive to catalytic events and thus to yield a higher signal-to-noise ratio in the *i-t* traces.

We also thank the reviewer for the suggestion to unify the conductance units to nS, and the manuscript has been revised accordingly. We hope this explanation clarifies the roles of I-V and *i-t* measurements and resolves the reviewer's concerns.

12. To make it more credible, each possible single state should be tested under the current–time (i–t) measurements.

Response: We thank the reviewer for this constructive suggestion. As suggested, we have performed *i-t* measurements under conditions corresponding to the enzyme's non-catalytic functional states, including the free enzyme (CYP1A1), the inactive metal-free apo form (Apo-CYP1A1), and the enzyme bound separately to different cofactors (CYP1A1-NADH and CYP1A1-NADPH).

The resulting traces (representative examples shown in Fig. R8) confirm that stable single-molecule junctions formed in all cases, as evidenced by steady baseline current. Importantly, no significant TN fluctuations are observed in any of these control experiments. This demonstrates that the TN dynamics observed in the presence of both NADPH and BaP are not a generic feature of junction formation or cofactor binding, but are specifically associated with active catalytic turnover of the substrate.

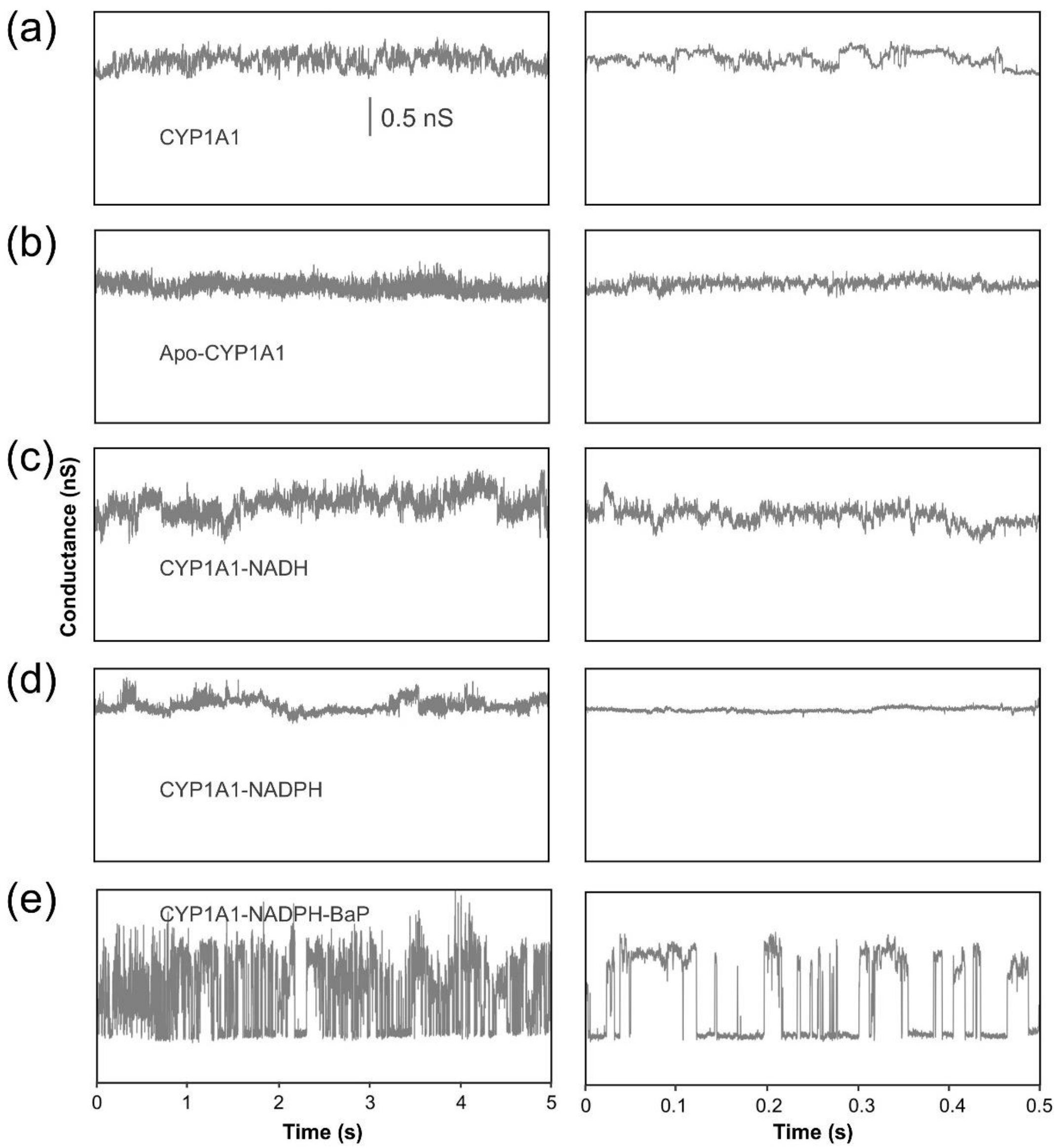

Fig. R8. Representative I-t traces of each possible single protein state (also Fig. S27 in the revised Supporting Information). (a) CYP1A1, (b) Apo-CYP1A1, (c) CYP1A1-NADH, (d) CYP1A1-NADPH, and (e) CYP1A1-NADPH-BaP. In the left panel, the 5-second *i-t* trajectories are presented. The right panel displays representative 0.5-second expanded sections randomly chosen from each trajectory.

We thank the reviewer again for this helpful suggestion. The corresponding data and discussion have been added to the manuscript to strengthen the TN signal interpretation.

"A representative TN trace is shown in Fig. 5a and Fig. S26. Notably, TN signals appeared only in the presence of both NADPH and BaP. Control experiments conducted

under non-enzymatic conditions showed no significant TN activity, confirming the catalysis-dependent origin of the observed fluctuations (Fig. S27).” (Page 20, lines 467-471).

13. The electronic structure of hemoglobin was calculated using DFT, but the model only included porphyrin and cysteine ligands, thus overlooking protein environment and solvent effects. This omission could compromise energy level accuracy and requires amelioration using the QM/MM method.

Response: We sincerely thank the reviewer for this critical comment on the limitation of the original electronic structure calculation. We agree that the electrostatic influence of the protein scaffold and solvent polarization is essential for a rigorous calculation of the electronic energy levels.

As suggested by the reviewer, we have replaced the model with an electrostatically embedded QM/MM framework implemented in ORCA 6.0.1. The revised calculations include a point-charge field derived from the AMBER force field to represent the electrostatic potential of the full protein environment and employ a conductor-like polarizable continuum model (CPCM) to account for solvent effects. After systematic screening of spin multiplicities, we adopted the ωB97M-V functional together with the def2-TZVP basis set to resolve the molecular orbital distributions under near-physiological conditions (See Method in the revised manuscript).

The results from this upgraded model confirm that the reduction of the heme center from Fe^{3+} to Fe^{2+} upon cofactor binding not only narrows the HOMO-LUMO gap but also shifts the LUMO closer to the Fermi level of the gold electrode and the redox potential of nearby aromatic residues (e.g., Tyr, Phe, and Trp, Fig. R4a and Fig. S22). In addition, quantum transport calculations based on the non-equilibrium Green’s function formalism combined with DFT reveal a markedly higher transmission near the Fermi level for the Fe^{2+} state compared with Fe^{3+} (Fig. R4b), in agreement with the experimentally observed increase in electron transport efficiency.

These methodological refinements and the corresponding results have been

incorporated into the revised manuscript. We hope that this addresses the reviewer's concerns regarding the rigor of the electronic structure analysis.

"A hybrid quantum mechanics/molecular mechanics (QM/MM) framework with electrostatic embedding was employed to account for protein environmental effects and solvent contributions (Methods). The model comprised the porphyrin macrocycle and the axial cysteine ligand coordinated to the iron center. As shown in Fig. 3c, in the resting ferric state, the lowest unoccupied molecular orbital (LUMO) is primarily localized on the porphyrin ring at -6.96 eV, whereas the highest occupied molecular orbital (HOMO) is distributed over the porphyrin and cysteine ligand at -12.79 eV. Reduction of the iron center to the ferrous state results in higher orbital delocalization, with the LUMO and HOMO shifting upward to -6.83 eV and -12.59 eV, respectively. Consequently, the HOMO-LUMO gap narrows from 5.83 eV to 5.76 eV. When considering a protein junction within gold electrodes, the elevated LUMO level in the ferrous state brings it closer to the Fermi level of the electrode (-5.32 eV)[42], as well as the redox energy levels of nearby aromatic residues (Tyr, -5.39 eV; Trp, -5.42 eV; Phe, -6.44 eV) located within ~2 nm of the active center (Fig. S22)[43]. This improved energetic alignment, combined with the reduced HOMO-LUMO gap, implies that the reduced state of CYP1A1 is inherently more conductive[26]. " (Page 14, lines 329-345).

Reviewer #3 (Remarks to the Author):

General Comments: The manuscript reports single-molecule electrical measurements of CYP1A1 during benzo[a]pyrene metabolism, identifying several conductance states that are assigned to catalytic intermediates. The experimental work is technically adequate and addresses an important challenge in enzymology. The results are interesting, and, in my view, the manuscript could be suitable for publication in Nature Communications. However, several aspects require clarification or further discussion before acceptance.

Response: We are grateful to the reviewer for the insightful comments and constructive suggestions. We have thoroughly evaluated each point, performed supplementary

experiments as appropriate, and revised the manuscript to fully address the reviewer's concerns. Our detailed responses are presented below.

1. My main concern is that the experimental observations are not sufficiently supported by a microscopic interpretation. The manuscript detects conductance changes and associates them with structural rearrangements, but no structural or computational analysis is provided to support such assignments. The work would benefit from a clearer mechanistic rationale explaining how the different conductance states relate to plausible conformational changes in CYP1A1, and from stronger connections with previous mechanistic or computational studies on P450 enzymes. At present, the interpretation is largely based on correlations, without independent evidence to validate the proposed sequence of states.

Response: We sincerely thank the reviewer for the constructive comments. We fully agree that establishing a rigorous mechanistic link between the measured electrical signals and specific conformational changes is essential for the scientific integrity of this work. To address this point, we have carried out comprehensive circular dichroism (CD) spectroscopy of CYP1A1 under different functional states, quantitatively analyzed the associated secondary-structure changes and their correlation with conductance, and complemented these experiments with refined electrostatically embedded QM/MM calculations to provide a physical basis for the observations. The details are provided below.

To bridge the gap between conductance signals and protein structures, we performed systematic CD measurements over the full functional cycle of the enzyme (Fig. R9), including the apo form, the holo form, and complexes with cofactors (NADH/NADPH) and with increasing concentrations of BaP. Quantitative analysis reveals a strong and positive correlation between α-helical content and the measured conductance (Fig. R9c,f). Crystallographic studies show that the active-site pocket of CYP1A1 is framed by the F and I helices (*J. Biol. Chem.* 282, 14348 (2007)), which host dense networks of aromatic residues (e.g., Tyr, Trp, and Phe) that are known to facilitate electron

tunneling (*ACS Phys. Chem. Au*, 3, 444-455 (2023) and *Proc. Natl. Acad. Sci.*121, e2405156121 (2024)). Our CD results indicate that NADPH binding stabilizes a more ordered, catalytically competent conformation with increased α-helicity, whereas binding of the bulky substrate BaP induces partial unwinding or rearrangement of these helices to accommodate the ligand. Such induced-fit remodeling, widely reported for cytochrome P450 enzymes (*Mol. Pharmacol.* 104, 154-163 (2023)), is expected to increase the tunnelling barrier within the protein matrix, providing a structural explanation for the conductance decrease upon substrate entry.

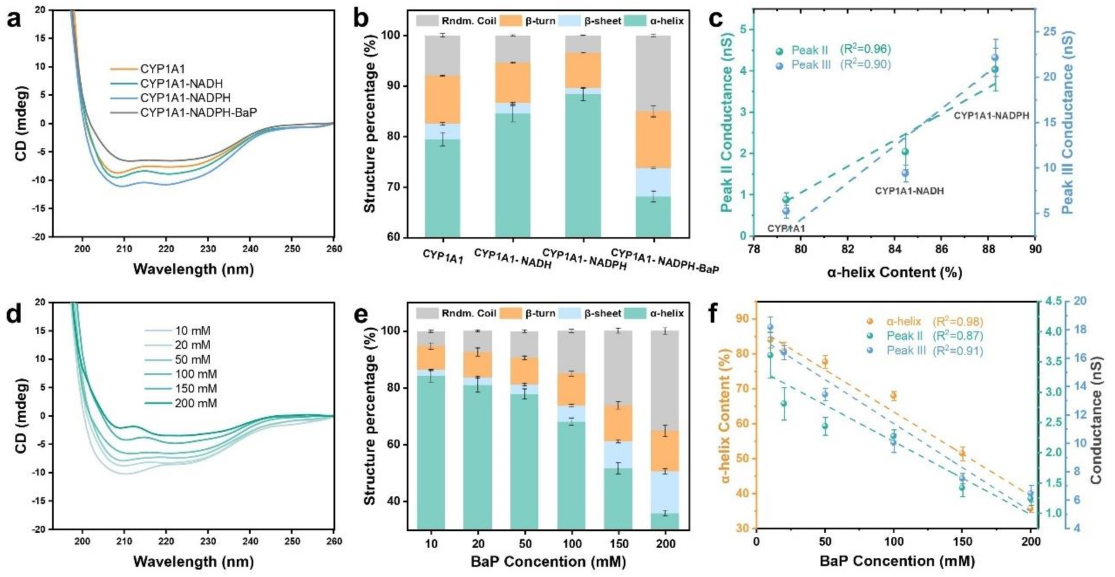

Fig. R9 Relationship between protein secondary structure and conductance under different catalytic conditions (newly added Fig. 2 in the revised manuscript). (a, b) CD spectra and corresponding secondary-structure contents of CYP1A1, CYP1A1-NADH, CYP1A1-NADPH, and CYP1A1-NADPH-BaP. (c) Correlation between α-helical content and measured conductance. (d, e) CD spectra and secondary-structure analysis of CYP1A1-NADPH at different BaP concentrations. (f) Dependence of α-helical content and conductance on BaP concentration. Data are presented as mean ± s.d. (n = 3).

Moreover, we employed an electrostatically embedded QM/MM framework to examine how the catalytic redox cycle modulates the electronic structure of the active site. The calculations demonstrate that reduction of the heme iron from Fe^{3+} to Fe^{2+} upon NADPH binding narrows the HOMO-LUMO gap and shifts the LUMO into closer energetic alignment with the Fermi level of the gold electrodes and with the redox potential levels of nearby aromatic residues located within the α-helical scaffold (Figs.

R10 a and Figs. S22). This redox-induced realignment enhances electronic coupling and facilitates charge injection through the metal center. Furthermore, newly performed NEGF-DFT transport calculations yield a substantially higher transmission near the Fermi level for the Fe^{2+} state than for Fe^{3+} sate, in agreement with the experimentally observed conductance enhancement (Fig. R10 b).

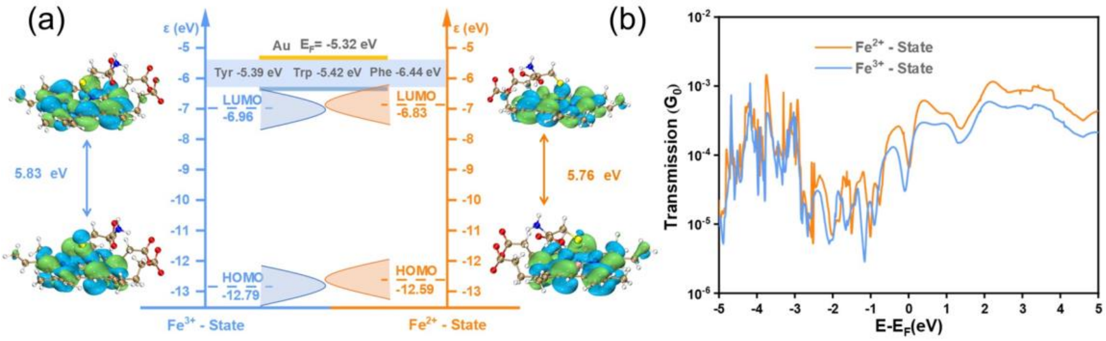

Fig. R10 Calculation of HOMO-LUMO Energy Levels and Transmission Spectra for Different Redox States in the Active Center of CYP1A1. (a) HOMO-LUMO energy levels of the heme iron in ferric (Fe^{3+}) and ferrous (Fe^{2+}) states, calculated within a protein-solvent environment, relative to the Fermi level of Au (EF = -5.32 eV). (b) The transmission function of the CYP1A1 active center in the Fe^{2+} and Fe^{3+} states.

Taken together, the revised manuscript now provides a multifaceted microscopic explanation where conformational changes (monitored by CD) alter the structural coupling of the protein matrix while catalytic redox transitions (analyzed by QM/MM) optimize the energy alignment for charge transport. We hope that this integrated experimental and theoretical analysis adequately addresses the reviewer's concern regarding the mechanistic origin of the conductance changes during enzymatic catalysis.

The relevant results and discussions have been incorporated into the revised manuscript. (Page 11, lines 254-316, and Page 14, lines 320-360)

2. There are also some internal inconsistencies. Peak III is first attributed to contact geometries involving surface cysteines (Cys20 and Cys502), later to Fe-mediated charge transport (based on the absence of Peak III in the apo enzyme), and later again to conformational changes around the active site. These explanations are not fully

compatible. The presence of the heme in the holo enzyme could itself induce conformational rearrangements that modify the protein–electrode contacts, which may explain the extra peak without invoking metal-mediated hopping. The discussion oscillates between "conformational effects" and "redox effects", but the causal separation between them is not clearly established.

Response: We apologize for any confusion arising from insufficient clarity in the previous version of the manuscript regarding the origin of Peak III. We fully agree with the reviewer that the interplay among contact geometry, redox state, and protein conformation is complex. In response to the reviewer's concern, we have performed additional control experiments and substantially expanded the discussion to provide a clearer and more rigorous interpretation of the assignment of Peak III, as explained below.

To first verify the role of electrical contact in establishing high conductance states, we carried out a control experiment in which the STM tip was passivated with mercaptoethanol (MCE) to suppress thiol-gold bond formation. As shown in Fig. R11 c, both Peak II and Peak III vanished under these conditions. This result demonstrates that covalent coupling between surface-exposed cysteine residues and the gold tip is a prerequisite for observing large conductance values (>1 nS, corresponding to Peaks II and III), in agreement with our previous work (*Proc. Natl. Acad. Sci.* 116, 5886-5891 (2019)). A related question is whether different binding through Cys20 or Cys502, could generate resolvable conductance differences. Although these sites correspond to different charge-transport lengths, our earlier study (*ACS Nano* 16, 1671-1680 (2022) and *Nano Lett.* 19, 4017-4022 (2019)) showed that once a strong chemical contact is formed, the length dependence of protein conductance is weak ($\beta \approx 0.1$ nm^{-1}). Given the broad distribution of peaks in I-V histograms (Fig. R11), corresponding to the limited conductance resolution of the technique, such minor geometric differences are not expected to be resolvable.

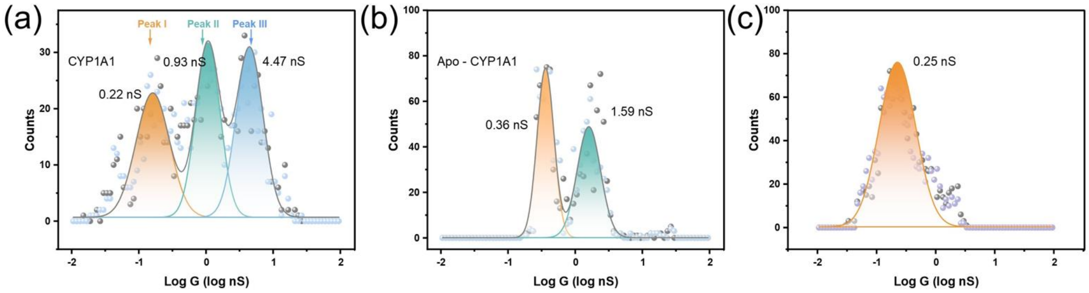

Figure R11 Histograms of conductance distribution for CYP1A1 under different conditions. (a-b) Conductance histograms of CYP1A1, apo-CYP1A1, (c) Protein conductance measured with MCE-passivated Au tips showed only Peak I (Peaks II/III absent), with triplicate values of 0.23, 0.25, and 0.26 nS.

The initial assignment of Peaks II and III was based on control experiments using the apo-enzyme, a strategy widely adopted in bioelectronic studies to differentiate transport pathways, for example, in investigations of azurin by David and coworkers (*Proc. Natl. Acad. Sci.* 121, e2405156121 (2024)). In our case, removal of the heme center led to the disappearance of Peak III while Peak II remained. This observation suggests that Peak II corresponds to a protein-matrix mediated pathway, whereas Peak III is associated with a metal-centered transport channel. We acknowledge, as the reviewer correctly noted, that removal of the heme inevitably perturbs the protein conformation (As evidenced in CD measurement Fig. S20), and thus this control is not entirely ideal. Nevertheless, the specific contribution of the redox-active center to charge transport may be independently supported by our QM/MM and DFT calculations, as discussed in detail in our response to Question 4.

Finally, we emphasize that conformational changes induced by cofactor and substrate binding systematically influence both Peak II and Peak III. This is demonstrated by our comprehensive CD spectroscopy measurements, which reveal a clear positive correlation between α-helical content and conductance (Fig. R9 and new Figs. S24-S25). Structurally, the active-site pocket of CYP1A1 is framed by the F and I helices (*J. Biol. Chem.* 282, 14348 (2007)), which contain dense networks of aromatic residues that facilitate electronic coupling. Binding of NADPH or BaP induces conformational

rearrangements in these helices, as detected by CD, thereby modulating the structural pathways for charge transport through both the protein matrix (Peak II) and the heme-mediated channel (Peak III). Superimposed on this structural effect, the redox state of the heme iron exerts a particularly strong influence on Peak III, as supported by our electrostatically embedded QM/MM and transport calculations. We therefore conclude that conformational modulation and redox tuning act cooperatively, and their individual contributions are intrinsically difficult to disentangle experimentally.

We thank the reviewer again for raising this insightful point. We have revised the manuscript to more clearly articulate this interpretation, and we hope that these clarifications and the unified presentation in the revised manuscript address the reviewer's concerns regarding internal consistency. (Page 8, lines 170-214; Page 11, lines 255-316; Page 14, lines 320-360).

3. The manuscript further assumes that NADPH binding does not induce conformational changes because the substrate is absent. This is not necessarily correct, as cofactor binding often affects the structure or dynamics of P450 systems. Since this assumption is used to attribute the conductance enhancement solely to the redox change, it should be justified or treated with more caution.

Response: We sincerely thank the reviewer for pointing out this mistake. The reviewer is entirely correct. As demonstrated by our supplementary CD measurements, the addition of the cofactor induces a pronounced change in the secondary structure of the enzyme. Specifically, the α-helical content increases by approximately 9% (Figs. R9a and R9b), which shows a strong correlation with the conductance according to the correlation analysis (Fig. R9c). We have therefore revised the manuscript to state that the conductance enhancement induced by NADPH arises from the synergistic effects of conformational rearrangement and redox-state tuning of the active center. We hope that this revision provides a more accurate interpretation and satisfactorily addresses the reviewer's concern.

"However, it should be noted that both coenzyme binding and substrate turnover

induce significant conformational changes that also modulate protein conductance (Fig. 2). Thus, isolating the individual contribution of the heme redox state from that of conformational effects remains challenging. These factors likely act synergistically to regulate electronic coupling and charge-transport efficiency." (Page 15, lines 355-360).

4. On the computational side, the DFT calculations are based on a highly truncated model (porphyrin + axial cysteine), which may not describe correctly the spin state or orbital distribution. The use of B3LYP for Fe(III)/Fe(II) redox systems is also questionable. Importantly, no transport-related quantities are computed, so the calculations do not directly support the interpretation of the conductance. At minimum, the limitations of this model should be explicitly addressed.

Response: We thank the reviewer for point out the limitations of the calculation methodology. As suggested, we have substantially refined our computational approach to address these concerns.

Firstly, to move beyond the highly simplified model and explicitly account for the influence of the protein environment, we adopted a hybrid quantum mechanics/molecular mechanics (QM/MM) framework with electrostatic embedding implemented in ORCA 6.0.1 (As detailed in Method). In this scheme, the QM region, comprising the heme iron, porphyrin, and axial cysteine ligand, is polarized by point charges representing the full protein matrix derived from the AMBER force field, and solvent effects are included through a conductor-like polarizable continuum model (CPCM). We further performed a systematic screening of all relevant spin multiplicities for both the Fe^{2+} and Fe^{3+} states (Table R1), selecting the quintet and sextet states, respectively, as they yielded the lowest energies and minimal spin contamination. The exchange-correlation functional was upgraded to ωB97M-V, which is more appropriate for transition-metal systems than B3LYP because it reduces self-interaction errors and incorporates nonlocal correlation effects that are important for accurately describing interactions within the heme pocket. These simulations show that reduction of the heme iron from Fe^{3+} to Fe^{2+} narrows the HOMO-LUMO gap and shifts the LUMO closer to

the Fermi level of the gold electrodes and the redox potential levels of nearby aromatic residues (Figs. R10a and new S22).

Table R1. Exploration of spin multiplicities for iron species (newly added in Supplementary table S2)

Species	spin multiplicity	<S**2>	Ideal value S*(S+1)	Energy (a.u.)
Fe(II)	1			-3744.23
	3	3.23	2	-3744.34
	5	6.22	6	-3744.35
	7	13.18	12	-3744.33
Fe(III)	2	2.92	0.75	-3743.85
	4	4.92	3.75	-3743.84
	6	8.99	8.75	-3743.85
	8	15.92	15.75	-3743.84

S denotes the spin quantum number. <S**2> represents the total spin operator squared, whose theoretical eigenvalue is given by S(S+1).

Moreover, as suggested by the reviewer, to establish a direct connection between the electronic structure and the measured conductance, we have carried out first-principles quantum transport calculations using the non-equilibrium Green's function formalism combined with DFT (NEGF-DFT). Due to the high computational cost, the transport calculations were carried out on the active-site cluster with the geometries and orientations extracted from fully optimized QM/MM structures. The calculated transmission coefficient near the Fermi energy is significantly higher for the Fe^{2+} state than for Fe^{3+} (Fig. R10b), providing direct theoretical support for the enhanced conductance observed experimentally in the reduced form.

We have revised the manuscript to incorporate these updated calculations (Page 14,

lines 320-360) and to expand the discussion of the remaining limitations of the model (Page 25, lines 608-611). We hope that these methodological improvements enhance the rigor of the electronic-structure and transport analysis and satisfactorily address the reviewer's concerns.

5. The kinetic analysis also requires clarification. The approximation that the change in conductance is proportional to the ES population is strong, especially in single-molecule experiments where conductance depends on contact geometry and molecular orientation. Moreover, the different Km values obtained (from conductance, GC-MS, and Lineweaver–Burk) differ by more than a factor of three. These discrepancies should be discussed more directly rather than simply stating that they are of the same order of magnitude.

Response: We sincerely appreciate the reviewer's insightful observation regarding the discrepancies among the reported K_m values. We believe that such differences may arise from both the methodology used to extract K_m and the altered enzymatic behavior in a heterogeneous, surface-immobilized system, as discussed below.

For the conductance-based analysis, we determined K_m using both nonlinear sigmoidal fitting and the Lineweaver-Burk plot, yielding apparent values of 43.2 μM and 24.2 μM, respectively. It is well recognized that double-reciprocal transformations amplify experimental uncertainty, particularly at low substrate concentrations, often leading to biased parameter estimates (*Fundamentals of Enzyme Kinetics* (ed Athel Cornish-Bowden) 200-211 (Butterworth-Heinemann, 1979) and *Biochem. J.* 137, 143-144 (1974)). Consequently, nonlinear regression is generally considered the more reliable approach in modern enzymology, and we therefore consider the value obtained from sigmoidal fitting to be the more reliable estimate.

When compared with literature values for CYP1A1 (K_m = 19.0-25.6 μM), the apparent K_m of 43.2 μM obtained from conductance measurements is noticeably larger. We attribute this difference to the heterogeneous nature of our system, in which the

enzyme is immobilized on a gold surface, and catalysis is partially influenced by mass-transport limitations. To quantify this effect, we estimated the Damköhler number (Da) and obtained $Da \approx 0.23$ (See Method), placing the system in a mixed kinetic–diffusion regime. Under these conditions, the apparent K_m is expected to exceed the intrinsic value by a factor of approximately $1+Da \approx 1.23$, which yields a corrected value (~34.9 μM) in close agreement with reported solution-phase measurements.

The remaining discrepancy between the K_m values obtained from GC-MS (90.6 μM) and conductance measurements (43.2 μM) likely reflects differences in the underlying kinetic observables and model assumptions. In the GC-MS assay, K_m is derived from the rate of BaP consumption, whereas in conductance measurements, it is inferred from changes in enzyme conductance that reflect the population of the enzyme-substrate complex. Although these quantities are related, they probe distinct aspects of the catalytic cycle and may therefore yield systematically different apparent parameters.

We thank the reviewer again for raising this important issue, which has helped improve the rigor and clarity of our kinetic analysis. We have expanded the discussion in the manuscript to clarify these points.

"For comparison, the classic Lineweaver-Burk plot was applied to the same data set, yielding a K_m^{app} of 24.2 μM (Fig. 4d). The discrepancy between the two calculation methods likely arises because the double-reciprocal transformation distorts the experimental error structure, particularly at low substrate concentrations, making it inherently less accurate than nonlinear regression[43]. " (Pages 18, lines 419-423).

"To further validate the model, gas chromatography-mass spectrometry (GC-MS) was used to independently determine the reaction rate under identical conditions, yielding a K_m of 90.6 μM (Fig. 4c, green line). This value is about twofold higher than that obtained from the conductance measurements, likely reflecting differences in the underlying observables and model assumptions. In the GC-MS assay, K_m is derived from the rate of BaP consumption, whereas in the conductance measurements, it is inferred from changes in enzyme conductance that reflect the population of the enzyme-substrate complex. Although these quantities are related, they probe distinct aspects of

the catalytic cycle and may therefore yield systematically different kinetic parameters." (Pages 18, lines 424-432).

"Compared with previous literature values reporting K_m for CYP1A1-catalyzed BaP metabolism (19.0 - 25.6 μM)[45,46]*, the 43.2 μM obtained from conductance measurents is significantly higher. This deviation likely reflects the heterogeneous nature of the conductance assay, in which the enzyme is immobilized on a gold surface. Under these conditions, the observed reaction rate is governed by both intrinsic catalytic kinetics and the mass transport of substrate to a spatially confined active site. To quantify the influence of diffusion,we calculated the Damköhler number (Da) and obtained a value of approximately 0.23 (Methods). This places the system in the mixed kinetic-diffusion regime (0.1 < Da < 10), rather than in the limits of purely kinetic-controlled (Da ≪ 0.1) or purely diffusion-controlled (Da ≫ 10) behavior. In this regime, the experimentally determined K_m^{app} is expected to be larger than the intrinsic kinetic constant by a factor of approximately (1 + Da) ≈ 1.23."* (Pages 18, lines 433-444)

6. Error bars must be included in all the data presented along the manuscript.

Response: We appreciate the reviewer's valuable suggestion. In response, we have added error bars to all figures in the main text and Supplementary Information. All data points are now reported as mean ± standard deviation (s.d.), where the s.d. reflects the variability from at least three independent measurements or, where applicable, the uncertainty associated with the fitting procedure.

7. Finally, several conclusions in the manuscript rely on attributing conductance changes to a single effect (redox state, conformational change, or contact geometry), but the experiments do not fully isolate these contributions. A more critical discussion of these limitations would improve the clarity and credibility of the work.

Response: We appreciate the reviewer's insightful suggestions and fully agree that the current study still has certain limitations. In response, as discussed in Q1 and Q2, we

have performed complementary experiments and analyses to provide additional information and strengthen the supporting evidence. Specifically, we conducted tip passivation experiments to elucidate the role of chemical coupling in protein conductance, carried out systematic CD spectroscopy to characterize enzyme conformational changes during catalysis, analyzed the correlation between enzyme conductance and conformational dynamics, and refined the theoretical calculation methodology to more accurately represent the model and better elucidate the underlying mechanism.

Nevertheless, as noted by the reviewer, some limitations remain and warrant further clarification. In particular, the effects of redox state and conformational changes on enzyme conductance are intrinsically coupled, making it challenging to isolate the contribution of a single factor while holding others constant. In addition, although the theoretical model has been improved by incorporating protein environmental effects and solvent contributions, it remains a simplified representation of the system.

In response to the reviewer's suggestion, we have updated the supplementary results and adopted more precise language in the assignment of the conductance peaks. In addition, we have expanded the discussion on the remaining limitations of this study in the Conclusion Section. We hope that these revisions adequately address the reviewer's concerns. It reads as follows:

"*Despite these advancements, several limitations of the present study should be acknowledged. The contributions of cofactor redox state, protein conformational changes, and electrode-protein contact geometry to the measured conductance are intrinsically coupled, complicating the isolation of individual factors under experimental conditions. Moreover, although theoretical calculations were extended to account for protein environmental effects and solvent contributions, they remain necessarily simplified owing to the computational complexity of electronic structure calculations for large biomolecular systems. Nonetheless, this study establishes a successful proof of concept for protein-based bioelectronic platforms capable of resolving transient enzymatic kinetics with high temporal resolution, offering a*

promising approach for future investigations in mechanistic enzymology, bioelectronics, and drug discovery." (Page 25, lines 604-615)

8. In summary, I recommend publication after major revisions that strengthen the mechanistic interpretation, clarify the logic used to assign conductance states, and discuss more explicitly the limitations of the experimental and computational analyses.

Response: We sincerely thank the reviewer for the constructive and helpful comments. As suggested, we have carried out additional experiments to better support our conclusions, adopted more accurate and logically consistent interpretations of the data, and expanded the discussion of the limitations of this study. We hope that these comprehensive revisions adequately address the reviewer's concerns.

Once again, we wish to express our sincere appreciation for the reviewer's insightful comments, which have significantly contributed to improving the scientific rigor and overall quality of this work.

Reviewer #4 (Remarks to the Author):

General Comments: I co-reviewed this manuscript with one of the reviewers who provided the listed reports. This is part of the Nature Communications initiative to facilitate training in peer review and to provide appropriate recognition for Early Career Researchers who co-review manuscripts.

Response: Thank you for your time and for the valuable comments on our manuscript. We have carefully considered each suggestion and made every effort to address all of the reviewers' questions and concerns. We hope that the revised manuscript adequately addresses these points.

Point-to-point responses to the reviewers' comments

We sincerely thank all the reviewers for the time and effort they dedicated to evaluating our work. We are grateful to Reviewers 1, 3, and 4 for their positive feedback and to Reviewer 2 for the critical comments. We have carefully considered each point and revised the manuscript accordingly. Our point-by-point responses are provided below. For clarity, reviewer comments are presented in plain text, followed by our responses in blue, and the revised text in the manuscript is shown in *italics*.

Reviewer #1 (Remarks to the Author):

General Comments: The reviewer is happy with the changes.

Response: We sincerely appreciate the reviewer's positive evaluation and kind comments, as well as the time and effort invested in reviewing our work.

Reviewer #2 (Remarks to the Author):

General Comments: This manuscript presents a conceptually appealing and innovative idea—"Electrical Monitoring of Enzymatic Catalytic Dynamics at the Single-Molecule Level." However, despite the novelty of the concept, I have substantial concerns regarding the validity of the experimental observations and the robustness of the data analysis. A critical issue arises from the authors' own acknowledgment that "Given the limited conductance resolution of the I-V histogram, the conductance features of the enzyme-NADPH complex and the ES complex are unlikely to be resolved as separate, well-defined peaks." This admission fundamentally undermines the central claim of the study. If the conductance technique lacks the resolution to distinguish between key catalytic states, it raises serious doubts as to whether the observed signals genuinely reflect dynamic enzymatic catalytic processes or merely arise from unresolved, averaged events. Furthermore, the data processing methodology exacerbates these concerns, suggesting that the observed "states" may represent

ensemble averages rather than discrete, functionally relevant intermediates. One of the major scientific issues is that the authors mistake the duration distribution and dwell time/lifetime distribution. Based on this misunderstanding, their duration distribution follows the exponential distribution rather than bell-shape, increasing the unreliability of their work. Given these fundamental limitations, it remains unclear whether the electrical signals reported indeed capture genuine catalytic dynamics at the single-molecule level.

Response: We thank the reviewer for highlighting the conceptual innovation of our work. However, we respectfully disagree with the reviewer regarding the validity of the experimental observations and the robustness of the data analysis, as we believe that these concerns arise from a fundamental misunderstanding of the measurement techniques and data processing methods.

(1) Regarding the concern about the limited conductance resolution of the I-V histogram for dynamic catalytic monitoring.

We must respectfully clarify that this concern stems from a fundamental technical misunderstanding of two distinct protein conductance measurement techniques. Specifically, the reviewer incorrectly conflated the (i) population-based I-V characterization, used for elucidating electron transport mechanisms, with the (ii) single-molecule, high-temporal-resolution I-t measurements, used for real-time monitoring of enzymatic dynamics. These two methods are intrinsically different in methodology, data processing, biophysical interpretation, and intended purposes within this study. Importantly, we used the I-t technique for the dynamic catalytic process study, rather than the I-V histogram. Expecting I-V histograms to resolve dynamic states is scientifically impractical and also outside the intended scope of the technique.

We understand the reviewer's concern that, due to the limited conductance resolution, the I-V histogram comprises signal contributions from both enzyme-NADPH and ES complexes; **however, the I-t measurement fundamentally rules out this possibility**. This is because we only analyze the I-t traces showing TN features, which are a definitive signature of active enzymatic processes. Our control experiments (Fig. S28)

demonstrate that TN features are absent under non-catalytic conditions, confirming that the analyzed I-t signals originate exclusively from catalytically active enzymes. Furthermore, as noted in the manuscript (Page 19, Line 463), even under full enzymatic conditions, we found that only ~40% of the recorded I-t traces exhibit TN signals, while the remaining ~60% (lacking TN features) are rejected from analysis. These discarded traces correspond to inactive or non-catalytic states, which may align with what the reviewer refers to as "substates". Therefore, due to the data filtering process and the inherent single-molecule nature of the I-t technique, the analyzed trajectories do not include contributions from inactive enzymatic events, ensuring that the extracted signals from I-t measurements genuinely reflect dynamic enzymatic catalytic processes.

To facilitate a clearer comparison between I-V and I-t measurements, we summarize the detection principles and key characteristics of these two techniques in Figure R1 and Table R1. We apologize for the need to reiterate the technical details at some length. It appears that certain misunderstandings of the methodology have led to serious misinterpretations of our experimental results and conclusions.

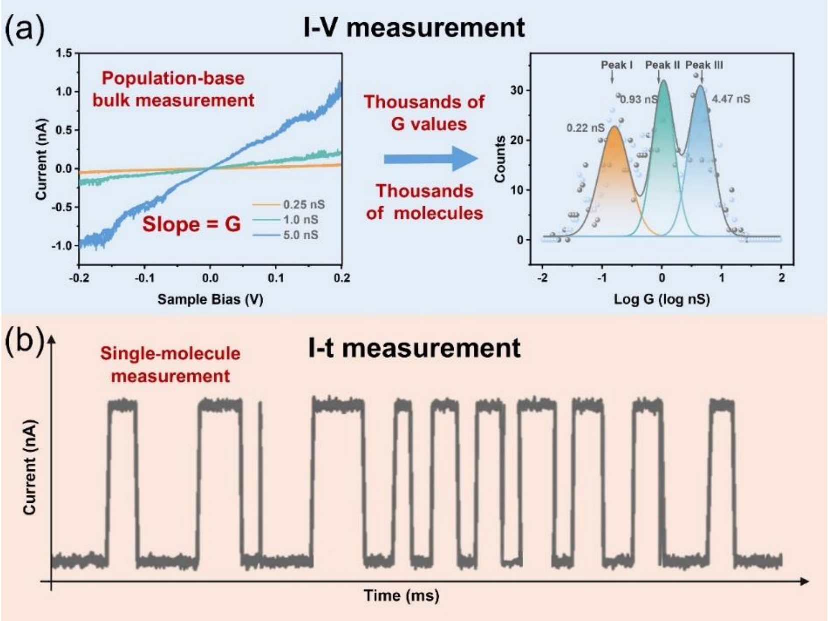

Figure R1. Schematic illustration of the differences between I-V and I-t measurement techniques. I-V based histograms are statistical, population-based constructs derived from the slopes of thousands of individual I-V curves, corresponding to thousands of enzyme molecules. In contrast, the I-t measurement is a true single-molecule technique,

which monitors real-time current fluctuations from a single junction, with telegraph noise (TN) signals directly reporting on the catalytic dynamics of an individual enzyme.

Table R1. Comparison between population-based I-V and single-molecule I-t measurement

Feature	I-V Characterization (Population-based)	I-t Measurement (Single-molecule)
**Methodology**	Statistical construction from thousands of individual I-V curves.	Real-time monitoring of current fluctuations in a single junction.
**System State**	**Ensemble-averaged** (analogous to bulk UV-Vis or fluorescence).	**Discrete single-molecule** trajectories (analogous to nanopore/AFM).
**Temporal Resolution**	Static; represents a steady-state conductance snapshot.	High (20 μs at 50 kHz); captures transient intermediates.
**Biophysical Meaning**	Most probable electron transport pathways through the protein.	Stochastic catalytic transitions and turnover events.
**Data Interpretation**	Broadening reflects molecular heterogeneity and junction variability.	Telegraph noise (TN) reports on conformational and chemical dynamics.
**Intended Purpose**	To elucidate fundamental electron transport mechanisms.	To monitor real-time enzymatic catalytic dynamics and kinetics.
**Selectivity**	Includes all junctions (**active, inactive, or "dead" enzymes**).	Selectively filters for **active enzymes** displaying TN fluctuations.

(a) <u>Differences in methodology</u>: population-based (I-V) measurement *vs.* single-molecule (I-t) measurement.

The difference can be simply understood by considering I-V measurements as analogous to bulk techniques such as UV-vis or fluorescence spectroscopy. As shown in Figure R1a, constructing an I-V histogram requires the collection of thousands of I-V curves, each corresponding to a different protein molecule. From each curve, only the slope (i.e., conductance, G) is extracted to build the histogram. Consequently, the I-

V histogram is inherently a statistical, population-based measurement lacking temporal resolution, analogous to ensemble spectroscopic methods. In contrast, I-t measurements are true single-molecule observations, capturing the current fluctuations from an individual trapped enzyme at a high sampling rate of 50 kHz (20 μs resolution). This high-bandwidth temporal domain enables us to track transient state changes during the catalytic cycle in real time (Figure R1b). Unlike I-V histograms, I-t trajectories are not subject to ensemble averaging across different molecules, allowing for the unambiguous identification of catalytic events over extended observation times. Consequently, the static, ensemble-averaged I-V histogram is inherently unsuitable for resolving dynamic catalytic states at the single-enzyme level.

(b) Differences in biophysical interpretation: electron transport pathways *vs.* dynamic catalytic events.

The peaks in I-V histograms represent the most probable electron transport pathways across a large population of enzyme molecules, while the broadness of the peaks reflects the molecular heterogeneity and junction variability. This is analogous to the broad emission features commonly observed in fluorescence spectra, which arise from distributions of vibrational and rotational energy states. In I-V measurements, statistical averaging mitigates such heterogeneity, enabling more reliable interpretation of protein conductance mechanisms. This approach is well established in STM break-junction and nanopore studies (e.g., *Science* 301,1221-1223(2003); *Nat. Catal.* 6, 266-275 (2023); *Chem. Soc. Rev.*, 38, 2360-2384 (2009)). In contrast, I-t measurements provide the temporal resolution required to monitor transient and dynamic catalytic events at the single-molecule level. The TN fluctuations observed in I-t traces are characteristic signatures of active enzymes under catalytic conditions, as confirmed by our control experiments (Fig. S28) and consistent with our previous report (*ACS Nano* 14, 1360-1368 (2020)). Crucially, analysis of TN signals inherently excludes contributions from inactive or nonfunctional enzymes (e.g., CYP1A1-NADPH in the absence of BaP), as I-t traces without TN features are filtered out during data processing.

(c) Differences in intended purposes: mechanism study *vs.* dynamic study.

The intended purposes of these two techniques in our study are fundamentally distinct. We utilized I-V characterization to elucidate protein conductance mechanisms, thus providing a theoretical framework for interpreting conductance-based catalytic monitoring. In contrast, I-t measurements were specifically used to monitor catalytic dynamics at the single-molecule level, leveraging their high temporal resolution to resolve enzymatic processes in real time. Because of these fundamental differences in technical features and biophysical meanings, the results from these two techniques should not be conflated. For example, I-V measurements enabled us to distinguish conductance features between wild-type CYP1A1 and Apo-CYP1A1, revealing distinct electron transport pathways (metal-mediated *vs.* protein-matrix pathways), and to evaluate the influence of heme redox states via coenzyme addition (NADPH/NADH). Conversely, I-t measurements were uniquely suited for resolving catalytic dynamics. The distinct purposes of these two measurements arise from their fundamentally different technical characteristics. We do not expect static, ensemble-averaged I-V histograms to resolve the dynamic catalytic states of a single enzyme molecule. By analogy, fluorescence spectroscopy is not employed for such purposes, as it yields only ensemble-averaged signals from a large population of enzymes.

(d) The resolution of the I-V histogram does not affect the validation of the catalytic dynamic study by I-t detection.

The I-V histogram does not affect the validation of the I-t results, because these two techniques function under different detection principles. Although the I-V histograms under full enzymatic conditions may comprise both information from the CYP1A1-NADPH and ES complex, the I-t measurement technically and fundamentally excludes the possibility of "superposition of multiple substates", such as contributions from enzyme-NADPH or inactive enzymes. That is because we only analyzed I-t traces with TN signals (~ 40% of the total I-t traces), which are a definitive signature of active enzymatic processes, as evidenced by our control experiments, in which I-t traces show no TN features under non-catalytic conditions (Fig. S28). This observation is also

consistent with our previous study, in which the enzymatic activity of DNA polymerase was monitored via protein conductance using a similar I-t technique (*ACS Nano* 14, 1360-1368 (2020)). During the I-t measurement, it is indeed possible that the probe may pick up inactive enzymes (e.g., enzyme-NADPH or dead enzyme), as mentioned in the main text (Page 19, line 463), with approximately 60% of traces under 10 μM BaP showing no TN signals. However, this kind of TN-featureless traces is considered to represent inactive enzyme molecules and is therefore discarded. This operation ensures that all TN signals collected from I-t measurements reflect catalytic events of active enzymatic catalysis.

(2) Regarding the data processing methodology

It appears that the reviewer may have inadvertently misinterpreted the histograms shown in the right panels of Figs. 5a-c (Fig.5 in the revised manuscript) as duration distributions (as suggested in Comments 9 and 10 by the reference to a "bell-shaped distribution"). If this is the case, we respectfully clarify that these histograms represent the stacking of current amplitudes (or conductance amplitudes) along the Y axis, rather than the temporal distribution. As the reviewer can see, these histograms are plotted in units of nA (current) or nS (conductance), and are used to evaluate the amplitude distribution of distinct conductance states. From these histograms, the TN amplitudes (I_F) were extracted and subsequently analyzed in Fig. 5d and Fig. 5g. In contrast, dwell-time distributions were derived from HMM/QUB analysis of I-t trajectories (as exemplified in Fig. 5e), followed by monoexponential fitting to obtain the corresponding lifetimes (Fig. 5f).

Additionally, we emphasize that the data-processing methodology for the I-t measurements in this study follows mathematically rigorous standards in the field of single-molecule biophysics (e.g., *Nat. Nanotechnol.* 16, 1214-1223 (2021); *Sci. Adv.* 8, eabm8149 (2022)). We respectfully disagree that we have mistaken duration distributions for dwell-time distributions. We also respectfully encourage the reviewer to read these papers for clarification regarding the definition and analysis of dwell

time/lifetime in single-molecule bioelectronics (https://doi.org/10.1038/s41565-021-00959-4; https://doi.org/10.1126/sciadv.abm8149). Generally, the dwell time of TN signals is defined as the stochastic duration (τ values in Fig. 5e) during which an individual enzyme molecule resides in a discrete functional conductance state (S1-S3) before transitioning to a subsequent state, which are exclusively obtained from high-temporal-resolution I-t measurement. Each conductance state corresponds to a distinct substrate-binding or intermediate state, as validated by our control experiments using metabolic intermediates (S1: 7-OH-BaP; S2: 7,8-D-BaP; S3: 7,8-E-BaP). The hallmark feature of dwell time is its direct reporting on the underlying kinetic rate constants (k) of the biological process. Statistically, for a memoryless single-molecule Markov process, the dwell-time distribution fundamentally follows a single-exponential decay (*Nat. Commun.* 16, 9858 (2025)), i.e., $P(t) = k \cdot \exp(-kt)$, which is consistent with our results (Fig. 5f).

We hope that the above clarification adequately addresses the reviewer's concern.

1. Regarding Fig. R3c (Fig 2c), the R^2 fit based on only three data points lacks sufficient statistical significance and does not support the conclusion of a strong correlation. Furthermore, I have substantial concerns about Fig. R3def (Fig 2def). First, α-helical structures in enzymes undergo continuous bending, unfolding, and refolding during catalysis, processes closely tied to catalytic functon (Biochemistry 2025, 64, 4281−4295). This implies that the proportion of α-helical content in an enzyme fluctuates dynamically throughout the catalytic cycle. Given that a single circular dichroism (CD) measurement takes approximately 5–10 minutes, and that both the reaction rate and total reaction time vary with BaP concentration, a critical question arises: do the α-helical percentages obtained from CD measurements at six different BaP concentrations represent the state before, during, or after the reaction? Can these values be considered comparable across different states? The CD experiments merely indicate that the α-helical content changes during the reaction; they do not substantiate the claimed correlation.

Response: We thank the reviewer for the opportunity to clarify the statistical and physical basis of our CD data analysis. In response to concerns about statistical significance, we have expanded our dataset to ensure robustness beyond the initial observations. The correlation between protein conductance and α-helical content is now supported by four independent conditions, including CYP1A1, CYP1A1-NADH, CYP1A1-NADPH, and CYP1A1-NADPH-BaP, as presented in the updated Figure R2 (also Fig. 2c in the revised manuscript). More importantly, this relationship is independently validated by our concentration-dependent experiments (Fig. R3, Figs. 2d-f), comprising 6 data points and yielding good linear fits (Figs S24-25). Additionally, we want to emphasize that the purpose of CD measurements is to quantify the conformational changes of the enzyme under different conditions.

We agree with the reviewer that, at the level of an individual enzyme molecule, structural fluctuations occur during the catalytic cycle. However, it is important to note that CD spectroscopy is inherently an ensemble-averaged (bulk) technique rather than a single-molecule probe, and it measures the weighted average over a large population of molecules. At a given BaP concentration, the population of the enzyme in different conformational states (e.g., the substrate-binding state) reaches a dynamic equilibrium that depends on BaP concentration. In our experiments, measurements at six concentrations were conducted independently rather than via continuous titration. The CD spectrum acquisition was initiated immediately after BaP addition, and each measurement was completed within 2 min. Under these conditions, considering the relatively high BaP concentration (μM range) and the enzyme turnover number (≈ 2.0 s^{-1}), substrate consumption during the measurement is negligible, and the reaction rate can be approximated as the initial rate at a given BaP concentration.

Regarding the temporal nature of CD measurements, we further emphasize that this bulk technique does not resolve distinct catalytic states, analogous to our population-based I-V histograms. Instead, CD signals reflect the time-averaged distribution of conformational states under dynamic equilibrium during catalysis. This population-weighted average is directly comparable to the ensemble-averaged conductance

obtained from thousands of I-V measurements. As the substrate concentration increases, the equilibrium shifts toward substrate-bound states (ES complex), and this shift is consistently captured by both techniques. Therefore, the two measurements are fundamentally comparable and together establish a statistically sound relationship between population-level structural changes and electronic properties. This approach is standard and widely accepted in enzymology for linking structure to function (e.g., *Materials* 12, 1022 (2019); *Nucleic Acids Res.* 41(7), e7 (2013)).

We hope that the above explanation adequately addresses the reviewer's concern. The revised figures have been updated accordingly in the manuscript. Also, we have incorporated Figure R3 into the supplementary information to better support the BaP-dependent linear relationship between protein conductance and α-helical content of the enzyme (Figs. S24-25).

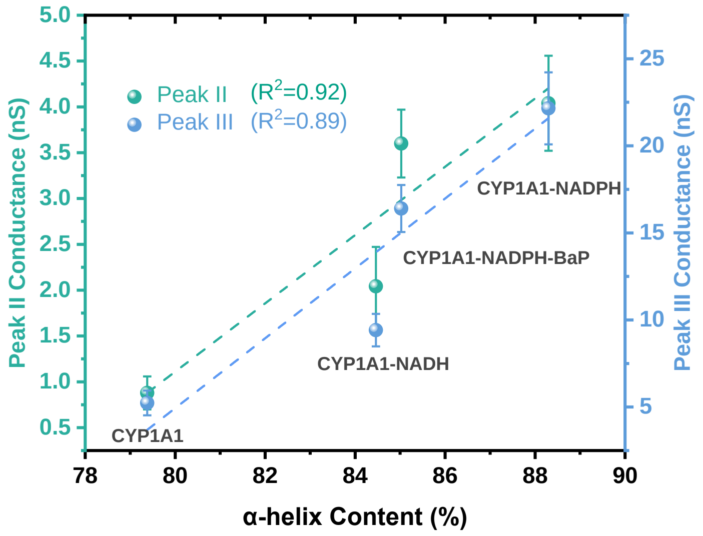

Figure R2. Relationship between α-helical content and conductance of the enzyme under different conditions.

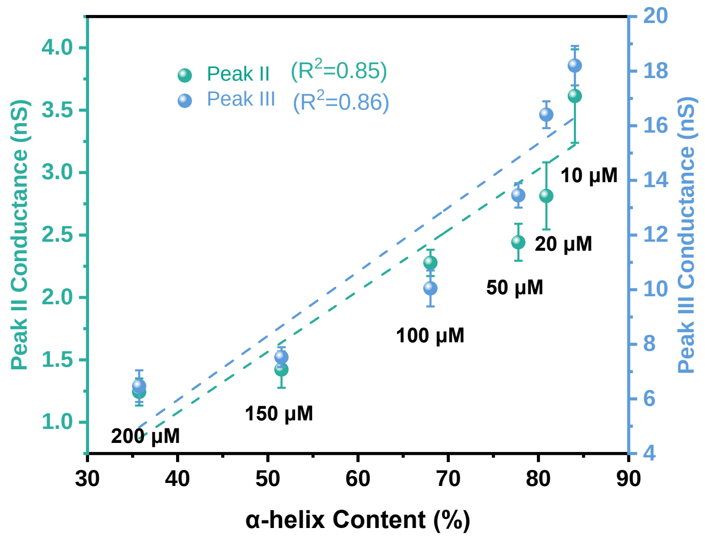

Figure R3. In the CYP1A1-NADPH system with the addition of 10-200 μM BaP, the α-helix content is positively correlated with protein conductance (Peaks II and III).

2. Although your explanation—"Given the limited conductance resolution of the I-V histogram, the conductance features of the enzyme-NADPH complex and the ES complex are unlikely to be resolved as separate, well-defined peaks"—may be an honest acknowledgment, it raises a concerning issue. The limited resolution may cause multiple states to merge into a single peak, obscuring the true underlying scientific reality. This could even lead to erroneous conclusions when such data are used as the basis for analysis (see comment 3). A similar concern arises: could this averaging effect, which masks rare events due to insufficient resolution, also be present in other experimental measurements?

Response: We thank the reviewer for this comment, but we must respectfully clarify that the concern about "limited resolution" arises from a categorical misunderstanding of the complementary roles of I-V and I-t measurements, as detailed in our response to the **General Comments**. The "averaging effect" the reviewer describes is not a flaw in our experimental approach; rather, it is the inherent and intended characteristic of any ensemble-based characterization, such as I-V histograms, UV-Vis, or fluorescence spectroscopy, and is also widely documented in the single-molecule bioelectronics field (e.g., *Science* 301,1221-1223(2003); *Nat Catal* 6, 266-275 (2023); *Chem. Soc. Rev.*, 38,

2360-2384 (2009)). We exclusively use the population-based I-V technique to establish the mechanistic foundation of protein conductance, such as identifying metal-mediated transport pathways or assessing the influence of redox states, rather than to resolve transient catalytic intermediates. The inability of a static I-V histogram to separate proximal conductance states is precisely why this technique is not used to derive our core conclusions regarding enzymatic dynamics.

The true "scientific reality" of discrete catalytic transitions is captured by our high-bandwidth I-t measurements, which operate at a 50 kHz sampling rate to circumvent the averaging inherent in ensemble methods. While the I-V histogram provides a statistical baseline of the most probable conductance pathways, the I-t trajectories resolve the stochastic, single-molecule catalytic events that define the catalytic cycle. Our control experiments (Fig. S28) demonstrate that these dynamic fluctuations (TN signals) are entirely absent under non-catalytic conditions, confirming that our I-t signals are genuine reports of enzymatic activity rather than artifacts of unresolved ensemble states. Importantly, the I-t measurements fundamentally ruled out the possibility of "merge of multiple states", as we analyzed only I-t traces with TN features. We rejected approximately 60% of TN-featureless I-t traces during data processing, corresponding to inactive enzymatic states (such as the enzyme-NADPH complex). Therefore, the lack of peak separation in a static histogram in no way undermines the validity of the time-resolved single-molecule data, as these two techniques are fundamentally different in their temporal resolution and intended scientific purpose. We hope that this clarification adequately addresses the reviewer's concern.

3. The amplitude-based analysis is concerning. In response to Point 6, the authors concede that the I-V method suffers from insufficient precision, such that conductance signals from distinct real-world events are averaged into peak shifts. Consequently, states exhibiting high-amplitude electronic fingerprints in I-t mode are more likely to represent a superposition of multiple substates—potentially corresponding to either the enzyme-NADPH complex or the ES complex. As long as conductance values cannot

be decomposed into individual single-molecule events, subsequent analyses and interpretations, particularly the identification of S3, remain problematic.

Response: We respectfully clarify that the reviewer's concern regarding the "superposition of multiple substates in I-t measurement" stems from a continued conflation of ensemble-averaged I-V histograms with single-molecule I-t trajectories. As explained above, these two techniques are fundamentally distinct in their methodologies, biophysical meanings, and intended roles in this study. The I-V is primarily used to elucidate conductance mechanisms, as it is a population-based (bulk) measurement lacking both single-molecule specificity and temporal resolution. In contrast, the I-t technique is a true single-molecule detection platform featuring high temporal resolution and is specifically employed to monitor the stochastic current fluctuations of an individual enzyme molecule during catalysis.

It is very important to note that the I-t detection fundamentally and effectively ruled out the possibility of "superposition of multiple substates", such as contributions from enzyme-NADPH or inactive enzymes. That is because we only analyzed the TN signals, which are a definitive signature of active enzymatic processes, as evidenced by our control experiments, in which I-t traces show no TN features under non-catalytic conditions (Fig. S28). This observation is also consistent with our previous study, in which the enzymatic activity of DNA polymerase was monitored via protein conductance using a similar I-t technique (*ACS Nano* 14, 1360-1368 (2020)).

The identification of states S0-S3 is based on relative current amplitudes (Fig. 5d) extracted directly from these high-bandwidth single-molecule I-t trajectories and is rigorously validated by control experiments using specific metabolic intermediates, such as 7-OH-BaP and 7,8-D-BaP (Figs. 5b-c and S30). The assignment of S3 is supported by its unique conductance signature (approximately 76% of the baseline, Fig. 5d), its characteristic lifetime (Fig. 5f), and its logical position within the established BaP metabolic pathway (Fig. 5h). Collectively, these results demonstrate that our analysis is both internally consistent and experimentally validated, and is fundamentally distinct from ensemble-averaged interpretations derived from static

histograms.

We hope that the above clarification adequately addresses the reviewer's concern.

4. The authors acknowledge that due to limitations in data analysis techniques, the observed peak shift represents an average of two types of events. However, the nature of this averaging remains ambiguous: is it a linear average of the two states or a nonlinear one? Given that both the E and ES complexes exhibit two peaks each, even a linear average would require appropriate weighting. In any case, ensemble averaging compromises the credibility of the analysis presented in this section.

Response: We thank the reviewer for the opportunity to clarify the statistical nature of the observed peak shifts. The concern regarding the "averaging" of states in the I-V histograms can be more easily understood by recognizing that this measurement is a population-based sampling technique, fundamentally analogous to bulk fluorescence or UV-Vis spectroscopy. The shift in peak position does not represent an ambiguous mathematical compromise, but rather a population-weighted average governed by the laws of mass action and chemical equilibrium. As the concentration of BaP increases, the probability of capturing an ES complex within the junction increases proportionally. The resulting conductance histogram reflects the distribution of the ensemble, with the peak position shifting as the relative occupancy of the active site changes.

This population weighting is quantitatively described by a standard binding isotherm, as evidenced by the fact that the conductance shift (ΔG) follows a hyperbolic saturation curve (Fig. 4b). By applying this model, we extract an apparent Michaelis constant (K_m^{app}) between 24.2 and 43.2 μM, which is highly consistent with established literature values for free CYP1A1. This approach is a mathematically valid and scientifically sound method for extracting thermodynamic parameters from ensemble data, effectively treating the conductance shift as a proxy for fractional occupancy, a standard practice in biophysical chemistry. Furthermore, this ensemble-level analysis is independently validated by the strong correlation with our circular dichroism data. Most importantly, this population-weighted averaging is confined to the I-V characterization

used for kinetic modeling and does not affect the high-resolution I-t measurements, which bypass ensemble limitations entirely by isolating individual molecules in real time. We hope that the above explanation adequately addresses the reviewer's concern.

5. Moreover, given the admission that the conductance value reflects an average of multiple events, certain statements in this section warrant reconsideration. For example: "This substrate-dependent conductance change is consistent with the variation in α-helical content extracted from CD measurements (Fig. 2f), yielding linear correlations for both Peaks II and III (Fig. S24-S25)." Similar concerns apply to the section title "Substrate concentration dependence of the CYP1A1 conductance" and the definition of ΔG.

Response: We thank the reviewer for the opportunity to clarify the biophysical interpretation of I-V histograms under varying BaP concentrations. As explained above, it is important to recognize that the I-V technique is fundamentally a population-based, ensemble measurement, conceptually analogous to bulk spectroscopic methods such as UV-vis, fluorescence, or CD spectroscopy. The conductance peak values derived from these histograms represent the statistical distribution of thousands of independent enzyme molecules sampled during the measurement. In our dynamic catalytic system, the conductance peak shift (ΔG) relative to the control reflects the fraction of enzyme molecules in the ES state, thereby reporting on the fractional occupancy of active sites. This approximation is scientifically well grounded and is also supported by Reviewer 3, who noted in the first round of revision that "*the approximation that the change in conductance is proportional to the ES population is strong, especially in single-molecule experiments…*" (Reviewer 3, Comment 5). Technically, this approach is directly analogous to conventional spectroscopic assays, in which peak shifts or intensity changes are used to quantify reaction kinetics, often through their proportionality to ES concentration. Since both I-V-derived conductance and α-helical content from CD measurements are ensemble-averaged observables, correlating them under identical equilibrium conditions represents a robust and widely accepted strategy

in biophysical chemistry (*Materials* 12, 1022 (2019)). This correlation provides key evidence linking population-averaged structural conformations to ensemble-averaged electronic properties.

The definition of ΔG as the shift in the ensemble-averaged conductance peak is thus physically meaningful, as it directly tracks the fractional occupancy of the ES complex across a large population of sampled molecules. Analogous to peak-shift analyses in traditional bulk kinetic assays, ΔG serves as a robust metric for quantifying concentration-dependent behavior at the ensemble level. This approach is further supported by its strong linear correlation with structural data (Figs. S24-25) and is consistent with established methodologies in single-molecule bioelectronics (*Materials* 12, 1022 (2019)).

Regarding the subtitle, we have revised it to "Substrate concentration dependence of conductance change in CYP1A1" to improve accuracy and clarity. We hope this clarification adequately addresses the reviewer's concern.

6. Closely related to the previous point, without resolving the sub-states within each peak, it is difficult to determine whether the observed effects stem from data analysis limitations or from diffusion-related processes.

Response: We thank the reviewer for the comment. We respectfully clarify that the determination of the Michaelis constant (K_m) in our study is derived from substrate concentration-dependent conductance shifts obtained from population-averaged I-V measurement. As explained in our responses to Comments 4 and 5, ΔG serves as a reliable indicator of the ES population at a given BaP concentration, thereby providing the basis for K_m calculation. This approach is scientifically rigorous and essentially consistent with established ensemble techniques, such as CD and fluorescence spectroscopy, in which kinetic parameters are extracted from macroscopic, concentration-dependent signal changes. In this specific analytical framework, resolving individual sub-states within a single peak is neither experimentally feasible nor theoretically necessary for determining K_m.

To address the concerns regarding diffusion-related effects, we have rigorously quantified the competition between mass transport and intrinsic catalytic kinetics by calculating the Damköhler number (Da). The obtained value (Da ≈ 0.23) indicates that the system operates in a mixed kinetic-diffusion regime. This analysis enables us to account for the role of diffusion explicitly, as the apparent Michaelis constant (K_m^{app}) is expected to exceed the intrinsic kinetic constant by a factor of approximately (1+Da) ≈ 1.23.

Furthermore, our approach to extracting Michaelis constant from single-molecule electronic measurements is consistent with established precedents in single-molecule enzymology (*Nat. Chem. Biol.* 2, 87-94 (2006); *Phys. Chem. B* 109, 19068-19081 (2005)). In this context, the peak position serves as the primary observable for kinetic analysis, and its systematic shift provides a mathematically sound and physically meaningful basis for our conclusions.

7. Although the authors reanalyzed the data using QUB and HMM, they did not update the dwell time statistics accordingly. Modifying the initial model and parameters in QUB inevitably alters the idealized trace, and thus the dwell time distributions must also change.

Response: We thank the reviewer for pointing out this mistake. We sincerely apologize for inadvertently overlooking the update Fig. 5f in the previous version of the manuscript after reanalyzing the data using HMM/QUB. The corrected figure has now been incorporated into Fig. 5 in the revised manuscript and is also shown as Figure R4 below. Compared with the previous fitting results, the extracted dwell-time distributions (τ_1 = 1.83 ± 0.33 ms, τ_2 = 1.52 ± 0.09 ms, τ_3 = 5.48 ± 0.54 ms) change slightly, but the overall kinetic trends and the central conclusions remain unchanged. We thank the reviewer again for this careful observation.

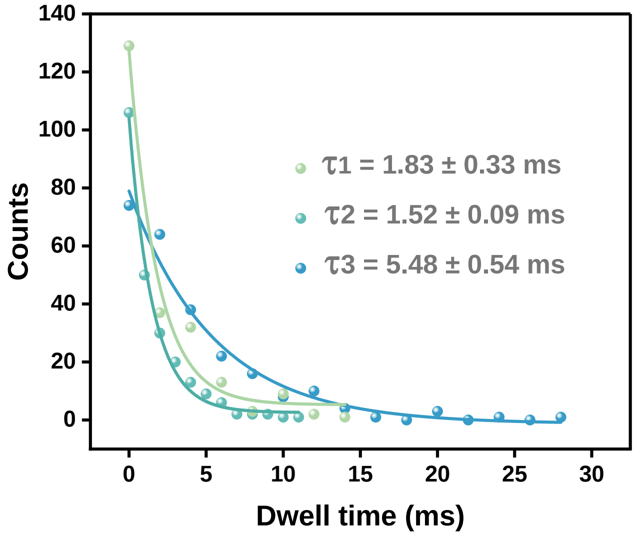

Figure R4. Dwell time distributions for conductance states S1, S2, and S3 obtained from QUB fits. Lifetimes were extracted via monoexponential fitting.

"As shown in Fig. 5f, the dwell time distributions for states S1, S2, and S3 follow monoexponential decay, yielding average lifetimes of $\tau_1 = 1.83 \pm 0.33$ ms (S1), $\tau_2 = 1.52 \pm 0.09$ ms (S2), and $\tau_3 = 5.48 \pm 0.54$ ms (S3), respectively." (Page 23, lines 558-560)

8. Given the unreliability of amplitude-based analysis, states exhibiting high-amplitude electronic fingerprints likely consist of multiple sub-states. Therefore, the reported lifetimes correspond to the aggregate of multiple events, rendering the statistical analysis of S3 lifetimes unreliable.

Response: We thank the reviewer for this comment. However, we respectfully disagree with the reviewer that amplitude-based analysis is unreliable due to the superposition of "multiple substates". As explained in our response to Comment 3, the I-t measurements fundamentally ruled out the possibility of signal contributions from inactive enzymes, such as the enzyme-NADPH or dead enzymes. This is because our analysis is restricted to I-t traces that exhibit TN features, which serve as a definitive signature of active enzymatic processes. Control experiments (Fig. S28) demonstrate that TN features are absent under non-catalytic conditions, confirming that the analyzed

signals originate exclusively from catalytically active enzymes. This observation is also consistent with our previous work, in which the enzymatic activity of DNA polymerase was monitored via protein conductance using a similar I-t approach (*ACS Nano* 14, 1360-1368 (2020)). Furthermore, as noted in the manuscript (**Page 19, Line 463**), approximately 40% of the recorded I-t traces exhibit TN signals, while the remaining ~60% (lacking TN features) are rejected from analysis. These discarded traces correspond to inactive or non-catalytic states, which may align with what the reviewer refers to as "substates". Therefore, due to the data filtration processing and the inherent single-molecule nature of the I-t technique, the analyzed trajectories do not include contributions from inactive enzymatic events, ensuring that the extracted signals are both specific and reliable.

The identification of states S0-S3 is based on relative current amplitudes (Fig. 5d) extracted from high-temporal-resolution, single-molecule I-t traces, and is rigorously validated through control experiments using defined metabolic intermediates (e.g., 7-OH-BaP and 7,8-D-BaP; Figs. 5b-c and S30). In particular, the assignment of S3 is supported by its distinct conductance signature (approximately 76% of the baseline; Fig. 5d), its characteristic lifetime (Fig. 5f), and its consistency with the established BaP metabolic pathway (Fig. 5h). In addition, the data processing methodology for I-t measurements in this study follows well-established and mathematically rigorous standards in single-molecule biophysics (e.g., *Nat. Nanotechnol.* 16, 1214-1223 (2021); *Sci. Adv.* 8, eabm8149 (2022)). Accordingly, we believe that the statistical analysis of S0-S3 lifetimes is both scientifically rigorous and reliable.

We hope that the above explanation helps to clarify the reviewer's concern.

9. The definition of lifetime/dwell time used in the study is problematic. Dwell time should typically refer to the residence time in a specific state before transitioning to another state—not the distribution of all sojourn times within a given state. Based on the state transition matrix provided by the authors, there should be $4 \times 4 - 4 = 12$ distinct dwell times.

Response: We thank the reviewer for the comments. The reviewer's concern regarding the definition of dwell time may arise from a potential misinterpretation of the conductance histograms shown in the right panels of Figs 5a-c in the manuscript (also Figure R5 shown below). If that is the case, we would like to clarify that these histograms represent the stacking of current amplitudes (or conductance levels) on the Y axis, rather than the distributions of residence (sojourn) times. As the reviewer will see, these histograms are plotted in units of nA (current) or nS (conductance), and are used strictly to evaluate the distribution of distinct conductance states, from which the TN amplitudes (I_F) were extracted and subsequently analyzed in Figs. 5d and 5g. In contrast, dwell-time distributions were derived from HMM/QUB analysis of I-t trajectories (as exemplified in Fig. 5e), followed by monoexponential fitting to obtain the corresponding lifetimes (Fig. 5f).

Regarding the Definition of Dwell Time. Our definition is fully consistent with that described by the reviewer: it is the residence time that an individual enzyme molecule spends in a given state (e.g., S1, S2, or S3) before transitioning to another state (Fig. 5e and 5f). In this study, each reported lifetime represents a physically meaningful quantity corresponding to the temporal stability of a single-enzyme configuration prior to a stochastic transition event.

Regarding the Number of Dwell Times. We respectfully correct the reviewer that a 4 × 4 transition matrix should imply 12 possible transition pathways, rather than 12 distinct dwell times. As correctly noted by the reviewer, dwell time is defined as the residence time within a state. Each transition pathway (e.g., S0→S1, S1→S0, S0→S2, etc.) represents a stochastic exit event, which is effectively instantaneous on the timescale of our measurement (Fig. 5e). According to the principles of Markovian kinetics, dwell time is an intrinsic property of the state itself and does not depend on the specific exit pathway. In a single-molecule catalytic process, the time an enzyme resides in state S1 is characterized by a single observable lifetime (τ_1), regardless of whether the subsequent transition proceeds to S0, S2, or S3. Accordingly, reporting one lifetime per state, rather than separate lifetimes for each transition pathway, is the

established standard in multistate kinetic analysis using HMM methods, as supported by numerous biophysical studies (*Sci. Adv.* 8, eabm8149 (2022); *ACS Nano*, 18, 38, 25986-25996 (2024)). For example, in single-molecule studies of protein folding or reaction pathways, one typically reports the lifetimes of the "open" ("State 1") or "closed" ("State 2") state, not separate lifetimes for "open→closed1" ("State 1→State 2") and "open→closed2" ("State 2 →State 3") transition (*Nat Commun* 14, 552 (2023); *Nat. Nanotechnol.* 16, 1214-1223 (2021)).

We hope that the above explanation helps to clarify the reviewer's concern.

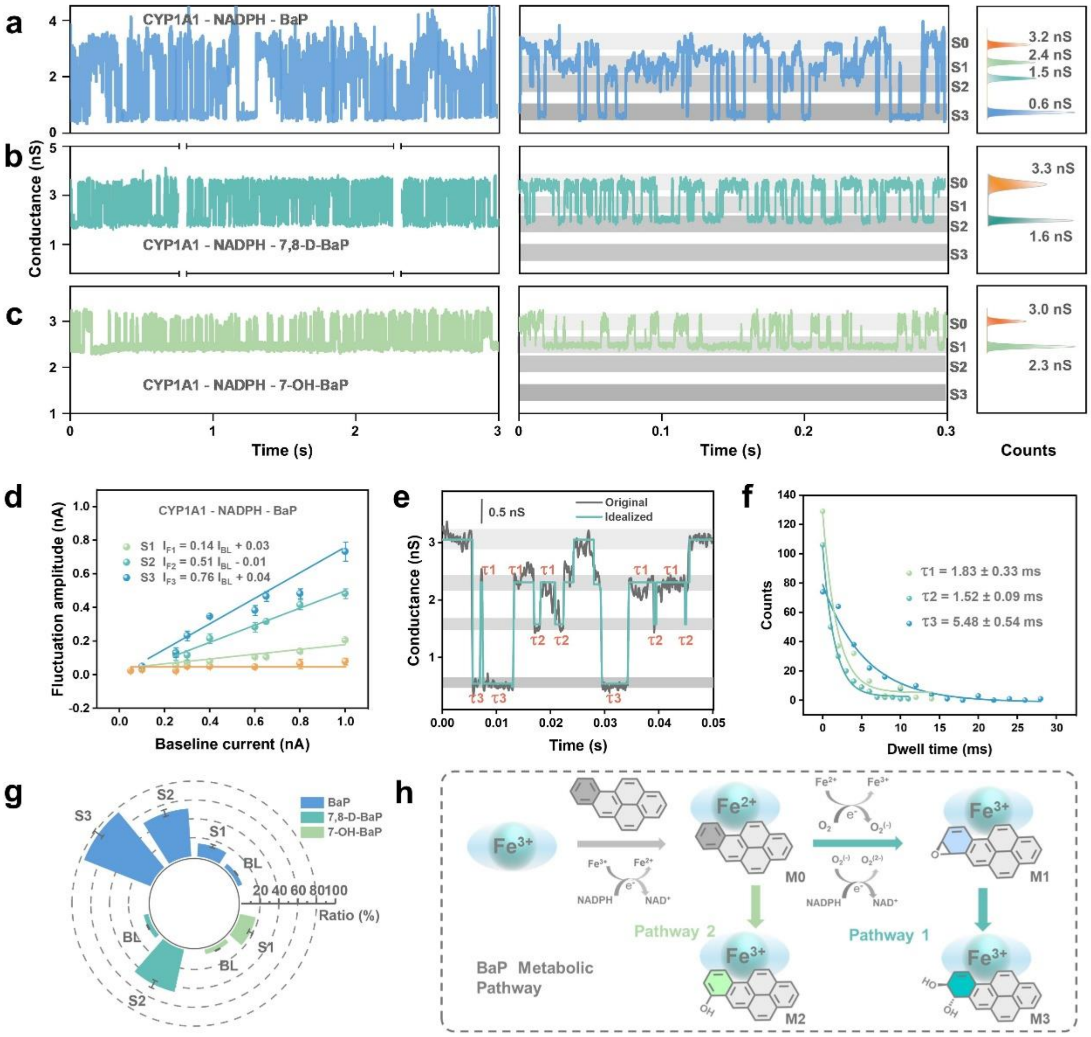

Figure R5. Monitoring catalytic dynamics of CYP1A1 based on conductance measurement. (a-c) Representative 3-s i-t traces during CYP1A1-mediated metabolism of BaP, 7,8-D-BaP, and 7-OH-BaP, respectively. The full traces (left), zoomed-in 0.3-s segments (middle), and corresponding conductance histograms (right) are shown. (d)

Dependence of TN amplitude (IF) on the baseline current (IBL). TN amplitude of S1, S2, and S3 exhibits an approximately linear correlation with the baseline current, with characteristic slopes for each state. (e) Example i–t trace segment idealized using a segmental k-means algorithm with hidden Markov modeling, fitted using QUB software (green trace). (f) Dwell time distributions for conductance states S1, S2, and S3 obtained from QUB fits. Lifetimes were extracted via monoexponential fitting. (g) Comparison of TN amplitude proportionality relative to baseline current for BaP and its metabolic intermediates. BL denotes baseline current fluctuation. (h) Proposed BaP metabolic pathways based on conductance measurements combined with HPLC-MS/MS analysis. Pathway 1 corresponds to metabolic activation; Pathway 2 corresponds to detoxification. The CYP1A1 heme active site is schematically represented by a blue ellipse.

10. The fitting of dwell time distributions is also unreliable. A proper dwell time distribution should follow a single-exponential decay. However, the authors appear to have constructed a duration histogram, which is expected to exhibit a bell-shaped distribution.

Response: We thank the reviewer for the opportunity to clarify our data presentation, as this concern appears to arise from a misinterpretation of the distinct types of histograms provided in Fig. 5. We must clarify that the histograms shown in the right panels of Figs. 5a-c represent the stacking of current amplitudes (or conductance levels) on the Y axis, rather than the distribution of residence (sojourn) times. This type of amplitude histogram is commonly used in the field of single-molecule electronics for visualizing current features (*Sci. Adv.* 8, eabm8149(2022)). As the reviewer will see, these histograms are plotted in units of nA (current) or nS (conductance), and are used strictly to evaluate the distribution of distinct conductance states, from which the TN fluctuations (I_F) were extracted and subsequently analyzed in Figs. 5d and 5g. In contrast, dwell-time distributions were derived from HMM/QUB analysis of I-t trajectories (as exemplified in Fig. 5e), followed by monoexponential fitting to obtain

the corresponding lifetimes (Fig. 5f).

As the reviewer correctly notes, for a memoryless, single-molecule Markovian process, the dwell-time distribution follows a single-exponential decay defined by $P(t) = k \cdot \exp(-kt)$, (*Nat Commun* 16, 9858 (2025)). Our temporal analysis is performed on the idealized traces generated through HMM and QUB (Fig. 5e). The resulting dwell-time distributions, presented in Fig. 5f, do indeed follow the expected monoexponential decay, confirming the stochastic nature of the transitions between catalytic states.

We hope that the above clarification adequately addresses the reviewer's concern. To further improve clarity, we have revised the figure labeling in the manuscript to avoid potential confusion.

11. For I-t measurements, please provide absolute conductance values instead of only scale bars. Absolute conductance is essential for assessing the stability and reliability of the measurements. Throughout the manuscript, I-t traces are presented with scale bars but without absolute values.

Response: We thank the reviewer for this valuable suggestion. As recommended, we have now included absolute conductance values for I-t traces (Fig. 5, also shown as Figure R6-R7). We fully agree that ensuring the stability and reliability of the measurements is essential. However, in single-molecule bioelectronic experiments, it is well recognized that the absolute baseline signal may vary due to differences in device configurations, STM probes, and local contact geometries. To address this inherent variability, we adopted the relative fluctuation amplitudes (%) for the identification of distinct conductance states, as shown in Fig. 5d. This normalization provides a more robust and reproducible metric and is widely employed in the field (*Nat. Methods* 22, 241253 (2025); *Acta Phys. Chim. Sin.* 12, 913 (2021)).

We thank the reviewer again for this helpful suggestion.

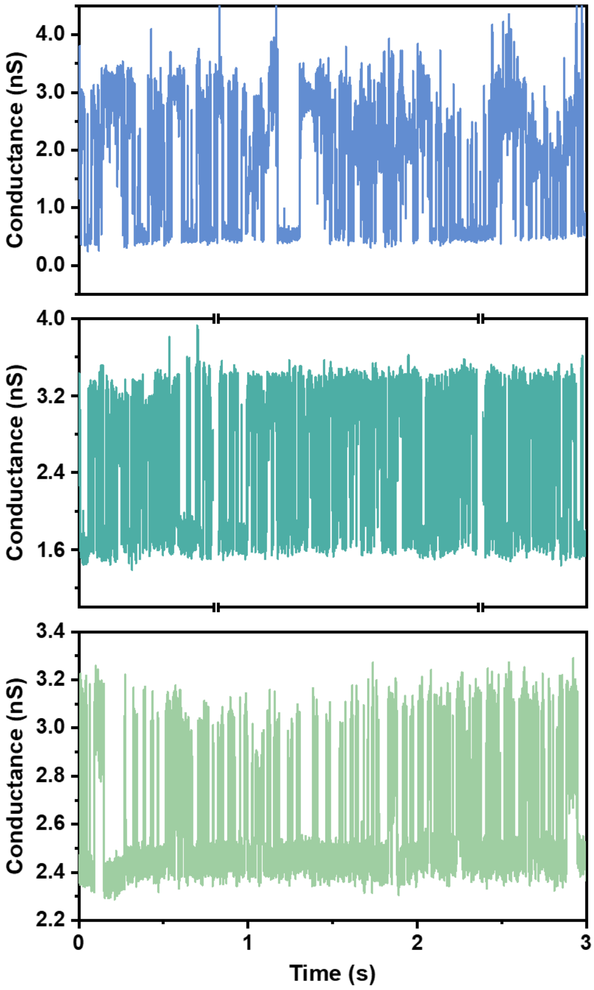

Figure R6. Representative 3-s i-t traces recorded during CYP1A1-mediated metabolism of BaP, 7,8-D-BaP, and 7-OH-BaP, respectively.

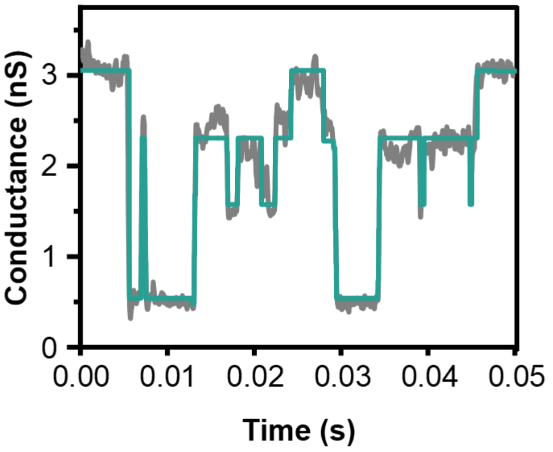

Figure R7. Example i-t trace segment idealized using a segmental k-means algorithm with hidden Markov modeling, fitted using QUB software (green trace).

12. Absolute conductance values should be reported for the I-t measurements. Do the absolute conductance values observed in single-state measurements fall within the amplitude range of the high-amplitude state?

Response: We thank the reviewer for this question. The answer is yes. As shown in Figures R6-R7, the baseline conductance (S0) of the measured I-t traces showing TN features mostly ranges from 0.5 to 5 nS, which falls within the conductance range corresponding to Peaks II and III obtained from our I-V histograms.

Reviewer #3 (Remarks to the Author):

General Comments: The authors have addressed all the points raised in my previous report. In my opinion, the paper only needs to improve the description of the methods employed, at least regarding the computational simulations.

Response: We sincerely thank the reviewer for the positive evaluation and constructive feedback. In response to this suggestion, we have revised and expanded the Methods section, particularly the description of the computational simulations.

1. In the Methods section it is first stated that "ORCA 6.0.156 was used for all quantum-chemical calculations in this study", but later it is said that "CP4K 4045.160 was used for all first-principles calculations in this study". The authors should clarify which methods were employed for each type of calculation. In addition, they need to provide more details about which atoms were included in the QM region (in QM/MM calculations) and which atoms were considered in the QM calculations. A figure in the Supporting Information could be useful for this purpose. Finally, the authors should provide the key structures in a public repository to facilitate reproducibility.

Response: We sincerely thank the reviewer for the insightful comments and suggestions regarding the computational methods and presentation of our results. To address these points, we have carefully revised the manuscript, as detailed below.

Regarding the methods employed for the theoretical calculations. We apologize for the confused description in the previous version of the manuscript. We actually adopted two different software packages for distinct purposes. ORCA 6.0.1 was used for HOMO-LUMO calculations, while CP2K 2025.1 was used for transmission spectrum calculations. We have revised the Methods section in the manuscript.

Details of the QM region in QM/MM calculations. In the QM/MM calculations performed with ORCA, the QM region includes the iron ion and its first-coordination shell ligands, which are essential for the electronic structure of the active site. Specifically, the QM region comprises the iron atom (Fe), the side chains of coordinating residues (Cys, sulfur atom, and C_β), and the porphyrin ring. In total, the QM region contains approximately 86 atoms (Supplement Data 1). To help visualize the partitioning, we have added a new figure (Figure R8, also Fig. S33) in the Supporting Information that shows the QM region (ball-and-stick) and the MM region (transparent surface) of the enzyme system (We have now provided the complete list of coordinates and charge values for the point charges in the MM region in Supporting Data 2). In the QM/MM model, environmental polarization was accounted for via electrostatic embedding, and the severed covalent bonds at the QM/MM boundary were capped with hydrogen link atoms.

For the CP2K calculations, the entire simulation cell (including all atoms: Au, C, H, O, N, S, Fe) is treated quantum mechanically (Figure R9, also Fig. S34); no QM/MM partitioning is applied.

Availability of key structures for reproducibility. As suggested, we have now provided Cartesian coordinates of all optimized key structures (both the enzyme active site models and the metal electrode models) in XYZ format in the revised Supporting Information. In addition, we have deposited these structures in Supplemental Data 2.

We thank the reviewer again for the constructive feedback and suggestions.

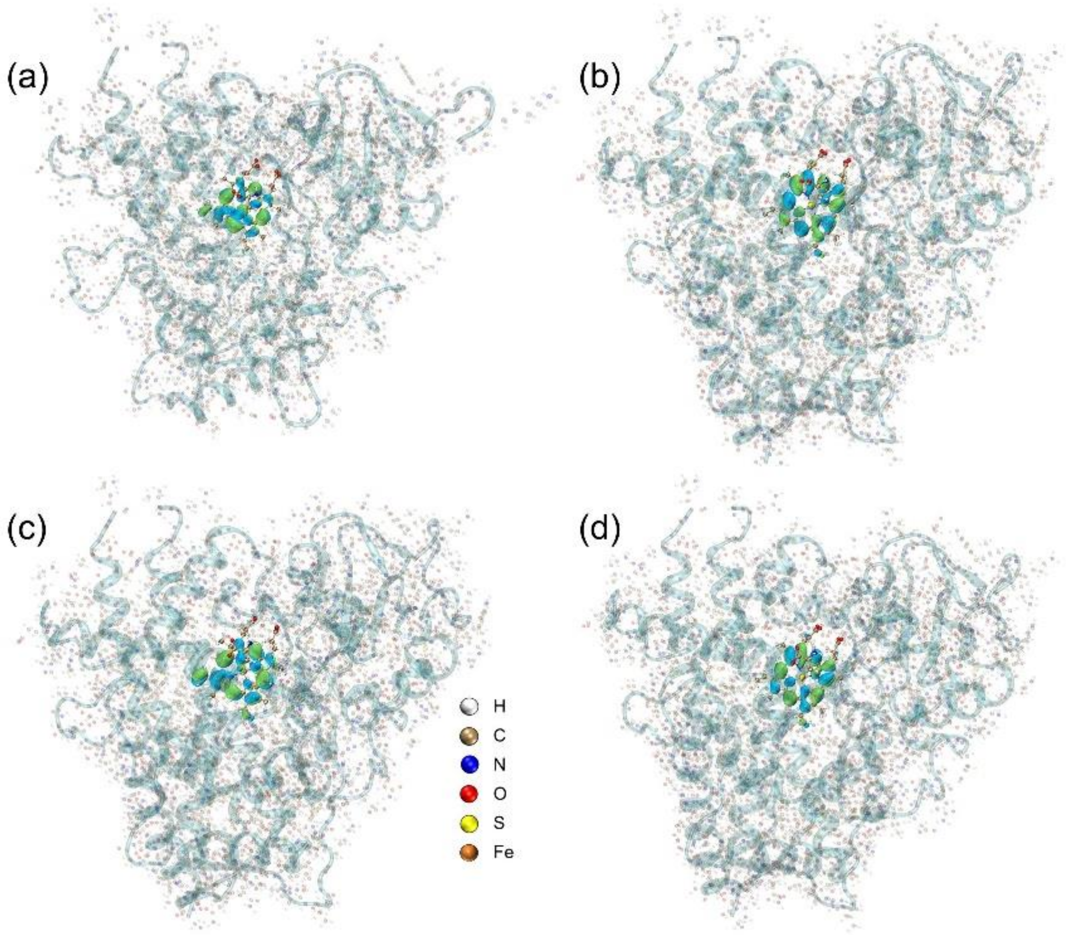

Figure R8. QM/MM computational model and frontier molecular orbital distributions of the iron-centered ligand. The HOMO and LUMO are shown for the Fe^{2+} (a, b) and Fe^{3+} (c, d) states. The central QM region (ball-and-stick) is embedded within the MM region, which is visualized as translucent background atoms and light blue protein ribbons.

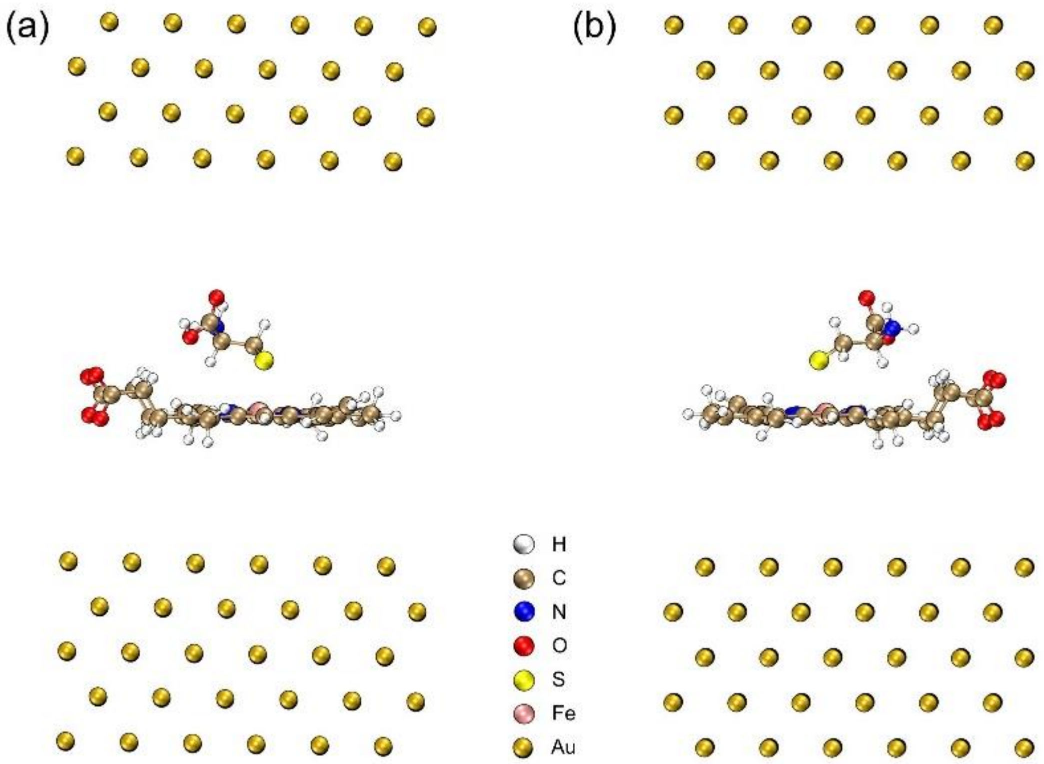

Figure R9. Two-probe molecular junction models used for transmission spectrum calculations. The central scattering region, comprising the Cys-porphyrin iron complex, is sandwiched between two semi-infinite gold electrodes (shown as spheres). Panels (a) and (b) depict the junction configurations for the Fe^{2+} and Fe^{3+} oxidation states, respectively.

Reviewer #4 (Remarks to the Author):

General Comments: I co-reviewed this manuscript with one of the reviewers who provided the listed reports. This is part of the Nature Communications initiative to facilitate training in peer review and to provide appropriate recognition for Early Career Researchers who co-review manuscripts.

Response: We gratefully acknowledge the reviewer's constructive comments and the time and effort dedicated to the evaluation of this manuscript.
